# Supplementary material for: Maternal dominance contributes to subgenome differentiation in allopolyploid fishes
Source: Nat Commun. 2023 Dec 15;14:8357. doi: 10.1038/s41467-023-43740-y (PMC10724154; doi:10.1038/s41467-023-43740-y)
Supplement: Supplementary file 1 — Supplementary information [file 41467_2023_43740_MOESM1_ESM.pdf]

Supplementary information for

Maternal dominance contributes to subgenome differentiation in allopolyploid fishes

Supplementary Figures 1-50

Supplementary Fig. 1. The estimation of genome size of 21 cyprinid fishes based on a genomic distribution of 17-mer frequencies.

Supplementary Fig. 2. Evidence for the allotetraploid origin of *S. sinensis*. **a.** Intensity signal heat map of the high-throughput chromatin conformation capture (Hi-C) chromosome interaction. **b.** Syntenic relationships between *O. macrolepis* and *S. sinensis* subP and subM. The green band showed one example of a collinearity gene between homologous chromosomes. **c.** Heatmap and clustering of differential k-mers. The x-axis, differential k-mers; y-axis, chromosomes. The vertical color bar, each chromosome is assigned to subP and subM; the horizontal color bar, each k-mer is specific to subP and subM. **d.** TE frequency on chromosomes showing subP and subM biased distributions in the tetraploid genome of *S. sinensis*.

Supplementary Fig. 3. Evidence for the allotetraploid origin of *P. rabaudi*. Evidence for the allotetraploid origin of *P. rabaudi*. **a.** Intensity signal heat map of the high-throughput chromatin conformation capture (Hi-C) chromosome interaction. **b.** Syntenic relationships between *O. macrolepis* and *P. rabaudi* subP and subM. The green band showed one example of a collinearity gene between homologous chromosomes. **c.** Heatmap and clustering of differential k-mers. The x-axis, differential k-mers; y-axis, chromosomes. The vertical color bar, each chromosome is assigned to subP and subM; the horizontal color bar, each k-mer is specific to subP and subM. **d.** TE frequency on chromosomes showing subP and subM biased distributions in the tetraploid genome of *P. rabaudi*.

Supplementary Fig. 4. Heatmap of intensity signals of per chromosome Hi-C interactions in allotetraploid *L. capito*.

Supplementary Fig. 5. Heatmap of intensity signals of per chromosome Hi-C interactions in allotetraploid *P. rabaudi*.

Supplementary Fig. 6. Heatmap of intensity signals of per chromosome Hi-C interactions in allotetraploid *S. sinensis*.

Supplementary Fig. 7. The correlated relationship between genome size and TEs in 21 cyprinids using the spearman method. TE content in subP and subM of *P. rabaudi*, *L. capito* and *S. sinensis* were shown using green, blue and red solid circle, respectively.

Supplementary Fig. 8. Summary of TE age across species. Heatmap depicts the median age (across all insertions) of given TE classes (DNA, LTR, SINE, LINE, RC, and others) and TE superfamilies.

Supplementary Fig. 9. Putative homeobox genes identified in the assembly of *P. rabaudi*, *S. sinensis* and *L. capito*.

Supplementary Fig. 10. Principal component analysis (PCA) of differential k-mers in *L. capito*, *P. rabaudi*, and *S. sinensis*, respectively. Points indicate

chromosomes.

Supplementary Fig. 11. The distribution of subgenome-biased index (SBI) for TEs in the reference genome of three allotetraploids.

Supplementary Fig. 12. Divergence times within the Cyprinidae family and between subgenomes of five allotetraploids inferred by MCMCTree. Species tree constructed using IQ-TREE based on CDS of 300 one-to-one orthologues. *Triplophysa bleekeri* was used as the outgroup. See Supplementary Fig. 13.

Supplementary Fig. 13. A Maximum Likelihood (ML) phylogenetic tree of the Cyprinidae family on the basis of CDS of 300 one-to-one orthologues using IQ-TREE. *T. bleekeri* was used as the outgroup. Bootstrap values supporting on each node are calculated with 1000 replicates.

Supplementary Fig. 14. A Maximum Likelihood (ML) phylogenetic tree of the Cyprinidae family on the basis of CDS of 310 one-to-one orthologues using RAxML. Zebrafish and *Danionella translucida* were used as the outgroup.

Supplementary Fig. 15. A phylogeny of the Cyprinidae family based on mitochondria genomes. We used mitochondrial genomes of 37 species to infer this phylogeny by RAxML using *T. bleekeri* as the outgroup. Bootstrap values lower than 60 were not shown.

Supplementary Fig. 16. A ML tree made from the whole genome alignment (WGA). The WGA of 13 cyprinid fishes was used for building the ML phylogenetic tree by RAxML. Numbers on the nodes represent the support values from 200 bootstrap tests. We used *Distoechodon tumirostris* as the outgroup.

Supplementary Fig. 17. A ML phylogenetic tree generated from 4d sites. 252,437 4d sites of 1,669 single-copy orthologs from 13 cyprinid fishes were identified and used for constructing a ML tree by RAxML. Numbers on the nodes represent the support values from 200 bootstrap tests. *D. tumirostris* was used as the outgroup.

Supplementary Fig. 18. a Summary tree across 1665 genetrees, inferred by SumTrees. Values represent the degree of support for clades indicated as proportions (posterior probabilities). **b** DensiTree of 1665 genetrees, constructed using MUSCLE and RaxML-NG; green: concensus trees across multiple gene trees; blue: summary tree.

Supplementary Fig. 19. Congruences between concatenation-based tree (left) and summary tree across 1665 genetrees (right).

Supplementary Fig. 20. Examples of expression of sub-F (left) and neo-F (right) genes. Gray bar, *Sc. acanthopterus* or *O. macrolepis*; blue bar, subP of each allotetraploid; red bar, subM of each allotetraploid. Tissues related with sub-F or neo-F were showed using asterisks.

Supplementary Fig. 21. Subgenome fractionation of *L. capito* chromosomes relative to the diploid *Danio rerio*. Gene retention in *L. capito* subP (red) and subM

(blue) was calculated in 100 gene sliding windows for each chromosome of the *D. rerio* reference.

Supplementary Fig. 22. Subgenome fractionation of *P. rabaudi* chromosomes relative to the diploid *D. rerio*. Gene retention in *P. rabaudi* subP (red) and subM (blue) was calculated in 100 gene sliding windows for each chromosome of the *D. rerio* reference.

Supplementary Fig. 23. Subgenome fractionation of *S. sinensis* chromosomes relative to the diploid *D. rerio*. Gene retention in *S. sinensis* subP (red) and subM (blue) was calculated in 100 gene sliding windows for each chromosome of the *D. rerio* reference.

Supplementary Fig. 24. Subgenome fractionation of *L. capito* chromosomes relative to the diploid *O. macrolepis*. Gene retention in *L. capito* subP (red) and subM (blue) was calculated in 100 gene sliding windows for each chromosome of the *O. macrolepis* reference.

Supplementary Fig. 25. Subgenome fractionation of *P. rabaudi* chromosomes relative to the diploid *O. macrolepis*. Gene retention in *P. rabaudi* subP (red) and subM (blue) was calculated in 100 gene sliding windows for each chromosome of the *O. macrolepis* reference.

Supplementary Fig. 26. Subgenome fractionation of *S. sinensis* chromosomes relative to the diploid *O. macrolepis*. Gene retention in *S. sinensis* subP (red) and subM (blue) was calculated in 100 gene sliding windows for each chromosome of the *O. macrolepis* reference.

Supplementary Fig. 27. Subgenome fractionation of *L. capito* chromosomes relative to the diploid *Sc. acanthopterus*. Gene retention in *L. capito* subP (red) and subM (blue) was calculated in 100 gene sliding windows for each chromosome of the *Sc. acanthopterus* reference.

Supplementary Fig. 28. Subgenome fractionation of *P. rabaudi* chromosomes relative to the diploid *Sc. acanthopterus*. Gene retention in *P. rabaudi* subP (red) and subM (blue) was calculated in 100 gene sliding windows for each chromosome of the *Sc. acanthopterus* reference.

Supplementary Fig. 29. Subgenome fractionation of *Sc. sinensis* chromosomes relative to the diploid *S. acanthopterus*. Gene retention in *S. sinensis* subP (red) and subM (blue) was calculated in 100 gene sliding windows for each chromosome of the *Sc. acanthopterus* reference.

Supplementary Fig. 30. Biased distribution of complete and single copy genes generated by the BUSCO analysis in the subM of five allotetraploids (X^2 text; p-value≤5.35e-6).

Supplementary Fig. 31. GO enrichment analysis of the complete and single copy BUSCO genes in the subP of *S. sinensis*.

Supplementary Fig. 32. GO enrichment analysis of the complete and single copy BUSCO genes in the subM of *S. sinensis*.

Supplementary Fig. 33. GO enrichment analysis of the complete and single copy BUSCO genes in the subP of *P. rabaudi*.

Supplementary Fig. 34. GO enrichment analysis of the complete and single copy BUSCO

genes in the subM of *P. rabaudi*.

Supplementary Fig. 35. GO enrichment analysis of the complete and single copy BUSCO genes in the subP of *L. capito*.

Supplementary Fig. 36. GO enrichment analysis of the complete and single copy BUSCO genes in the subM of *L. capito*.

Supplementary Fig. 37. Pan-gene analysis. **a** Frequency of orthogroups. The pie chart shows the proportion of core (shared by all 36 samples), softcore (shared by > 90% samples but not all), dispensable (shared by more than one but ≤ 90% samples), and private genes (present in only one sample) in those genomes. **b** Number of core genes that exists only in subP and subM of allopolyploids. There tends to be a statistically significant number of core genes biased distribution toward the subM in all species except *P. rabaudi* (X^2 text; p-value≤4.6e-4).

Supplementary Fig. 38. Median TPM of genes from three to five replicates of RNAseq across six tissue types in *S. sinensis*, *L. capito*, and *P. rabaudi*. Median TPM values displayed are separated by subP (red) and subM (blue) with the average across all three to five replicates for each subgenome/tissue indicated by horizontal black bars.

Supplementary Fig. 39. Expression of homoeolog pairs plotted as subP biased synteologs (red), subM biased syntelogs (blue) and unbiased synteologs (grey) was shown in five tissues.

Supplementary Fig. 40. Histograms of differences in TE density values downstream of subP and subM syntelogs of *S. sinensis*, *L. capito*, and *P. rabaudi*. The density values were calculated for all TEs in a 10,000 bp window downstream of genes and difference values were calculated by subtracting TE density of subM syntelogs from subP syntelogs. Negative values represent higher TE density for syntelogs in the subM, whereas positive values reflect higher TE density for the subP syntelogs.

Supplementary Fig. 41. Boxplots of TE density values for genes exhibiting biased expression in subP (left column) and subM (right column). TE density of syntelogs in subP (red) and subM (blue) for *S. sinensis*, *L. capito*, and *P. rabaudi* were plotted for 10,000 bp windows upstream of gene start sites. Two-sample t-test results are shown as non-significant (ns), p < 0.005 (**) and p < 0.00005 (****).

Supplementary Fig. 42. Boxplots of TE density values for genes exhibiting biased expression in subP (left column) and subM (right column). TE density of syntelogs in subP (red) and subM (blue) for *S. sinensis*, *L. capito*, and *P. rabaudi* were plotted for 10,000 bp windows downstream of gene start sites. Two-sample t-test results are shown as non-significant (ns) and p < 0.00005 (****).

Supplementary Fig. 43. Methylation levels at the CH sites of two diploid ancestors *O. macrolepis* and *Sc. acanthopterus* and three allotetraploids *S. sinensis*, *L. capito*, and *P. rabaudi*. The x axis represented the gene

body (TSS = transcription start site and TTS = transcription termination site) and 2 kb upstream and downstream region. The y axis showed the weighted CH methylation level.

Supplementary Fig. 44. CG methylation pattern of two diploid ancestors *O. macrolepis* and *Sc. acanthopterus* and three allotetraploids *S. sinensis*, *L. capito*, and *P. rabaudi*. The x axis showed the gene body (TSS and TTS) and 2 kb upstream and downstream region. The y axis was the weighted CG methylation level.

Supplementary Fig. 45. CG methylation pattern of 7040 genes with a 1:1:2:2:2 relationship (1 *O. macrolepis* gene, 1 *Sc. acanthopterus* gene, 2 *S. sinensis* genes, 2 *L. capito* genes and 2 *P. rabaudi* genes). The x and y axis represented the gene body (TSS and TTS) and 2 kb upstream and downstream region and weighted CG methylation level, respectively.

Supplementary Fig. 46. CG methylation levels of subP (**a**) or subM (**b**) biased expression genes in the muscle tissue of *L. capito*, *P. rabaudi* and *S. sinensis*. The x axis was the gene body and 2 kb upstream and downstream region. The y axis indicated the weighted CG methylation level.

Supplementary Fig. 47. Comparision of TE mCG levels between subP (red) and subM (green). CG methylation of TEs that are in 1kb vicinity of 7040 positionally conserved syntenic ohnologs (**a**) and at the whole genome level (**b**). The x axis was the TE and 2 kb upstream and downstream region. The y axis indicated the weighted CG methylation level of TEs.

Supplementary Fig. 48. Global A/B compartments identified in the *L. capito* genome using the PCA-based method.

Supplementary Fig. 49. Global A/B compartments found in the *P. rabaudi* genome using the PCA-based method.

Supplementary Fig. 50. Global A/B compartments obtained from the *S. sinensis* genome using the PCA-based method.

Supplementary Tables 1-24

Supplementary Table 1. Detail information of sequenced species in this study.

Supplementary Table 2. Summary of sequencing data for assembly of the genomes.

Supplementary Table 3. Summary of genome assemblies of twenty-one species.

Supplementary Table 4. Estimation of the genome size using 17-mer distribution analysis.

Supplementary Table 5. BUSCO evaluation of the completeness and accuracy of the genomes.

Supplementary Table 6. Statistics of Hi-C mapping of the *S. sinensis* genome.

Supplementary Table 7. Statistics of Hi-C mapping of the *L. capito* genome.

Supplementary Table 8. Statistics of Hi-C mapping of the *P. rabaudi* genome.

Supplementary Table 9. Statistical data of 50 chromosomes of *S. sinensis*.

Supplementary Table 10. Statistical data of 50 chromosomes of *P. rabaudi*.

Supplementary Table 11. Statistical data of 50 chromosomes of *L. capito*.

Supplementary Table 12. BUSCO evaluation of gene completeness from the assembly

genomes.

Supplementary Table 13. Characteristics of transposable element identified in the assembled genomes.

Supplementary Table 14. Transposble elements that are subgenome biased in the three allotetraploids.

Supplementary Table 15. Number of ohnolog clusters in evolutionary fate categories.

Supplementary Table 16. Total subgenome percent gene retention values for each subgenome of three focal tetraploid species when aligned to three diploid references.

Supplementary Table 17. Detail information of tandem repeat genes of *S. sinensis*, *L. capito* and *P. rabaudi*.

Supplementary Table 18. Chi-squared test (two-sided) results for biased genes in each of six tissue types.

Supplementary Table 19. Chi-squared test (two-sided) results for biased genes in six tissue types which are retained in a 1:1:2:2:2 ratio across subgenomes and the three focal allotetraploids.

Supplementary Table 20. Constraint on CNSs located on the subP and subM of each species relative to a model that incorporates mean CNS conservation across all subgenomes and subgenome-specific phylogenetic distance from *O. macrolepis*.

Supplementary Table 21. Non-clonal read pair alignment rate and non-conversion rate of the two diploid and three tetraploid fish species.

Supplementary Table 22. TAD and conserved TAD identified by hicFINDTAD and HiTAD.

Supplementary Table 23. Change in size of TADs found in subgenomes of three allotetraploids.

Supplementary Table 24. Mitochondrial and assembly genomes of reported species used in this study.

Supplementary Data 1-6 provided as separate Excel files

Supplementary Data 1. Evaluation of the accuracy of the assembled genomes using Illumina pair-end reads.

Supplementary Data 2. Characteristics of genes identified in the assembled genomes.

Supplementary Data 3. Detail information of TEs identified in the assembled genomes.

Supplementary Data 4. Hox genes identified in subgenomes of three allotetraploids.

Supplementary Data 5. Occupied genome length, gene number and TE sequences in A/B compartment in subgenomes of three allotetraploids.

Supplementary Data 6. Tissues of studied species used for RNA-Seq and Iso-Seq.

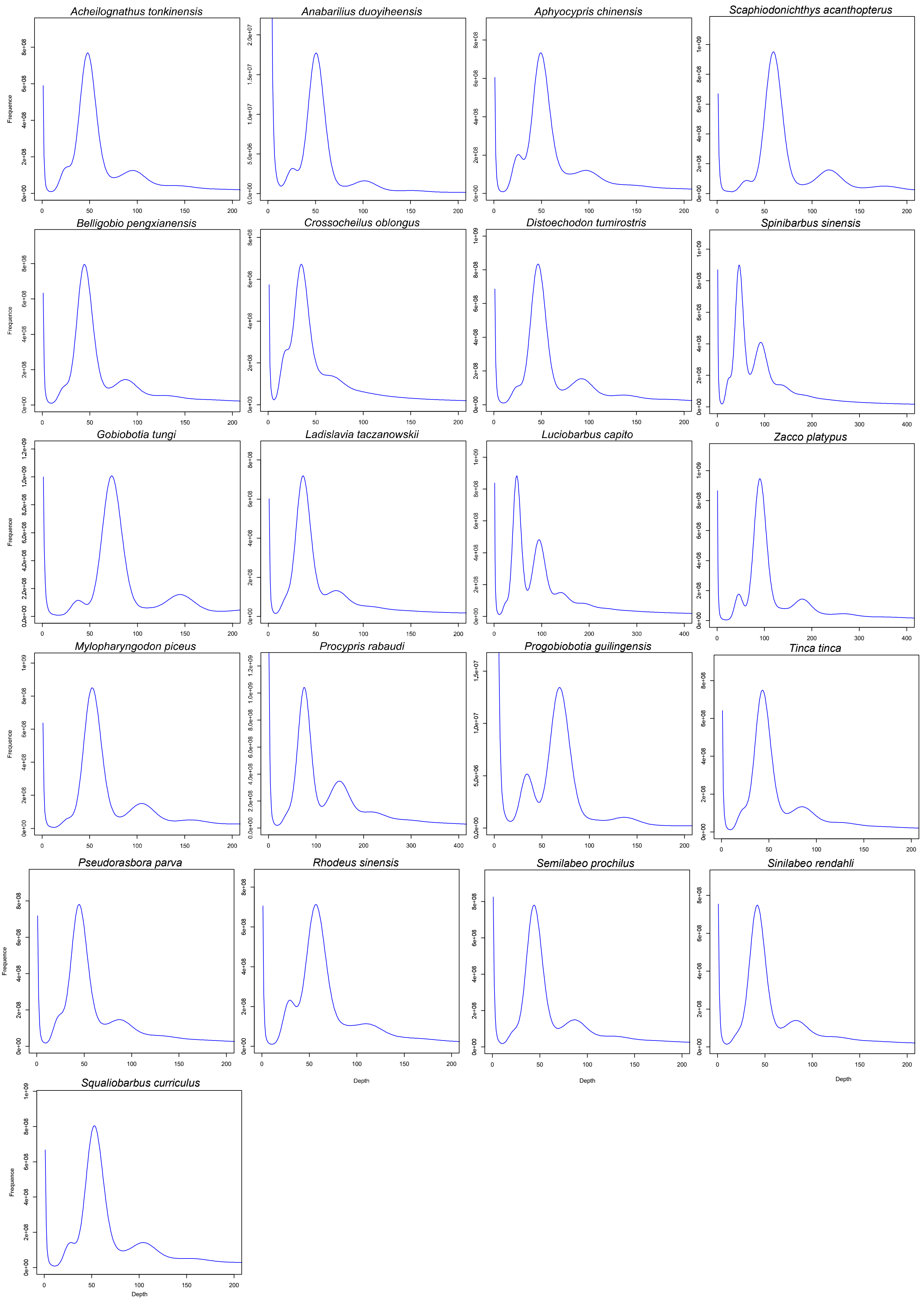

Supplementary Fig. 1. The estimation of genome size of 21 cyprinid fishes based on a genomic distribution of 17-mer frequencies.

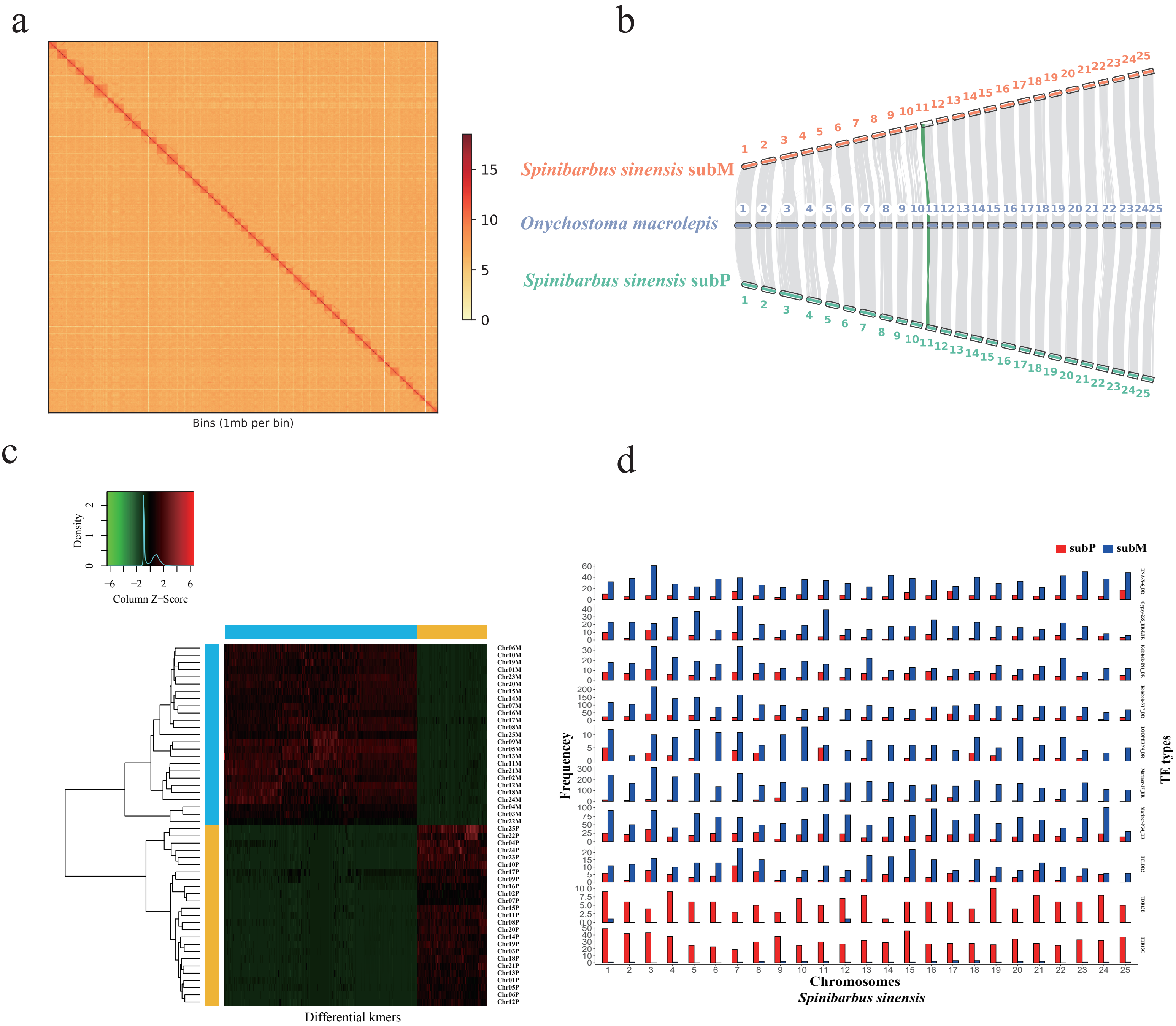

Supplementary Fig. 2. Evidence for the allotetraploid origin of *S. sinensis*. **a.** Intensity signal heat map of the high-throughput chromatin conformation capture (Hi-C) chromosome interaction. **b.** Syntenic relationships between *O. macrolepis* and *S. sinensis* subP and subM. The green band showed one example of a collinearity gene between homologous chromosomes. **c.** Heatmap and clustering of differential k-mers. The x-axis, differential k-mers; y-axis, chromosomes. The vertical color bar, each chromosome is assigned to subP and subM; the horizontal color bar, each k-mer is specific to subP and subM. **d.** TE frequency on chromosomes showing subP and subM biased distributions in the tetraploid genome of *S. sinensis*.

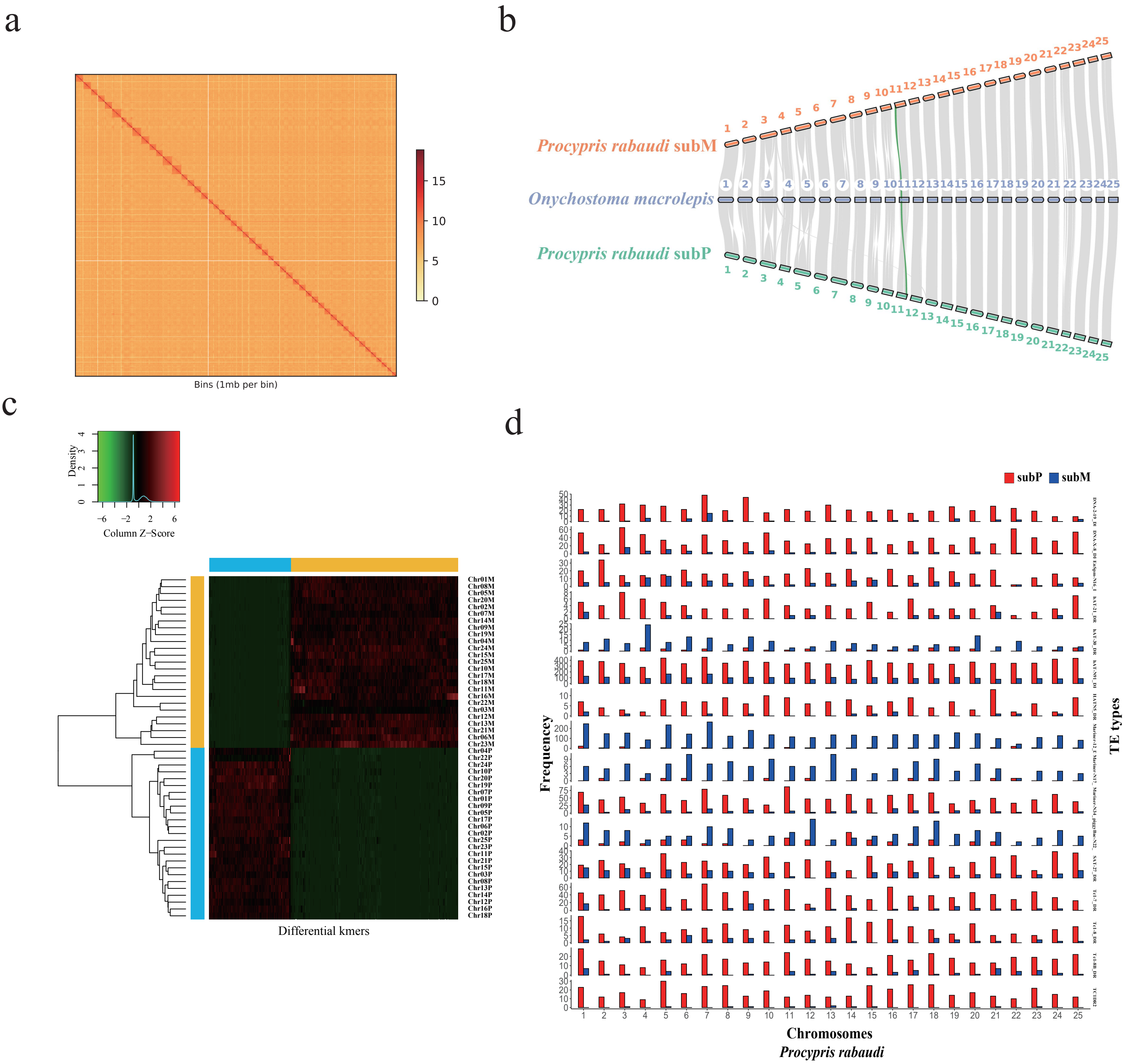

Supplementary Fig. 3. Evidence for the allotetraploid origin of *P. rabaudi*. Evidence for the allotetraploid origin of *P. rabaudi*. **a.** Intensity signal heat map of the high-throughput chromatin conformation capture (Hi-C) chromosome interaction. **b.** Syntenic relationships between *O. macrolepis* and *P. rabaudi* subP and subM. The green band showed one example of a collinearity gene between homologous chromosomes. **c.** Heatmap and clustering of differential k-mers. The x-axis, differential k-mers; y-axis, chromosomes. The vertical color bar, each chromosome is assigned to subP and subM; the horizontal color bar, each k-mer is specific to subP and subM. **d.** TE frequency on chromosomes showing subP and subM biased distributions in the tetraploid genome of *P. rabaudi*.

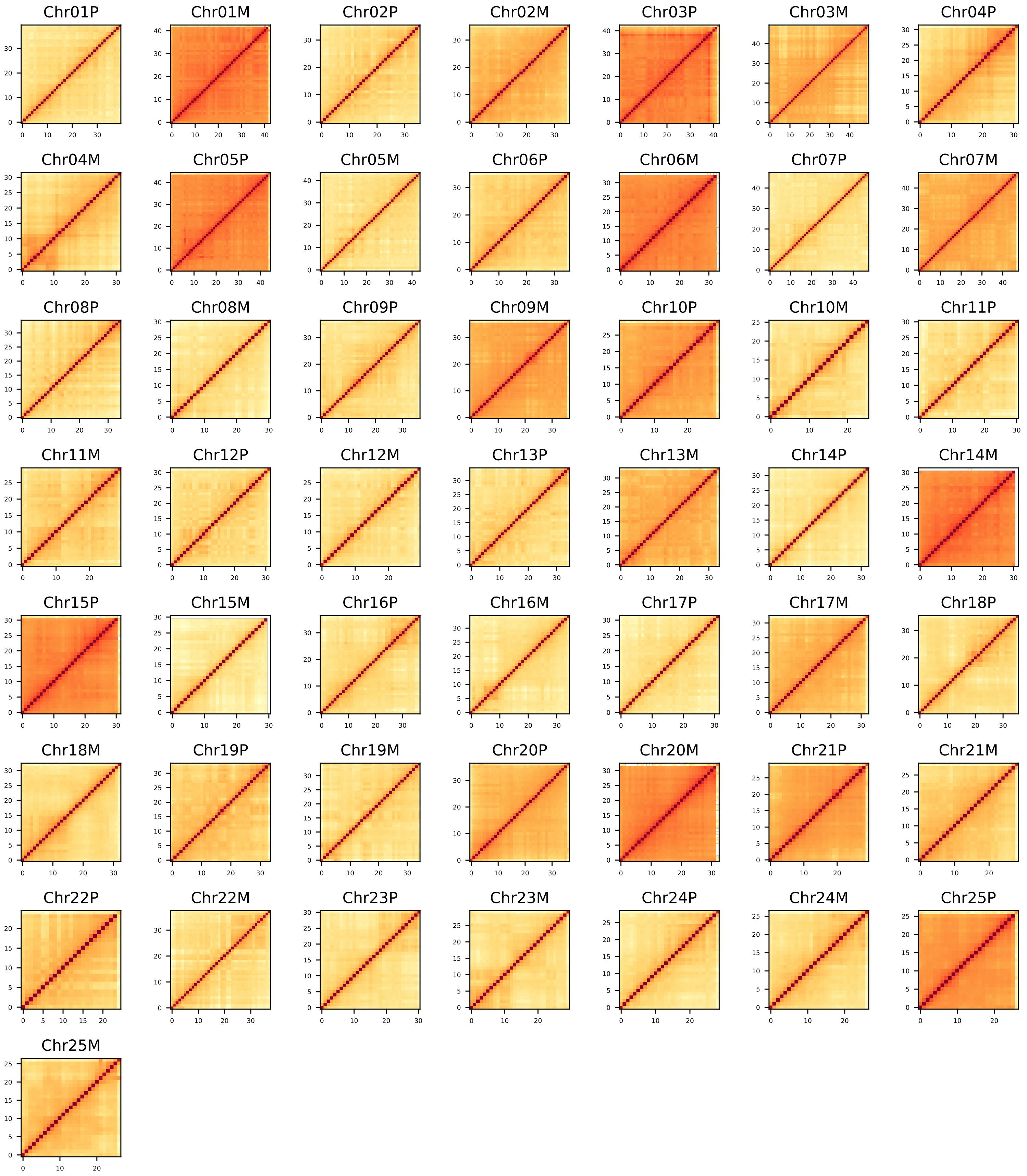

Supplementary Fig. 4. Heatmap of intensity signals of per chromosome Hi-C interactions in allotetraploid *L. capito.*

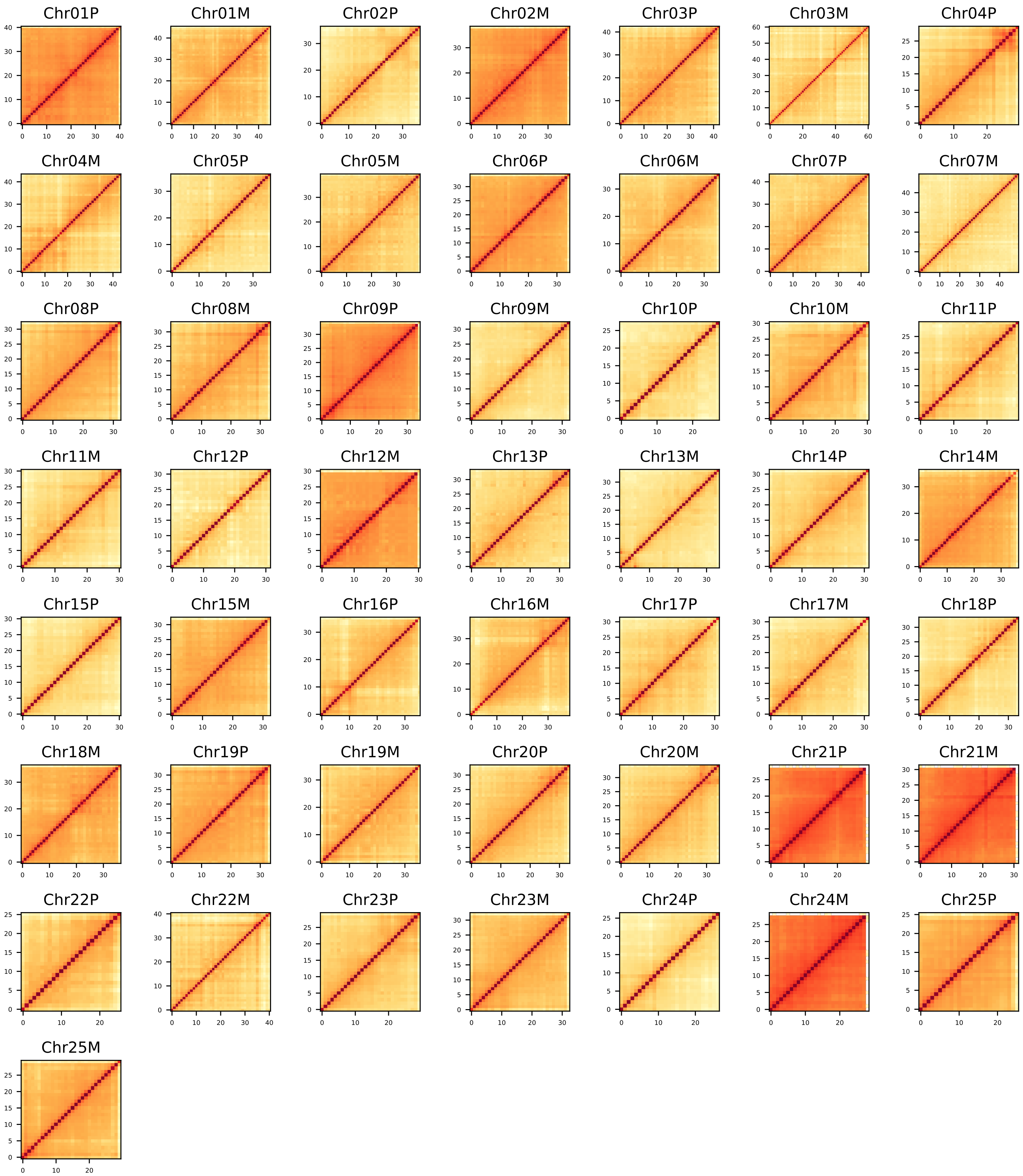

Supplementary Fig. 5. Heatmap of intensity signals of per chromosome Hi-C interactions in allotetraploid *P. rabaudi*.

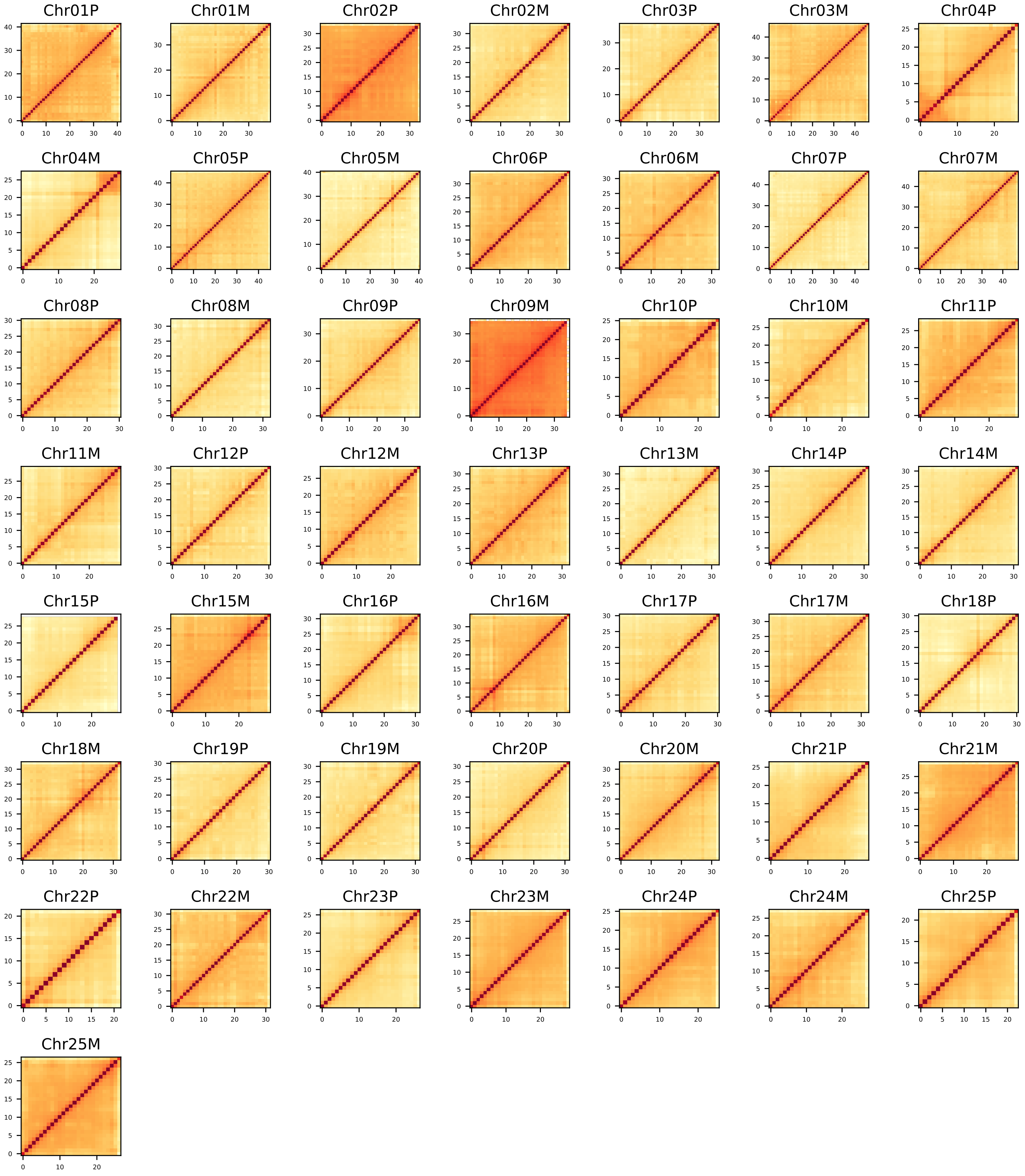

Supplementary Fig. 6. Heatmap of intensity signals of per chromosome Hi-C interactions in allotetraploid *S. sinensis*.

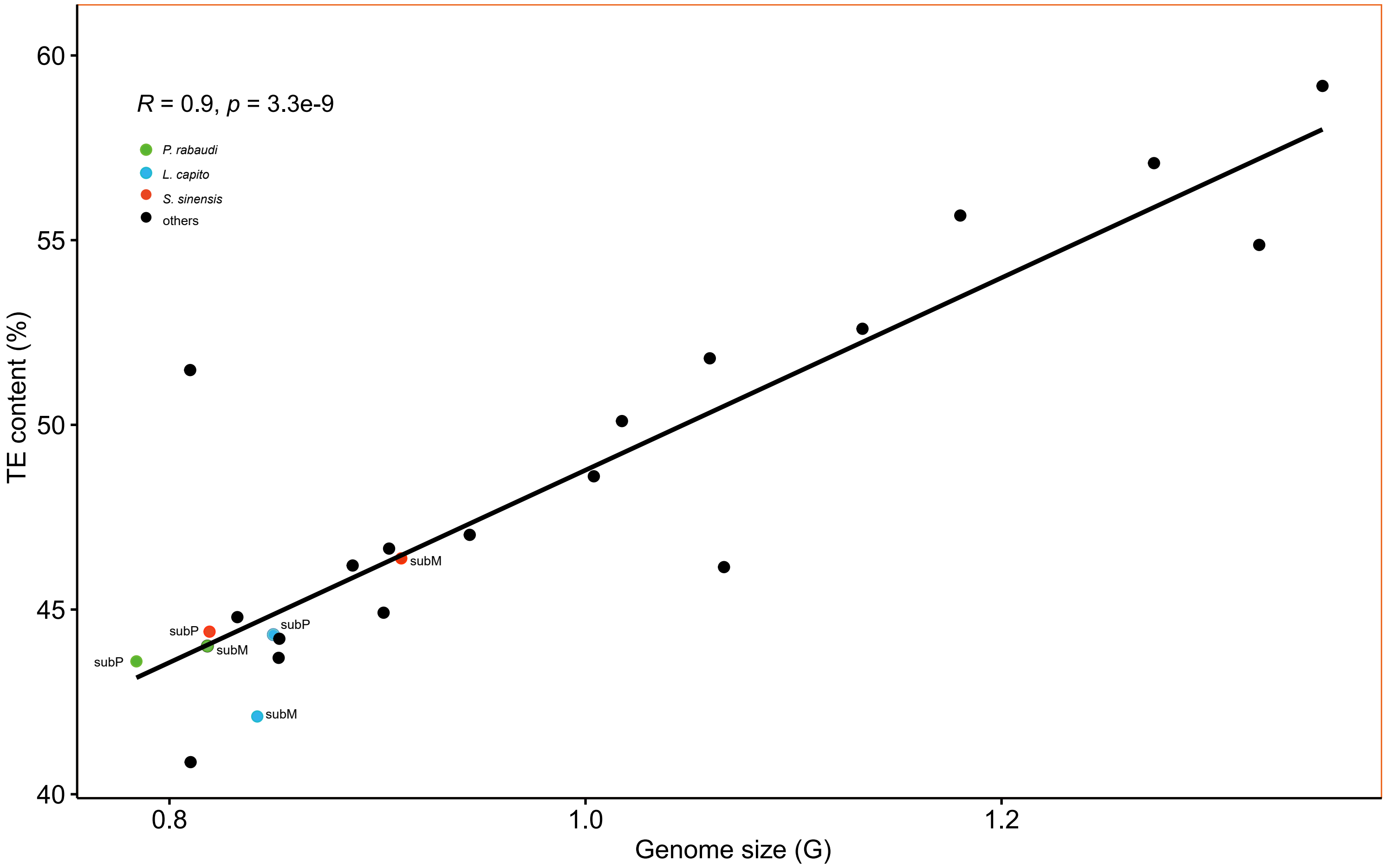

Supplementary Fig. 7. The correlated relationship between genome size and TEs in 21 cyprinids using the spearman method. TE content in subP and subM of *P. rabaudi*, *L. capito* and *S. sinensis* were shown using green, blue and red solid circle, respectively.

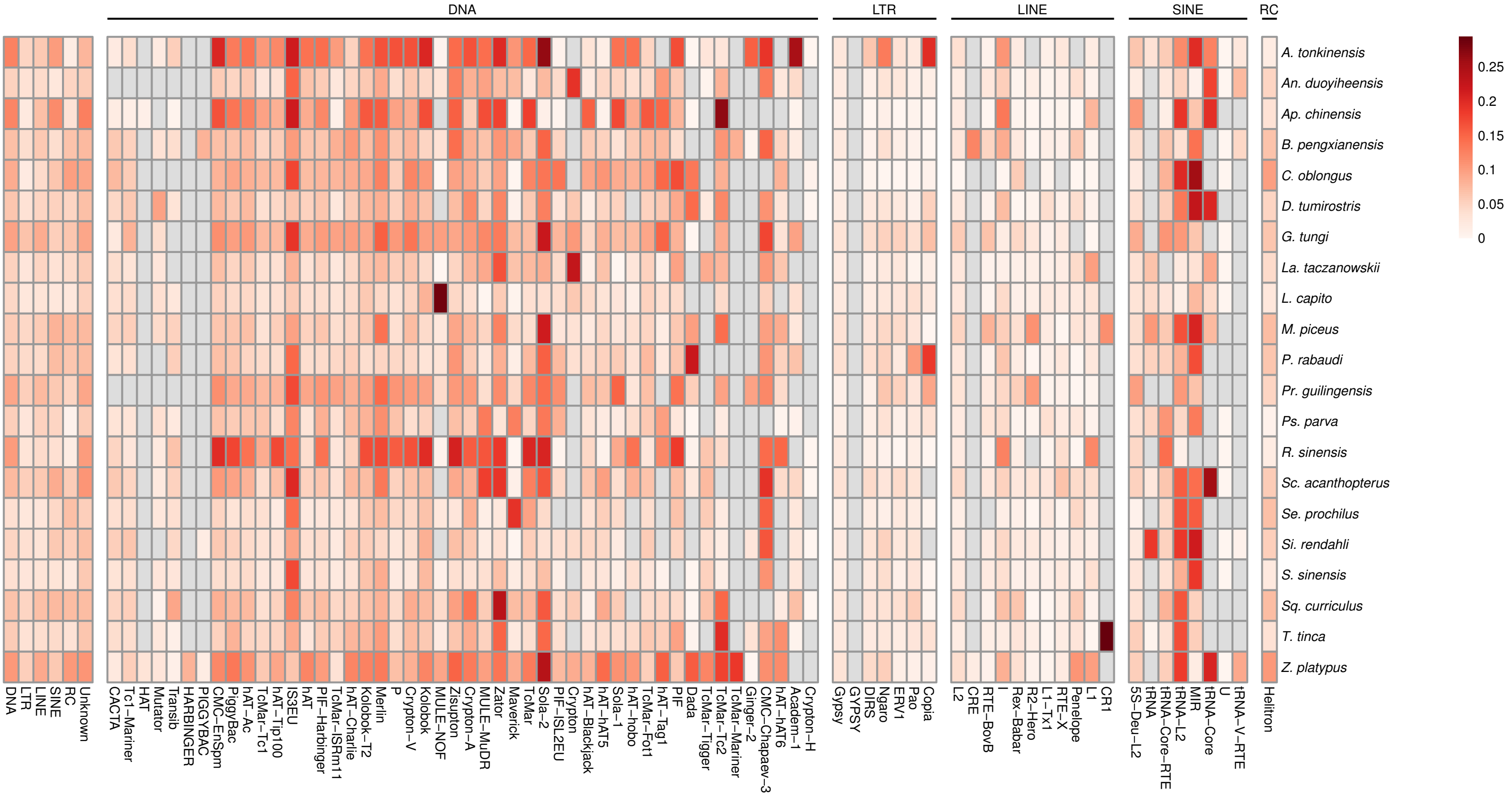

Supplementary Fig. 8. Summary of TE age across species. Heatmap depicts the median age (across all insertions) of given TE classes (DNA, LTR, SINE, LINE, RC, and others) and TE superfamilies.

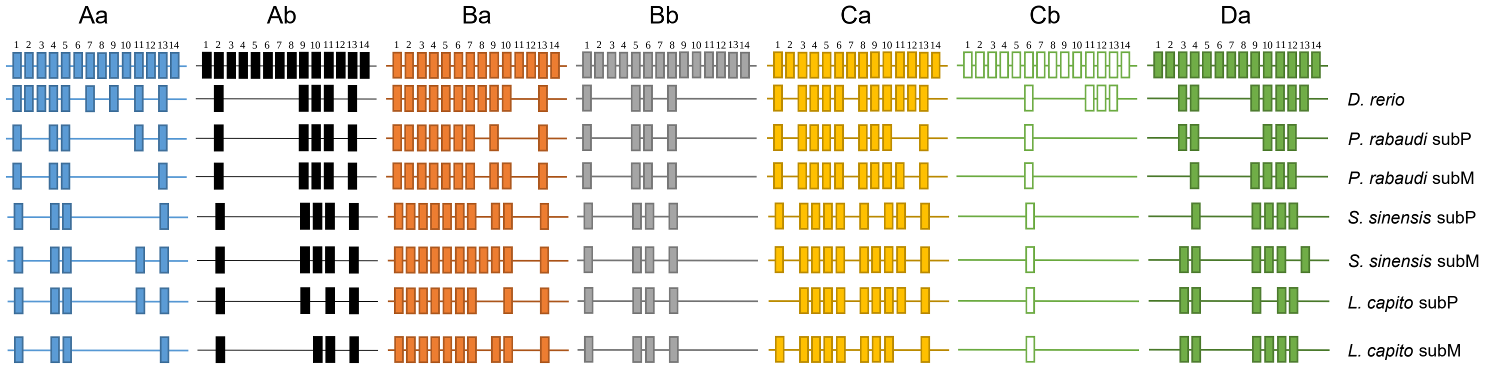

Supplementary Fig. 9. Putative homeobox genes identified in the assembly of *P. rabaudi*, *S. sinensis* and *L. capito*.

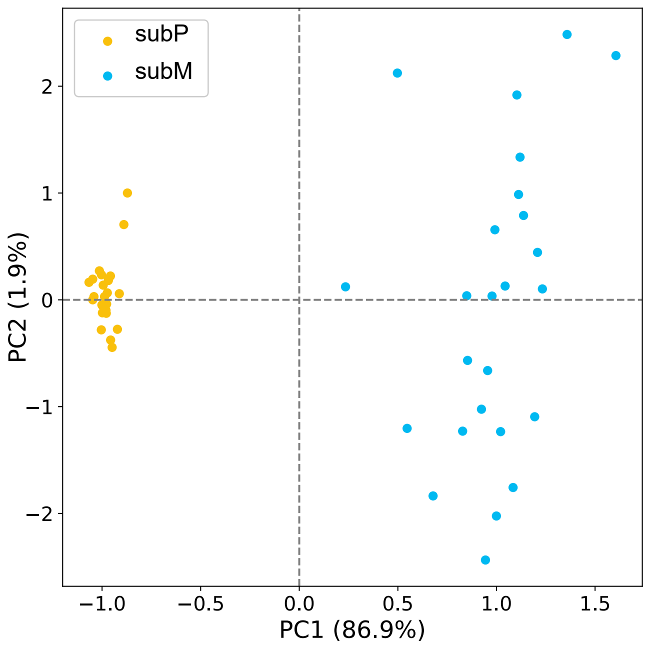

L. capito

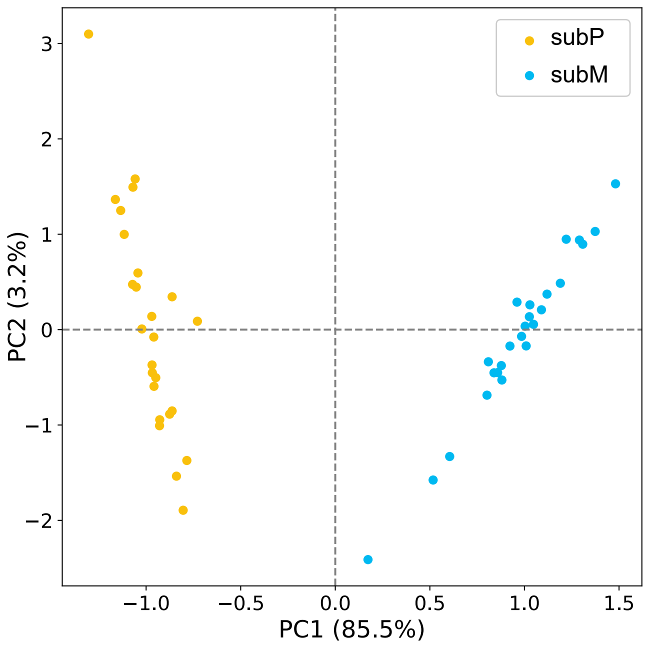

S. sinensis

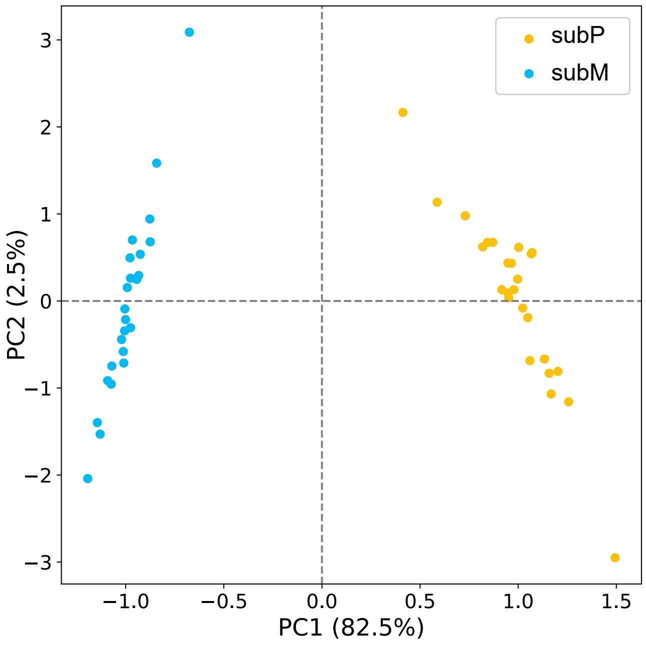

P. rabaudi

Supplementary Fig. 10. Principal component analysis (PCA) of differential k-mers in *L. capito*, *P. rabaudi*, and *S. sinensis*, respectively. Points indicate chromosomes.

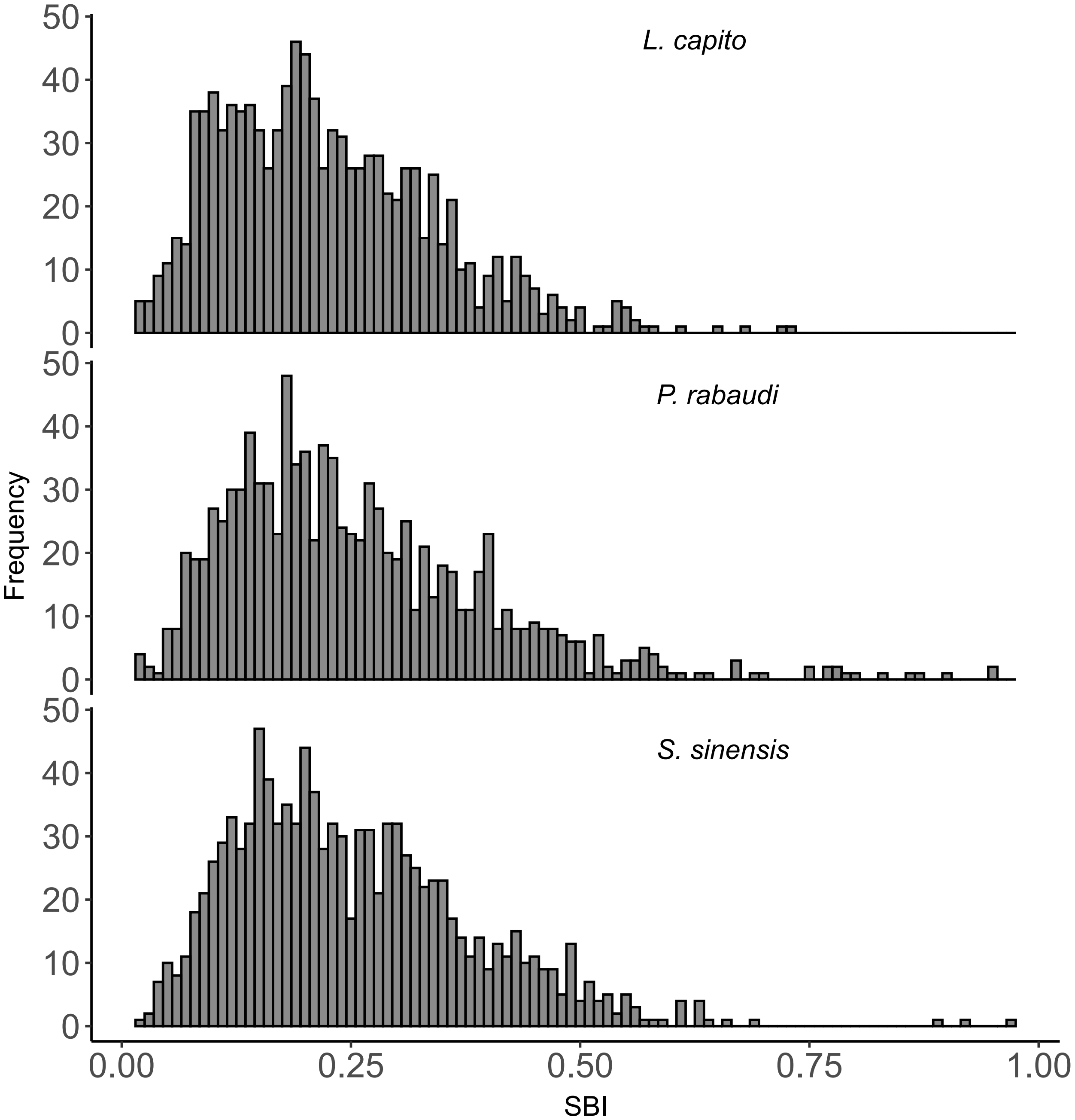

Supplementary Fig. 11. The distribution of subgenome-biased index (SBI) for TEs in the reference genome of three allotetraploids.

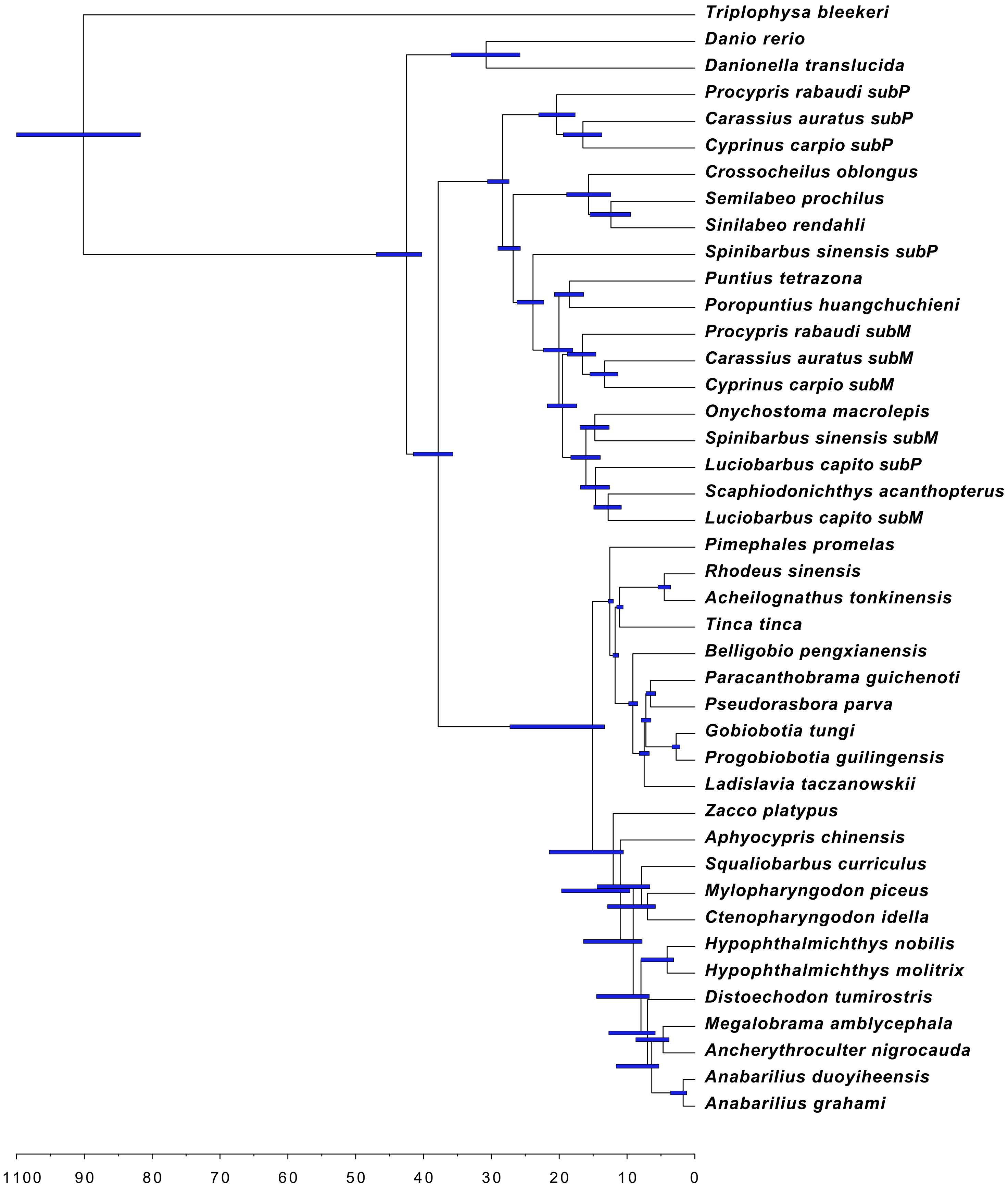

Supplementary Fig. 12. Divergence times within the Cyprinidae family and between subgenomes of five allotetraploids inferred by MCMCTree. Species tree constructed using IQ-TREE based on CDS of 300 one-to-one orthologues. *Triplophysa bleekeri* was used as the outgroup. See Supplementary Fig. 13.

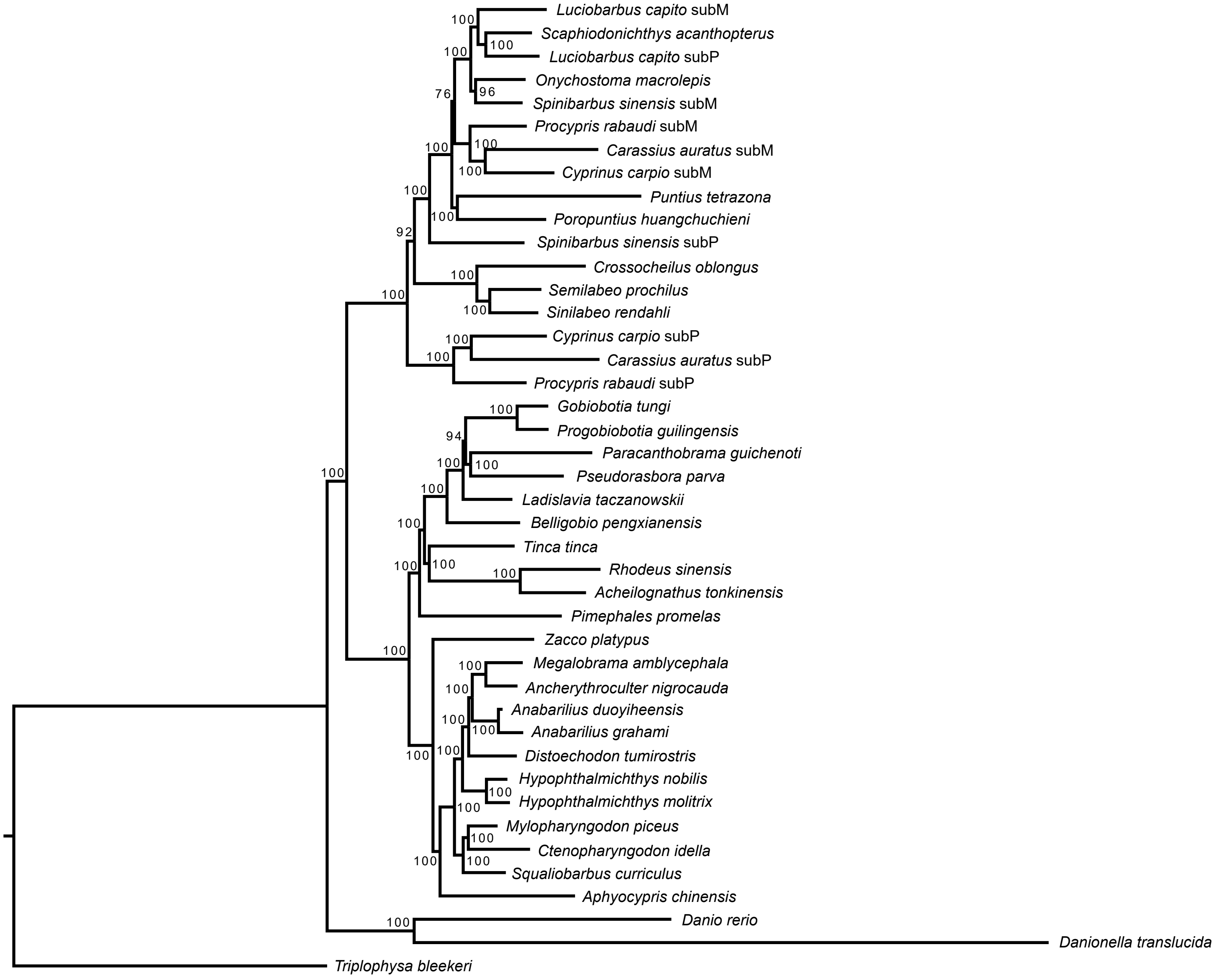

Supplementary Fig. 13. A Maximum Likelihood (ML) phylogenetic tree of the Cyprinidae family on the basis of CDS of 300 one-to-one orthologues using IQ-TREE. *T. bleekeri* was used as the outgroup. Bootstrap values supporting on each node are calculated with 1000 replicates.

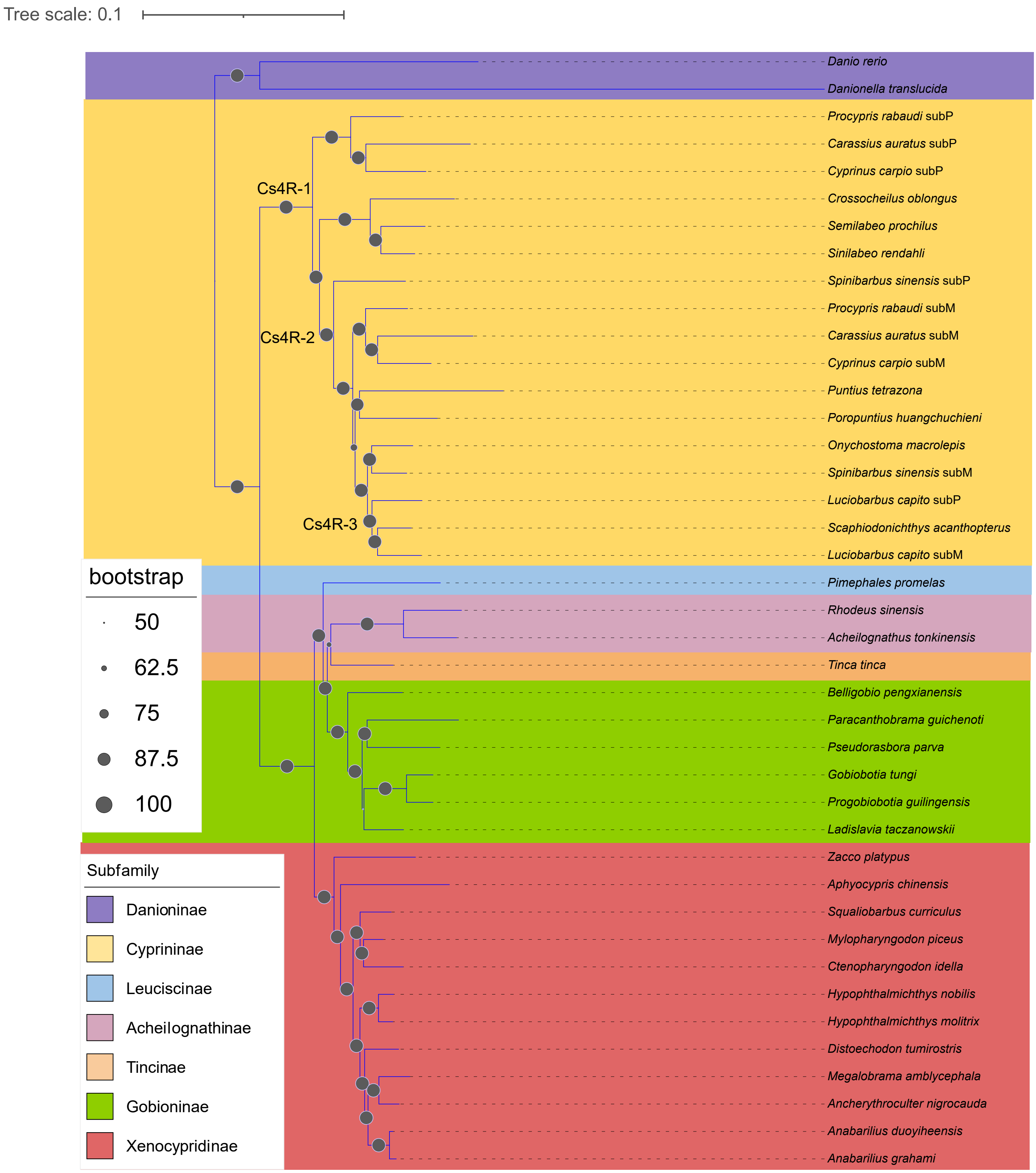

Supplementary Fig. 14. A Maximum Likelihood (ML) phylogenetic tree of the Cyprinidae family on the basis of CDS of 310 one-to-one orthologues using RAxML. Zebrafish and *Danionella translucida* were used as the outgroup.

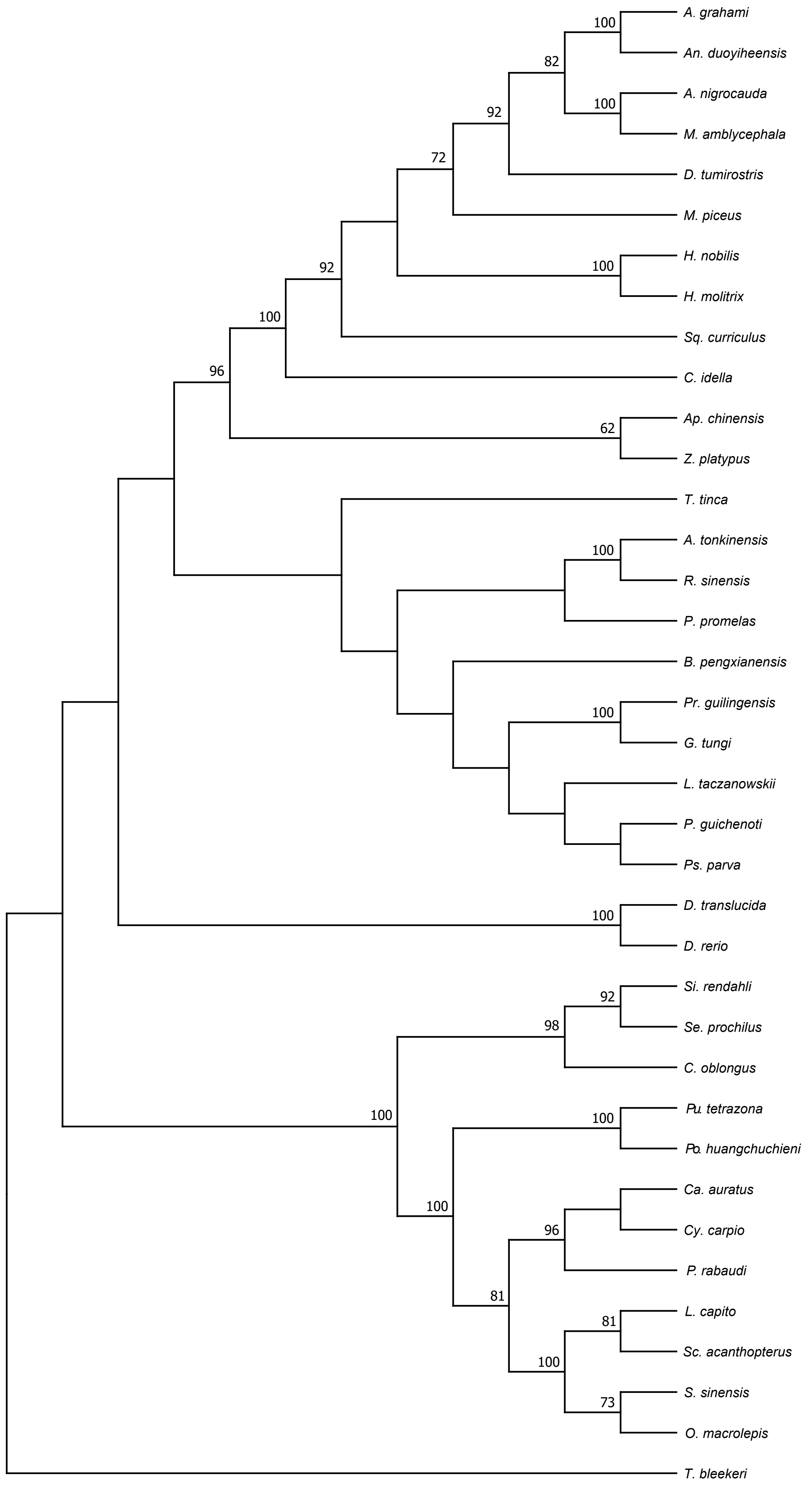

Supplementary Fig. 15. A phylogeny of the Cyprinidae family based on mitochondria genomes. We used mitochondrial genomes of 37 species to infer this phylogeny by RAxML using *T. bleekeri* as the outgroup. Bootstrap values lower than 60 were not shown.

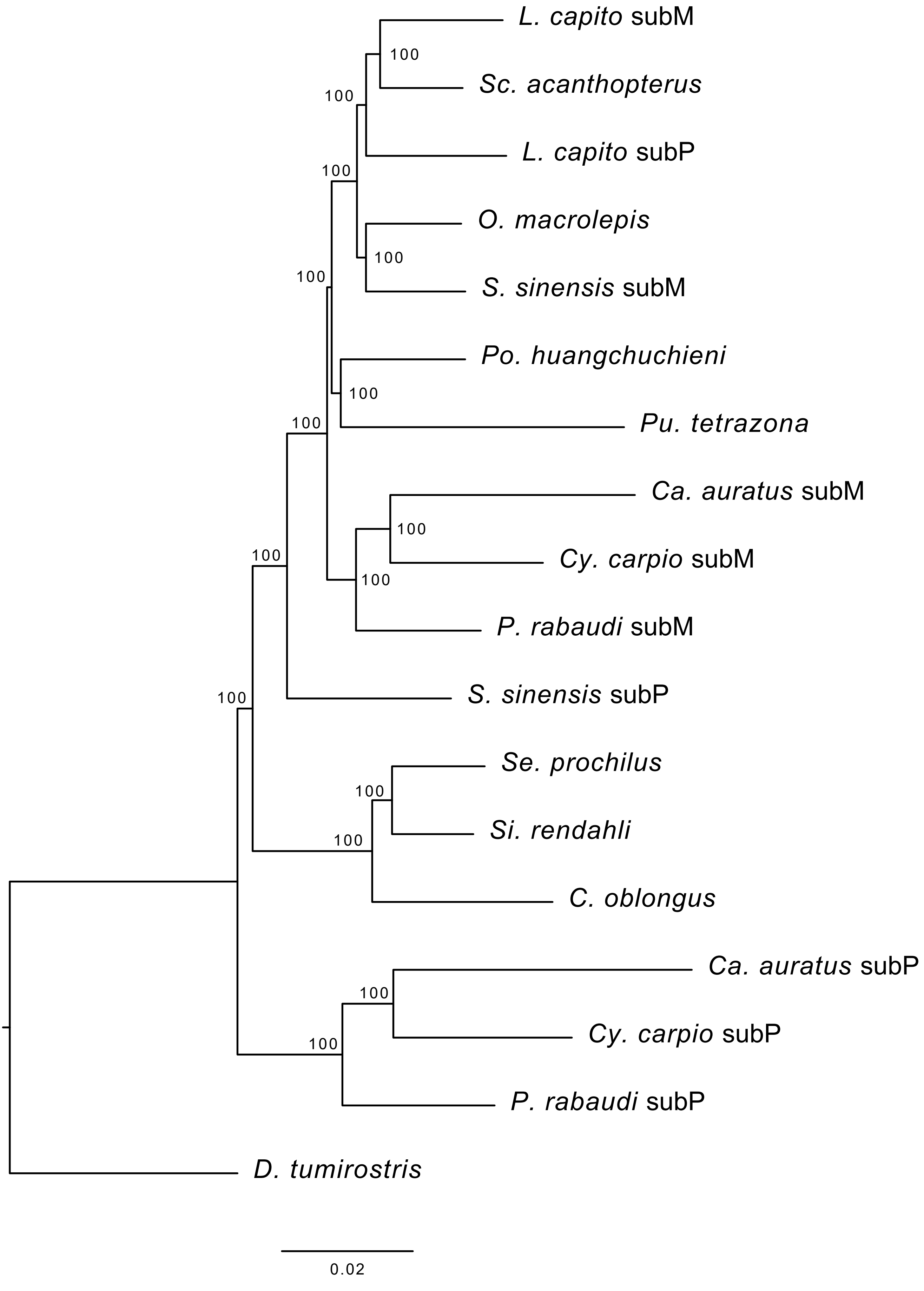

Supplementary Fig. 16. A ML tree made from the whole genome alignment (WGA). The WGA of 13 cyprinid fishes was used for building the ML phylogenetic tree by RAxML. Numbers on the nodes represent the support values from 200 bootstrap tests. We used *Distoechodon tumirostris* as the outgroup.

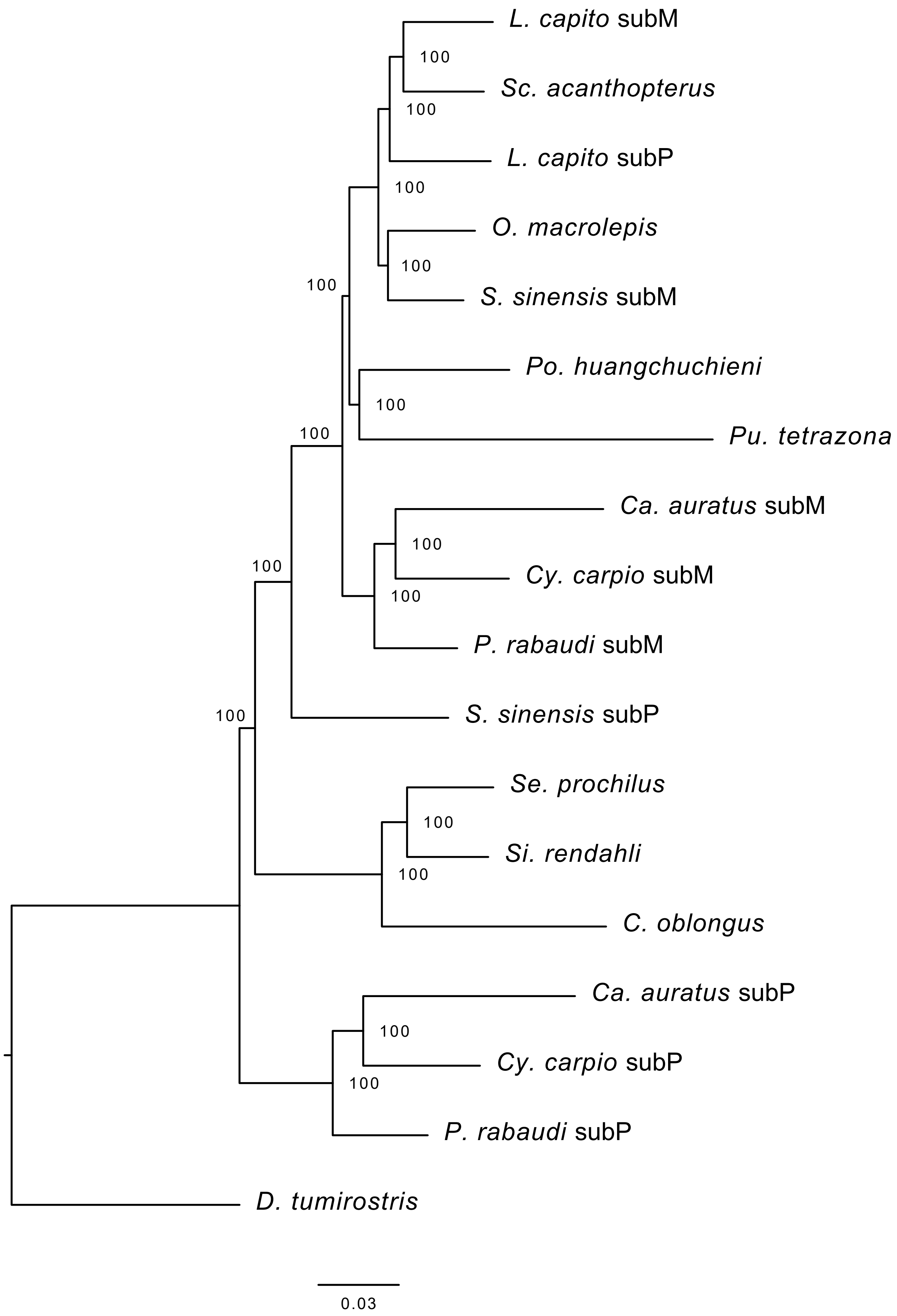

Supplementary Fig. 17. A ML phylogenetic tree generated from 4d sites. 252,437 4d sites of 1,669 single-copy orthologs from 13 cyprinid fishes were identified and used for constructing a ML tree by RAxML. Numbers on the nodes represent the support values from 200 bootstrap tests. *D. tumirostris* was used as the outgroup.

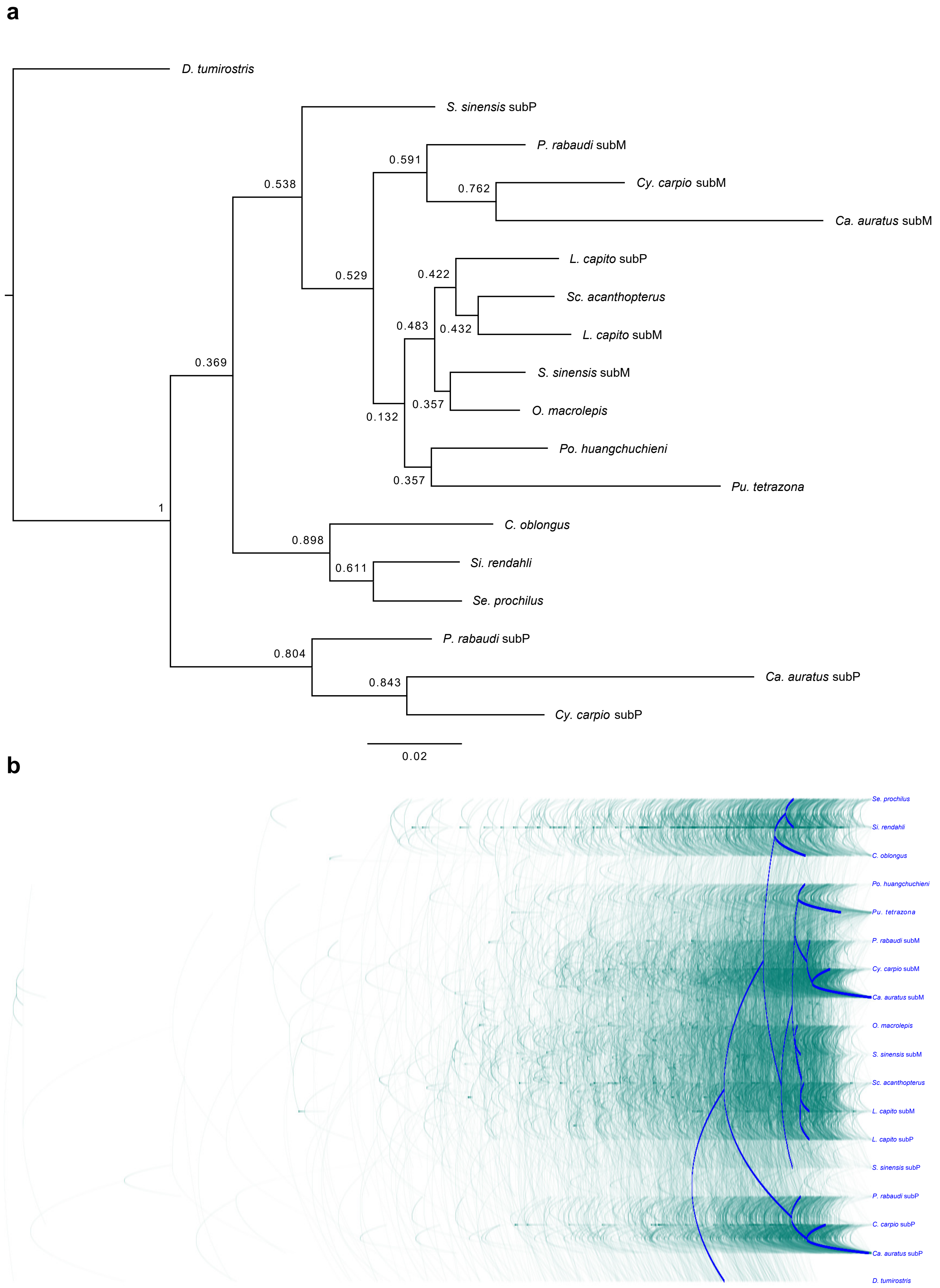

Supplementary Fig. 18. a Summary tree across 1665 genetrees, inferred by SumTrees. Values represent the degree of support for clades indicated as proportions (posterior probabilities). **b** DensiTree of 1665 genetrees, constructed using MUSCLE and RaxML-NG; green: concensus trees across multiple gene trees; blue: summary tree.

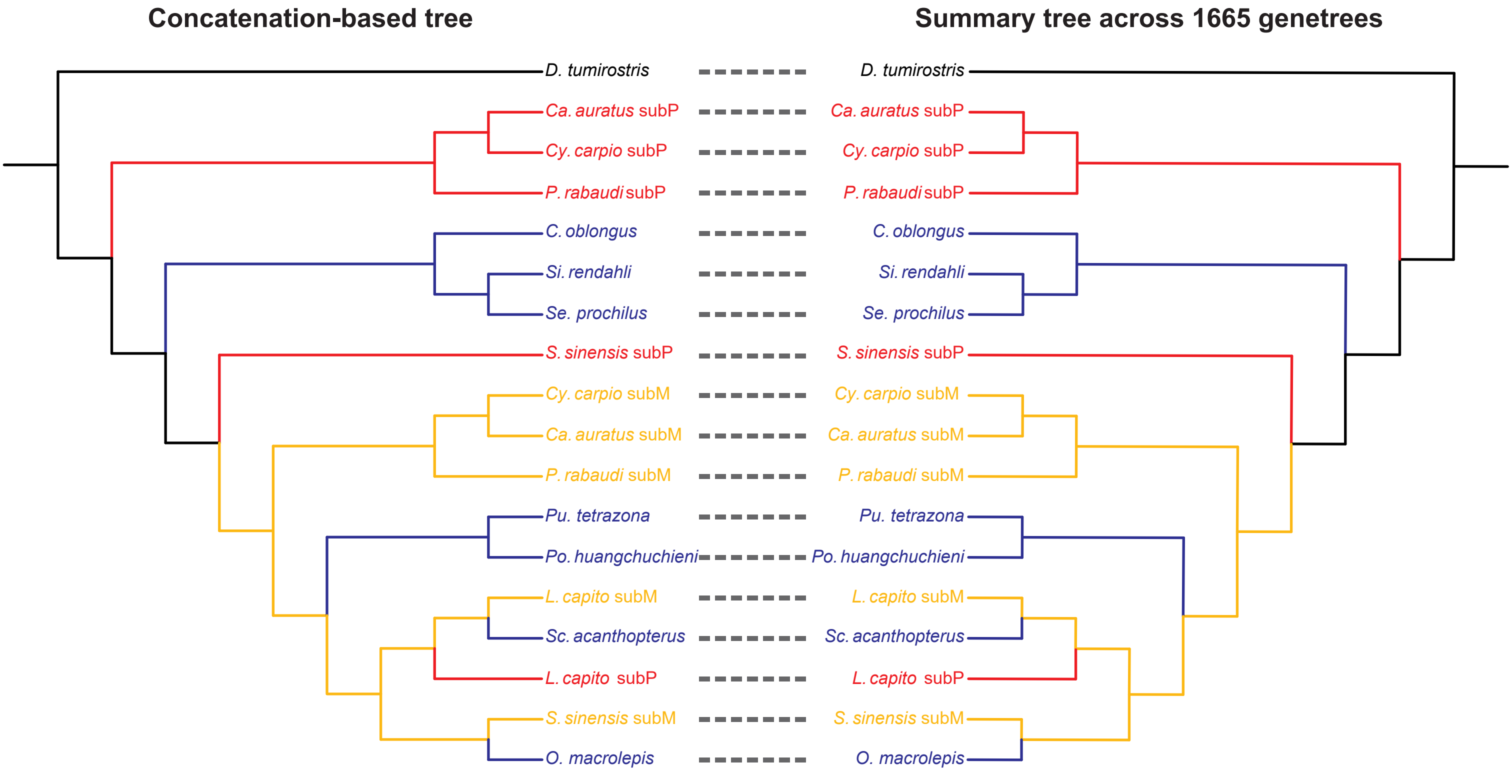

Supplementary Fig. 19. Congruences between concatenation-based tree (left) and summary tree across 1665 genetrees (right).

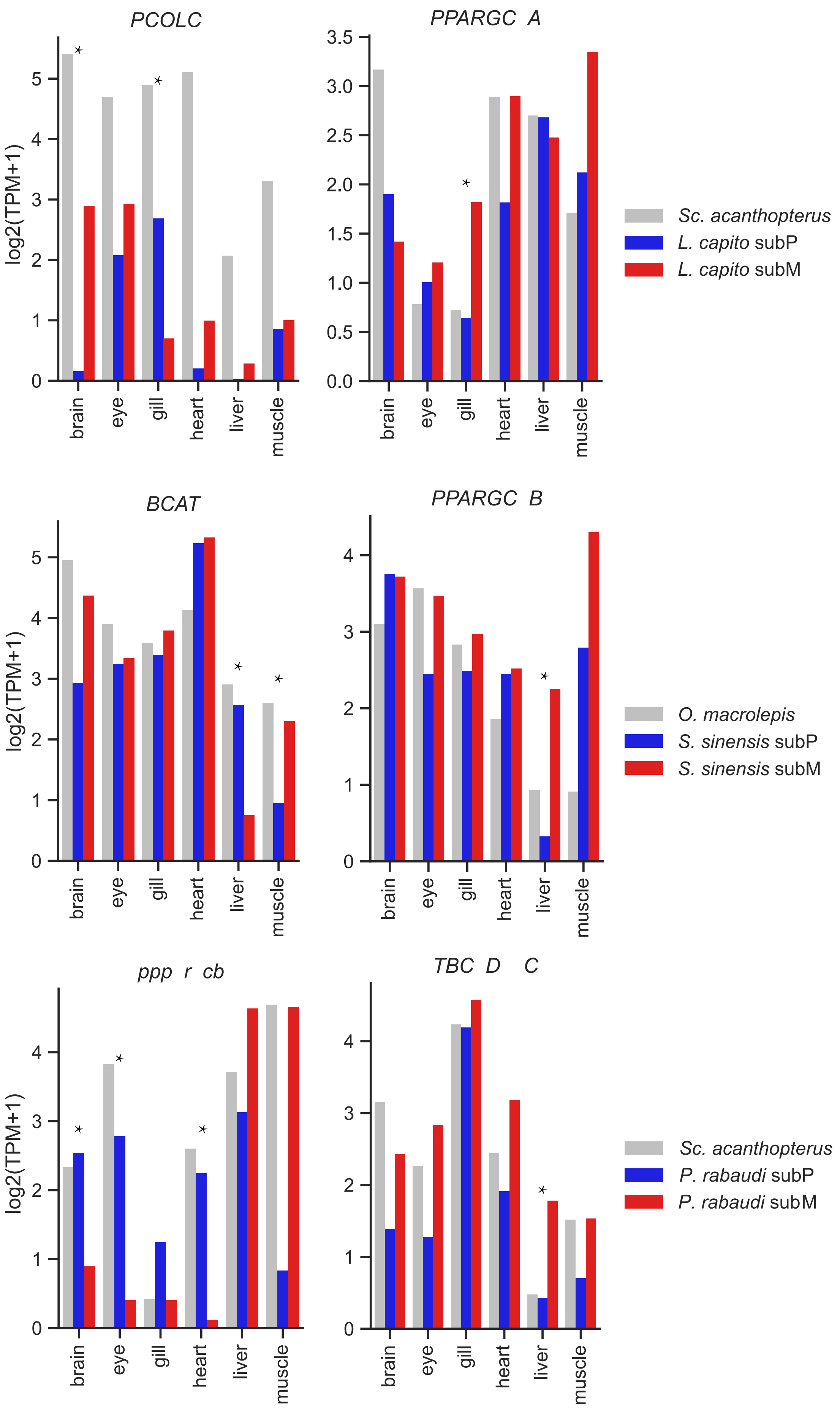

Supplementary Fig. 20. Examples of expression of sub-F (left) and neo-F (right) genes. Gray bar, *Sc. acanthopterus* or *O. macrolepis*; blue bar, subP of each allotetraploid; red bar, subM of each allotetraploid. Tissues related with sub-F or neo-F were showed using asterisks.

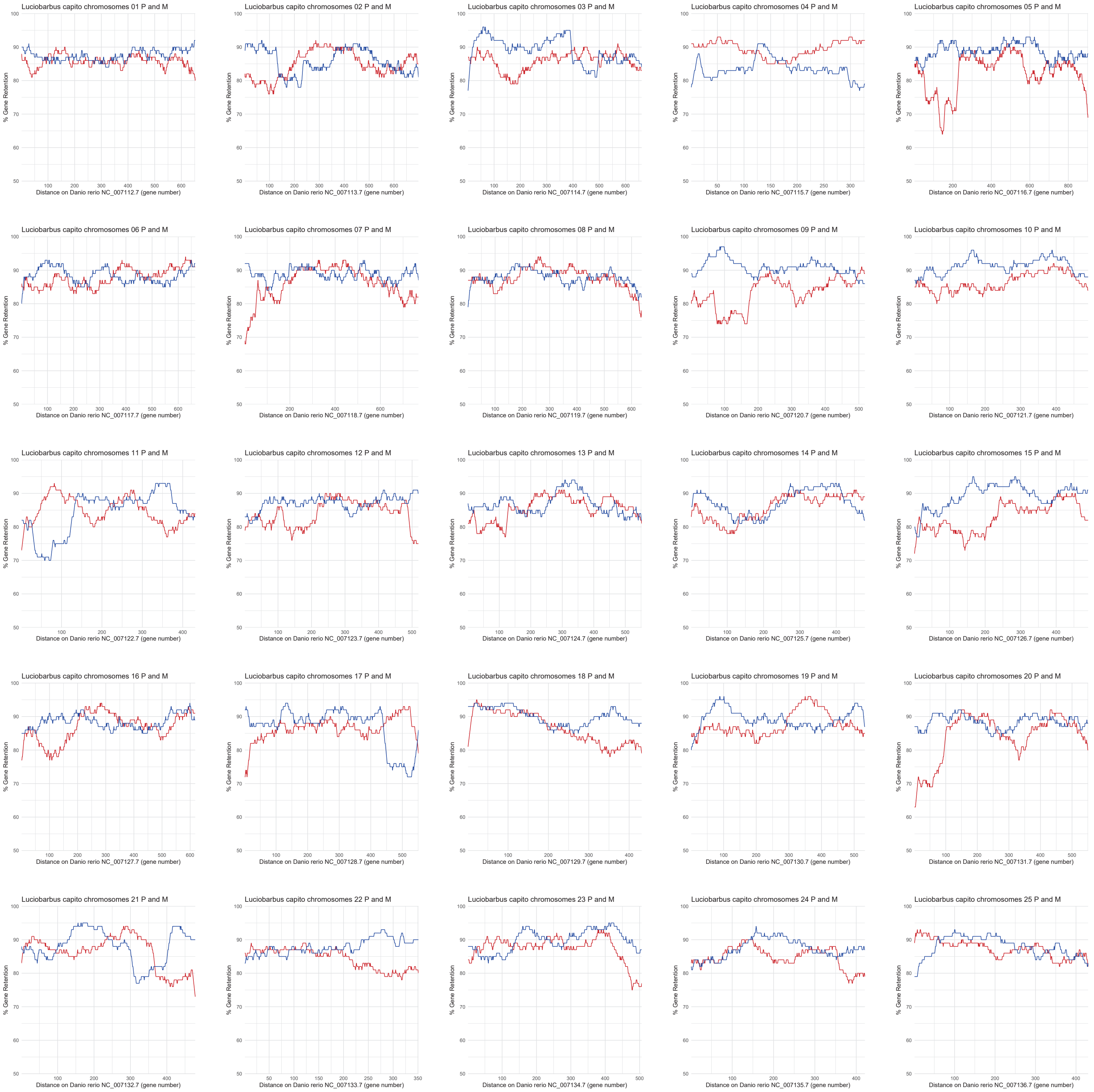

Supplementary Fig. 21. Subgenome fractionation of *L. capito* chromosomes relative to the diploid *Danio rerio*. Gene retention in *L. capito* subP (red) and subM (blue) was calculated in 100 gene sliding windows for each chromosome of the *D. rerio* reference.

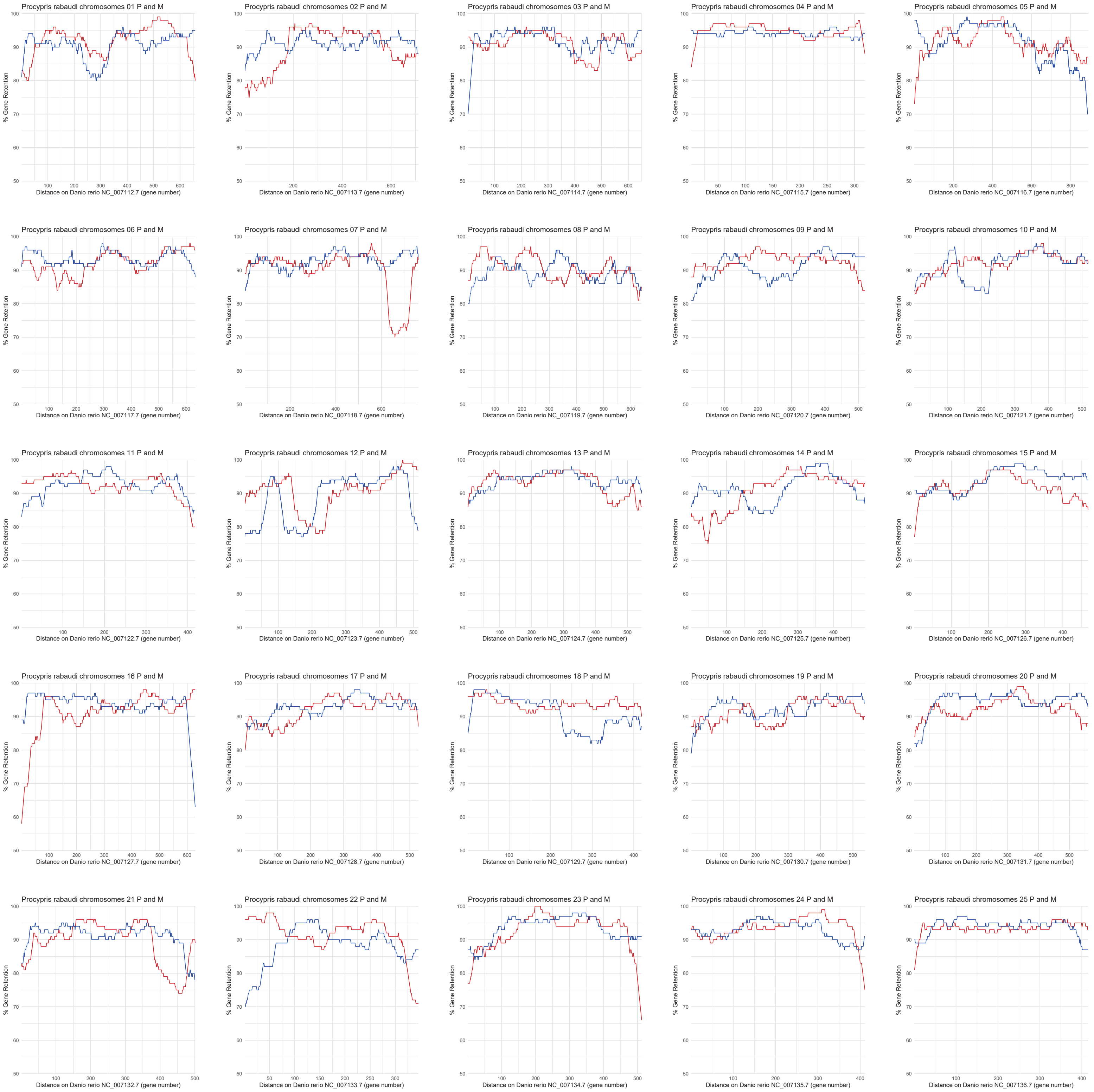

Supplementary Fig. 22. Subgenome fractionation of *P. rabaudi* chromosomes relative to the diploid *D. rerio*. Gene retention in *P. rabaudi* subP (red) and subM (blue) was calculated in 100 gene sliding windows for each chromosome of the *D. rerio* reference.

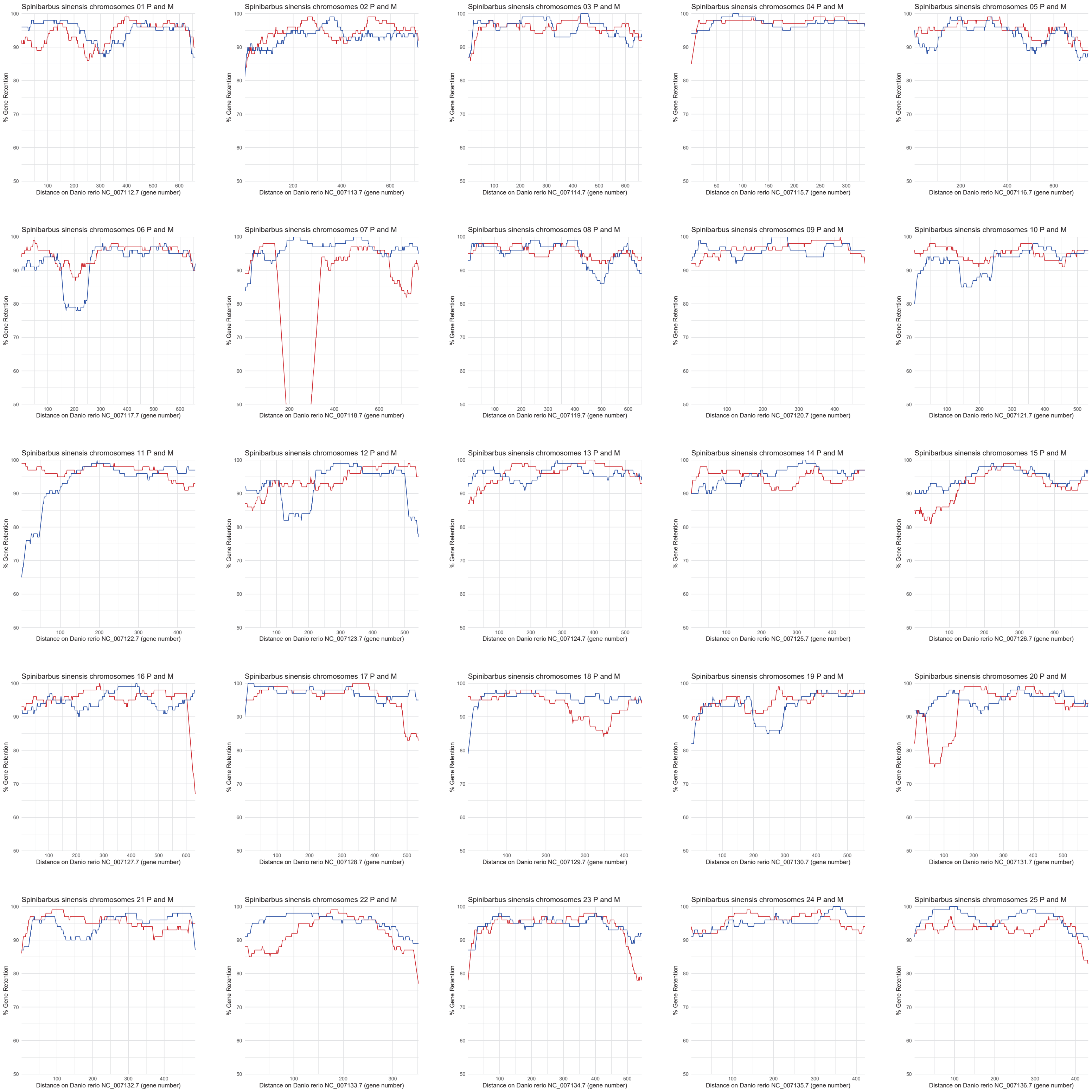

Supplementary Fig. 23. Subgenome fractionation of *S. sinensis* chromosomes relative to the diploid *D. rerio*. Gene retention in *S. sinensis* subP (red) and subM (blue) was calculated in 100 gene sliding windows for each chromosome of the *D. rerio* reference.

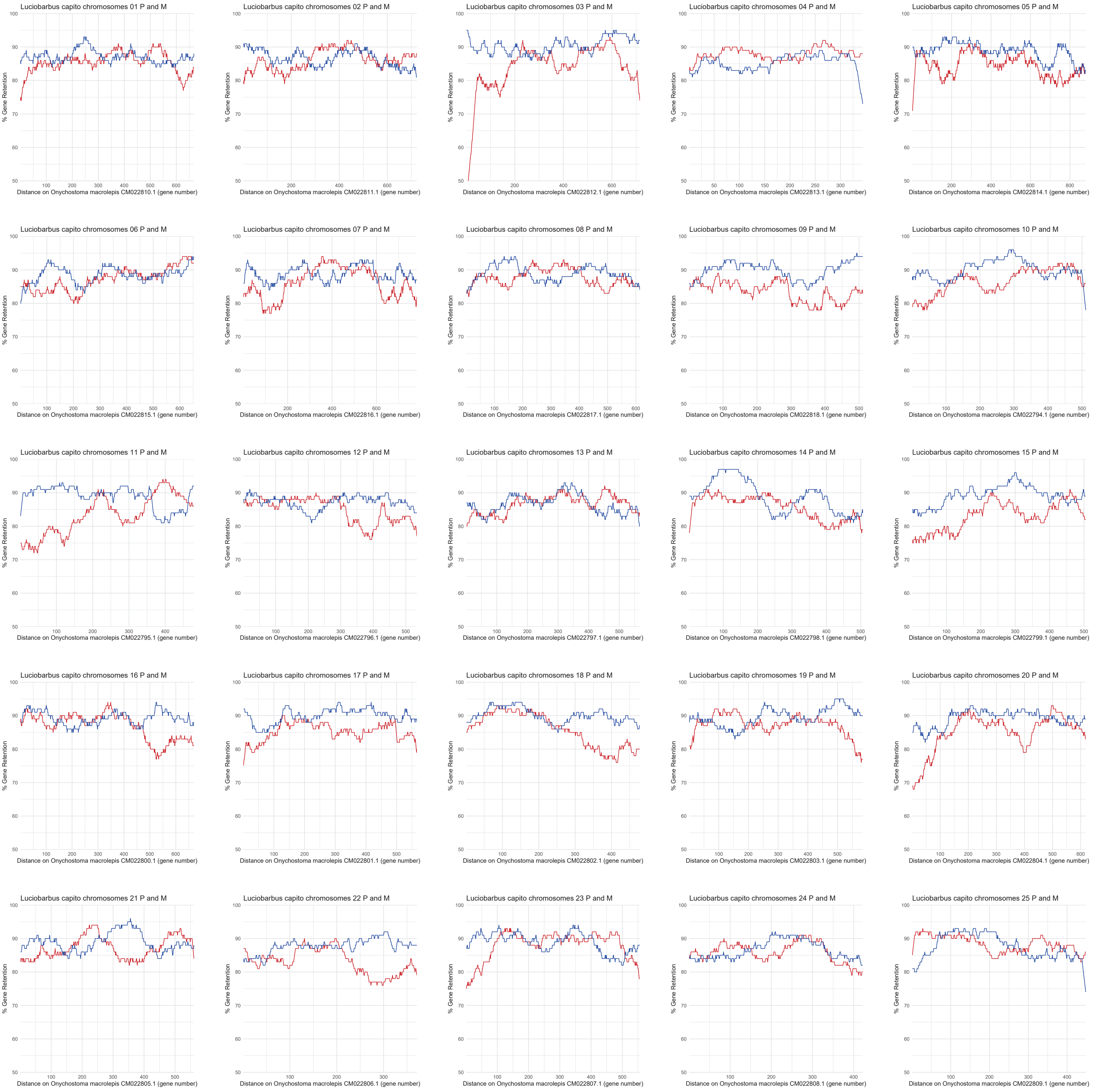

Supplementary Fig. 24. Subgenome fractionation of *L. capito* chromosomes relative to the diploid *O. macrolepis*. Gene retention in *L. capito* subP (red) and subM (blue) was calculated in 100 gene sliding windows for each chromosome of the *O. macrolepis* reference.

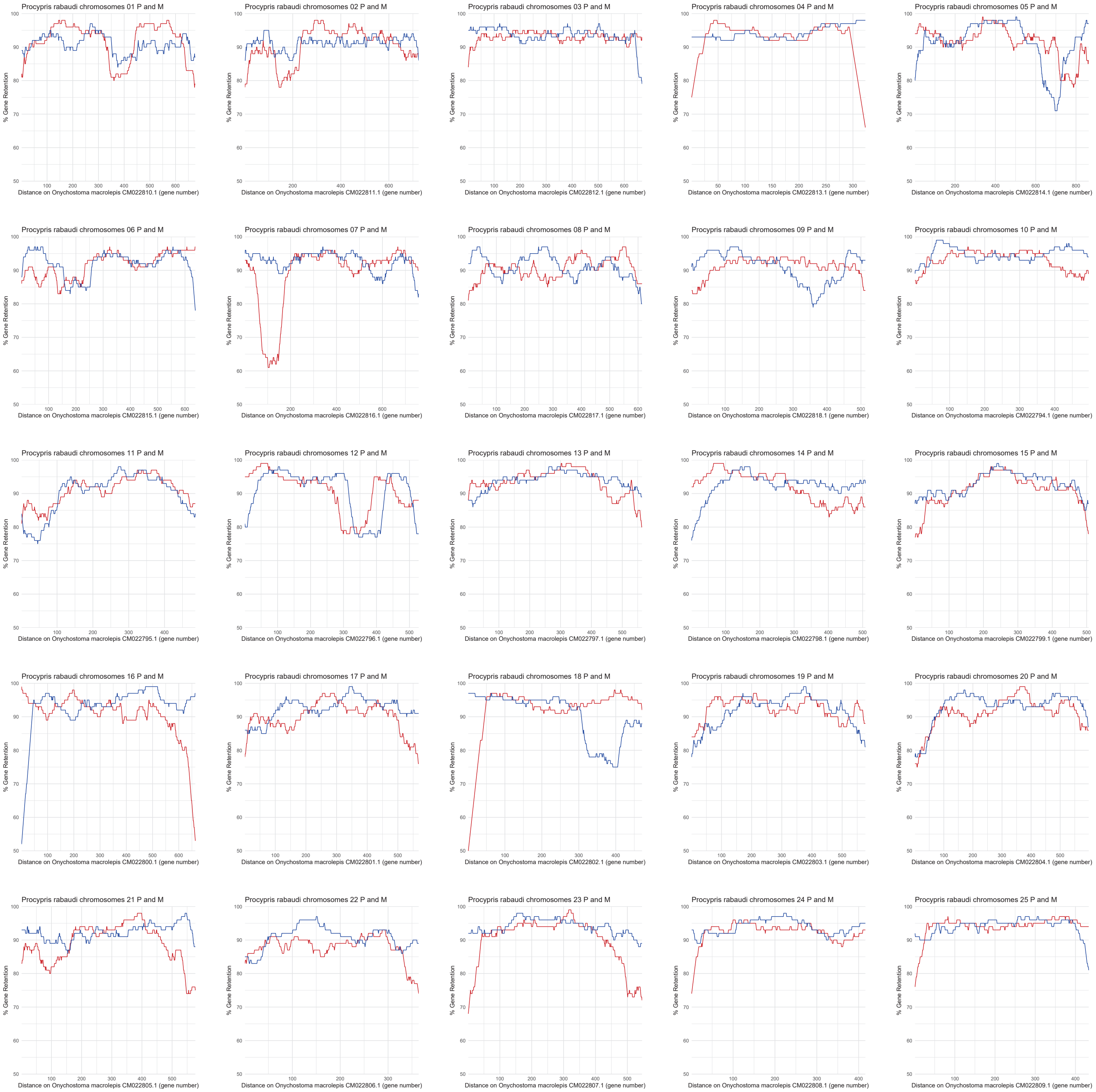

Supplementary Fig. 25. Subgenome fractionation of *P. rabaudi* chromosomes relative to the diploid *O. macrolepis*. Gene retention in *P. rabaudi* subP (red) and subM (blue) was calculated in 100 gene sliding windows for each chromosome of the *O. macrolepis* reference.

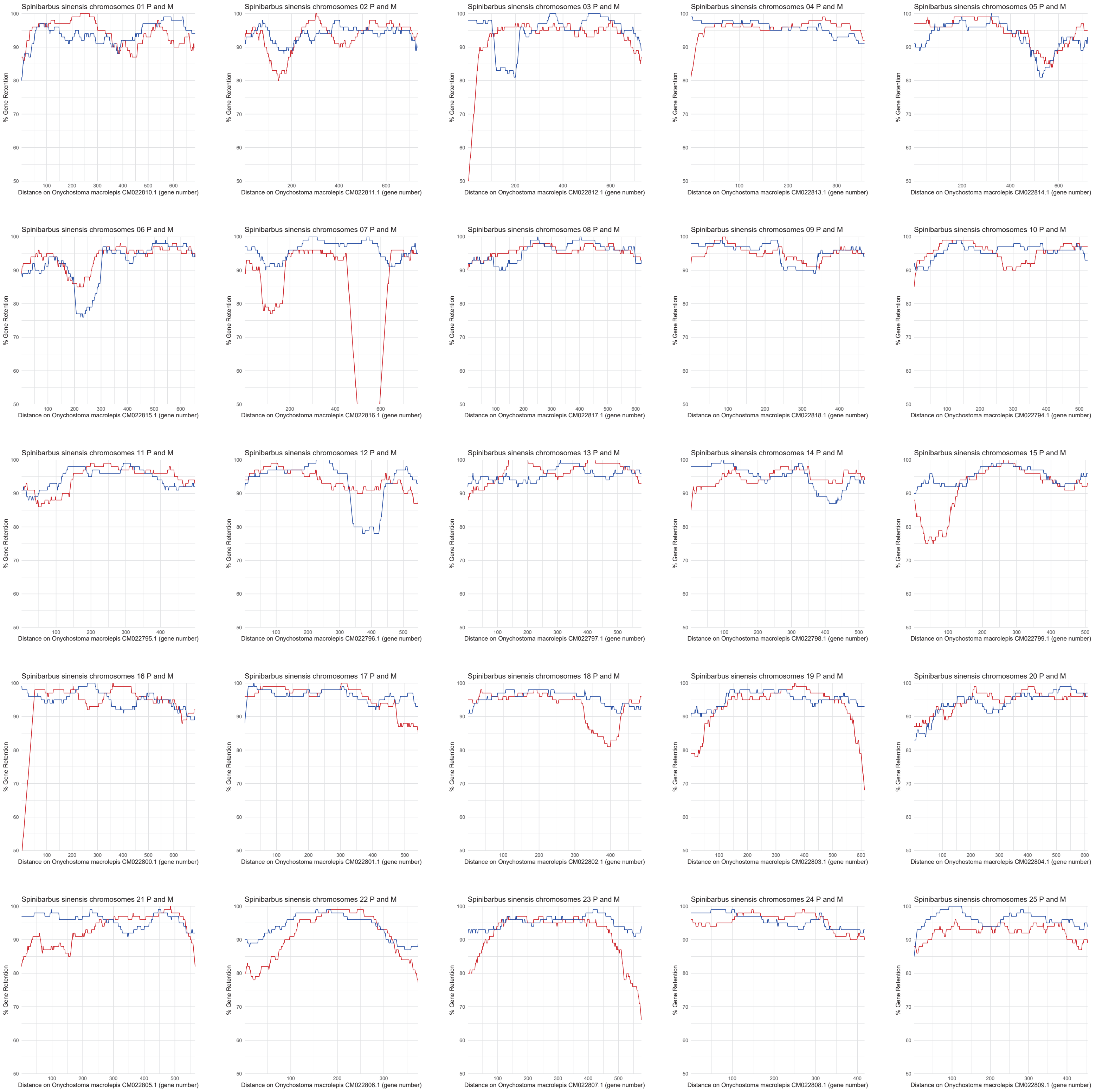

Supplementary Fig. 26. Subgenome fractionation of *S. sinensis* chromosomes relative to the diploid *O. macrolepis*. Gene retention in *S. sinensis* subP (red) and subM (blue) was calculated in 100 gene sliding windows for each chromosome of the *O. macrolepis* reference.

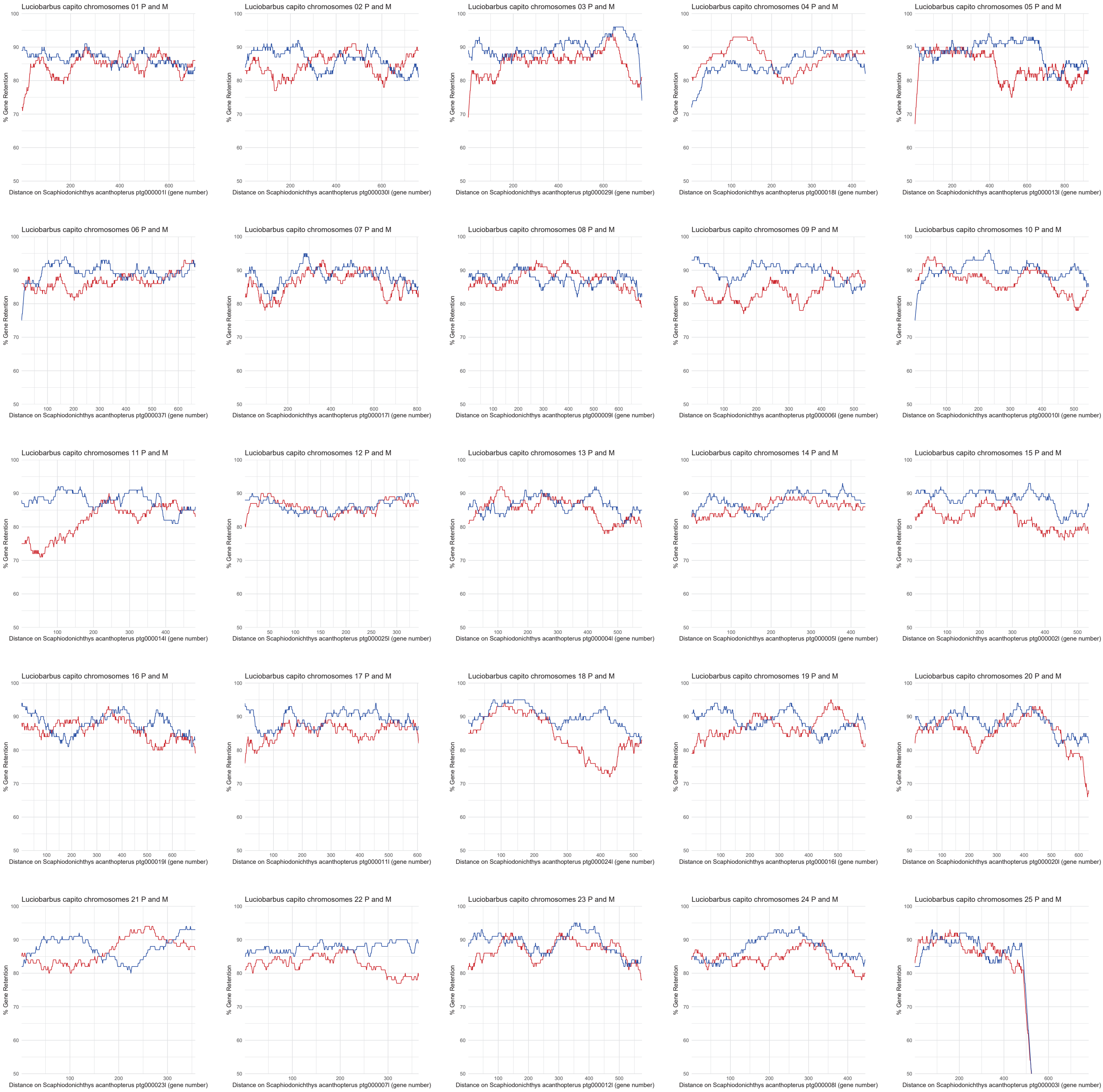

Supplementary Fig. 27. Subgenome fractionation of *L. capito* chromosomes relative to the diploid *Sc. acanthopterus*. Gene retention in *L. capito* subP (red) and subM (blue) was calculated in 100 gene sliding windows for each chromosome of the *Sc. acanthopterus* reference.

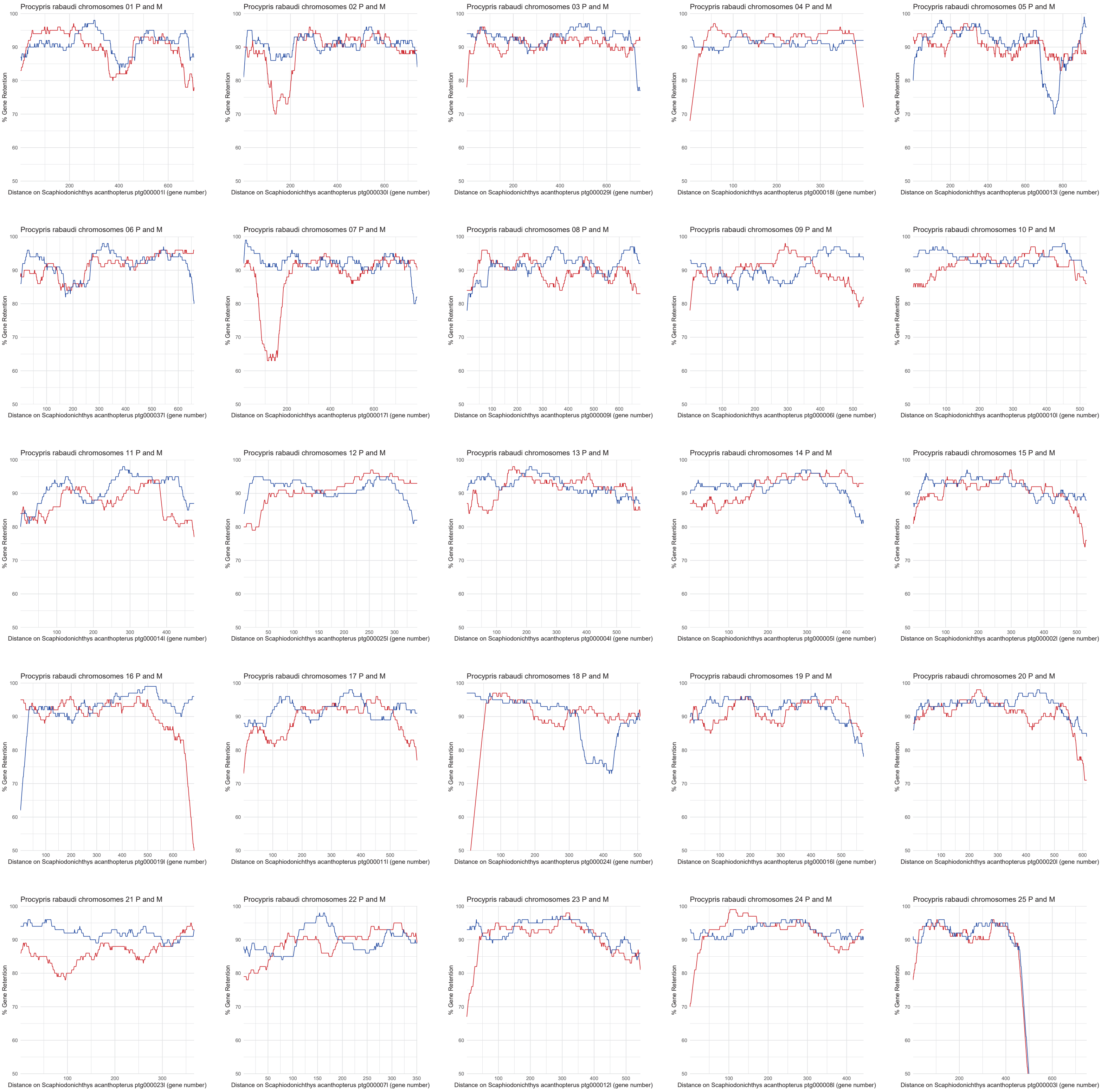

Supplementary Fig. 28. Subgenome fractionation of *P. rabaudi* chromosomes relative to the diploid *Sc. acanthopterus*. Gene retention in *P. rabaudi* subP (red) and subM (blue) was calculated in 100 gene sliding windows for each chromosome of the *Sc. acanthopterus* reference.

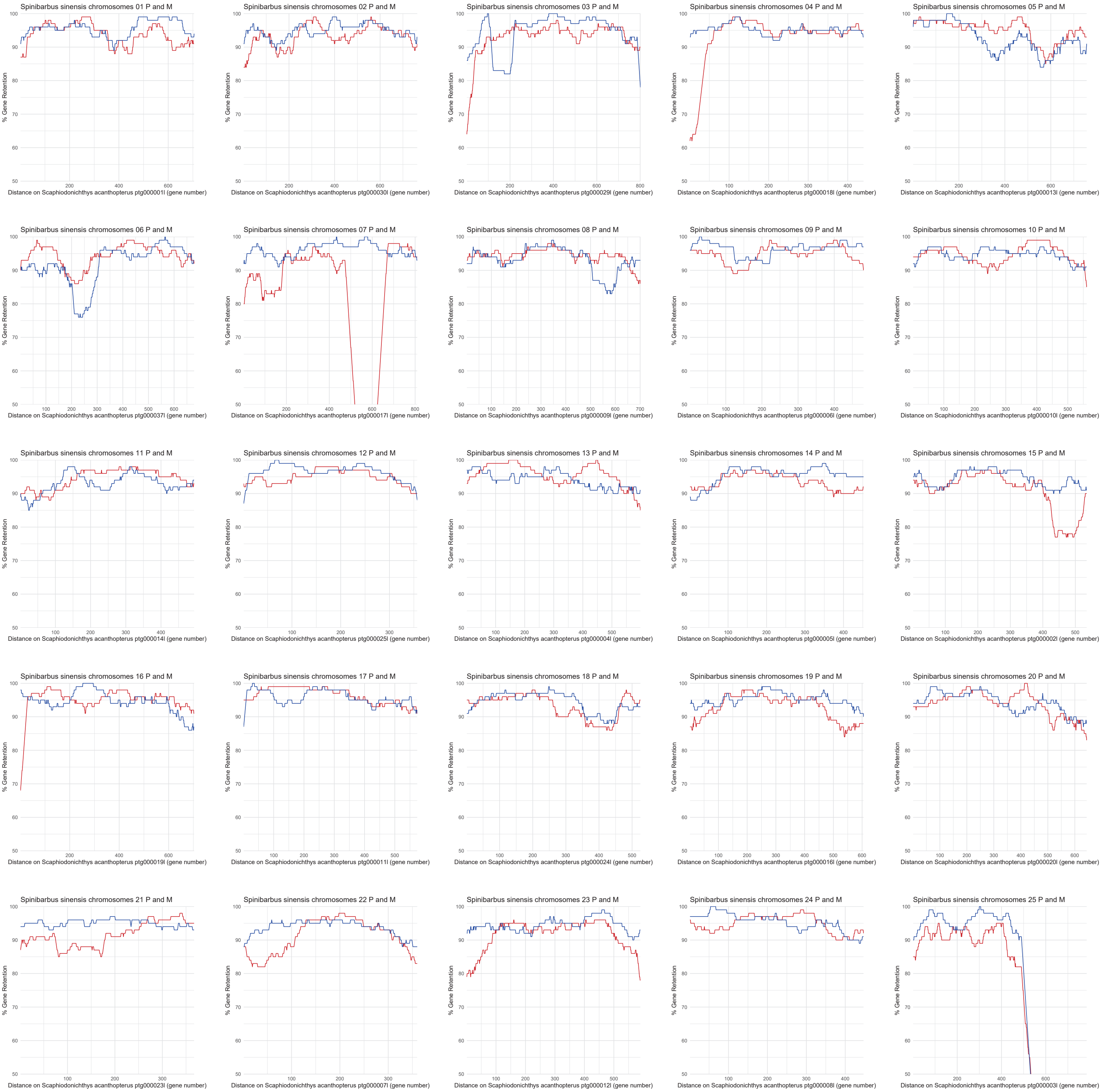

Supplementary Fig. 29. Subgenome fractionation of *S. sinensis* chromosomes relative to the diploid *Sc. acanthopterus*. Gene retention in *S. sinensis* subP (red) and subM (blue) was calculated in 100 gene sliding windows for each chromosome of the *Sc. acanthopterus* reference.

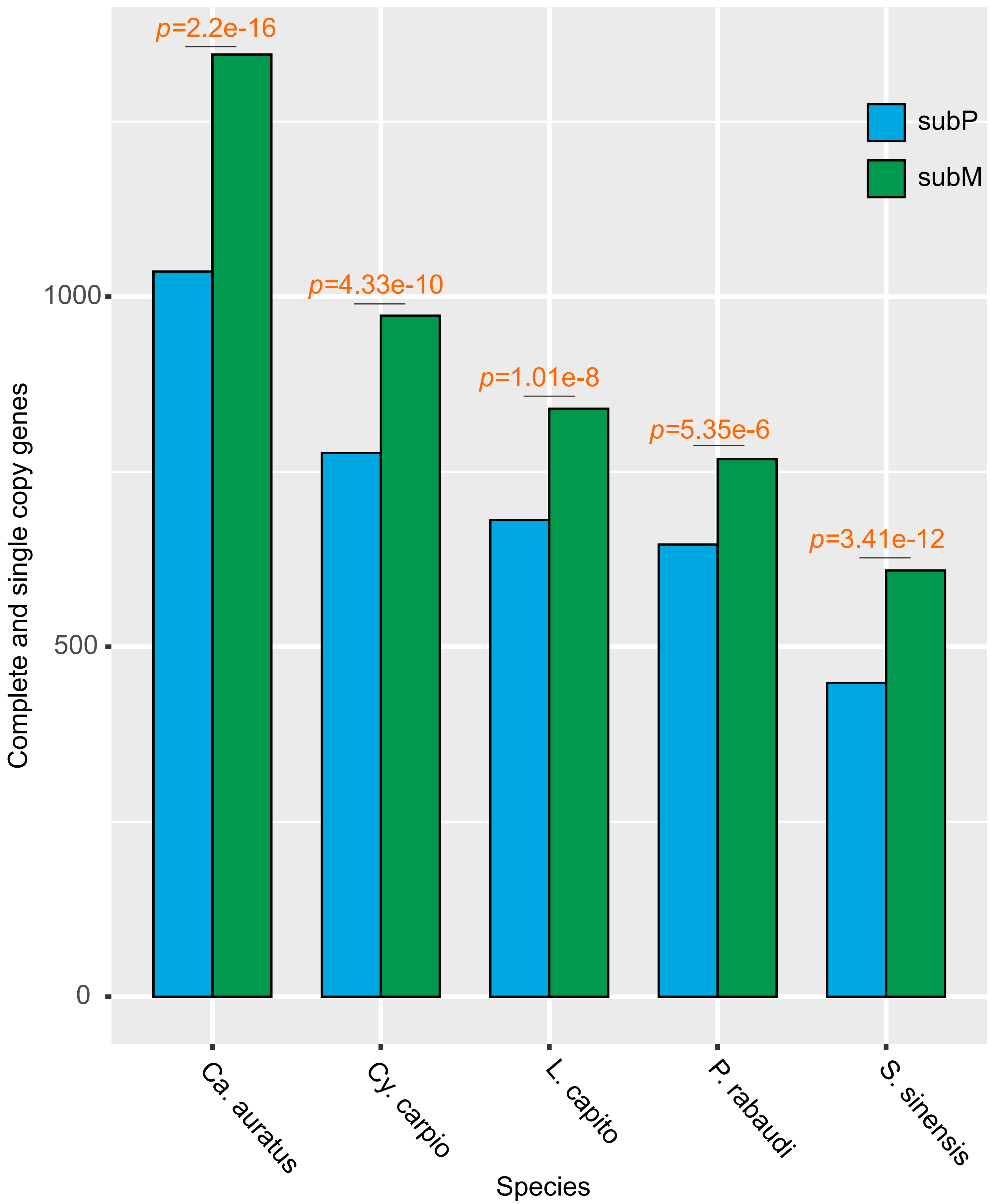

Supplementary Fig. 30. Biased distribution of complete and single copy genes generated by the BUSCO analysis in the subM of five allotetraploids (X^2 text; p-value≤5.35e-6).

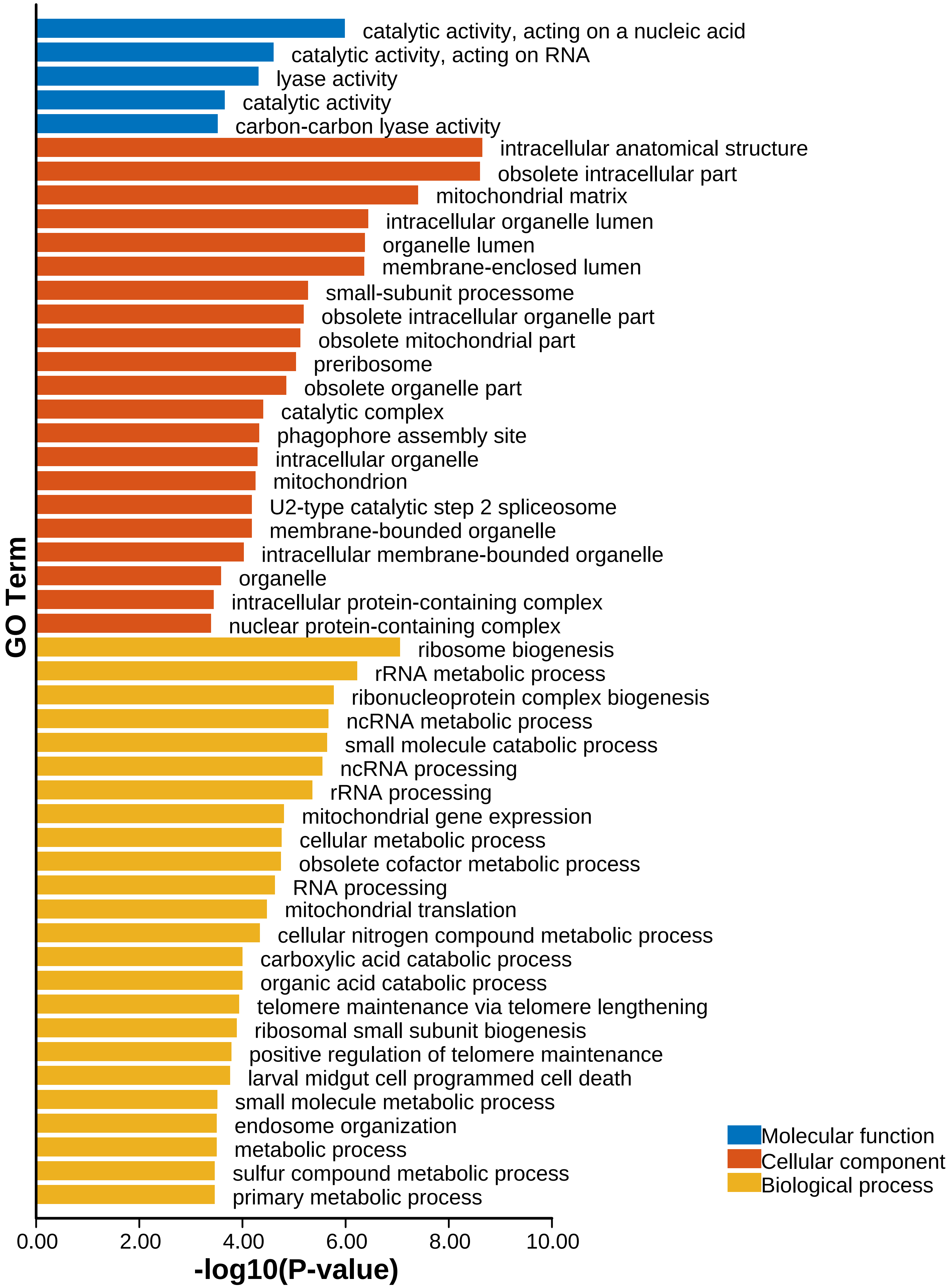

Supplementary Fig. 31. GO enrichment analysis of the complete and single copy BUSCO genes in the subP of *S. sinensis*.

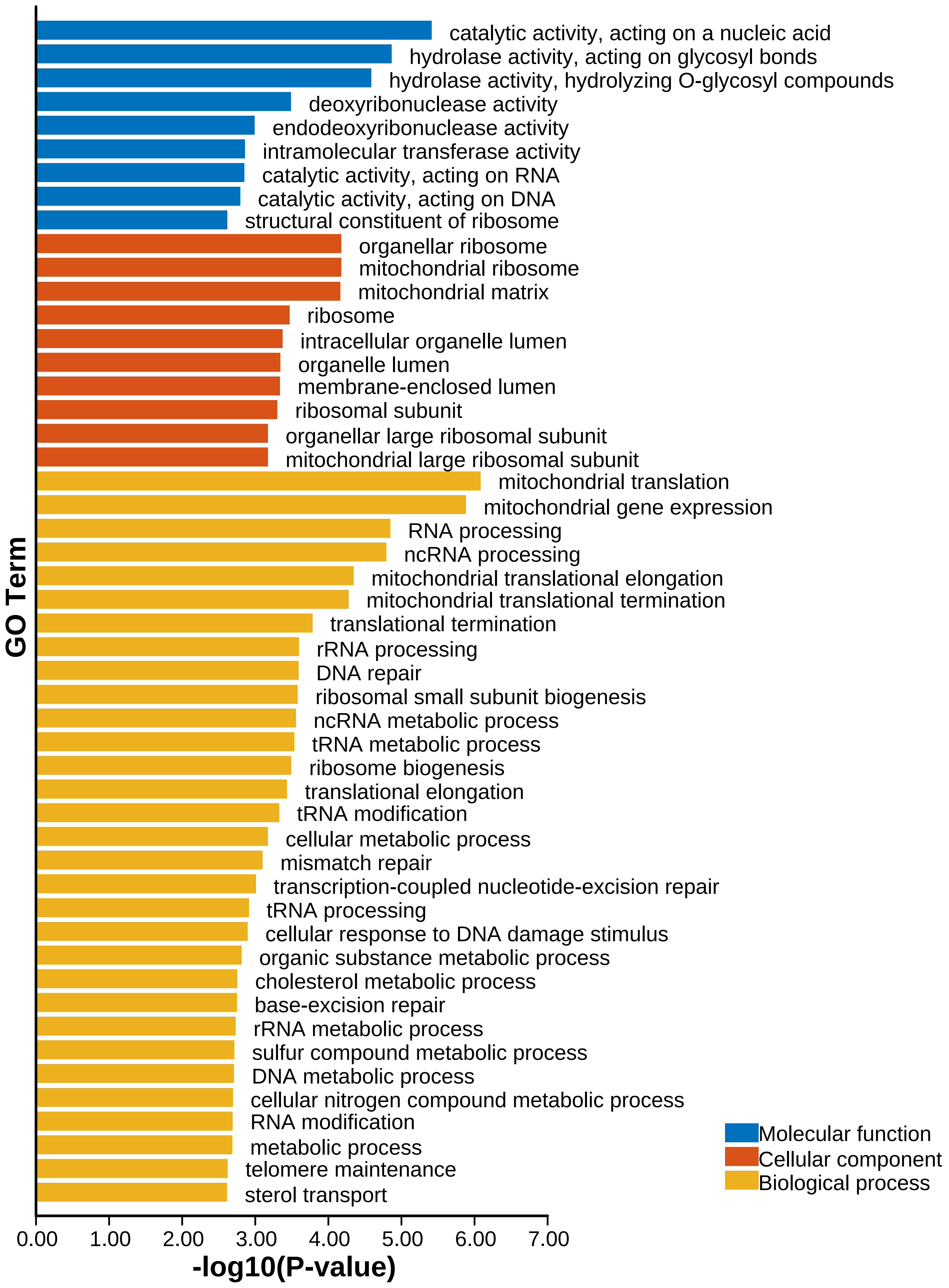

Supplementary Fig. 32. GO enrichment analysis of the complete and single copy BUSCO genes in the subM of *S. sinensis*.

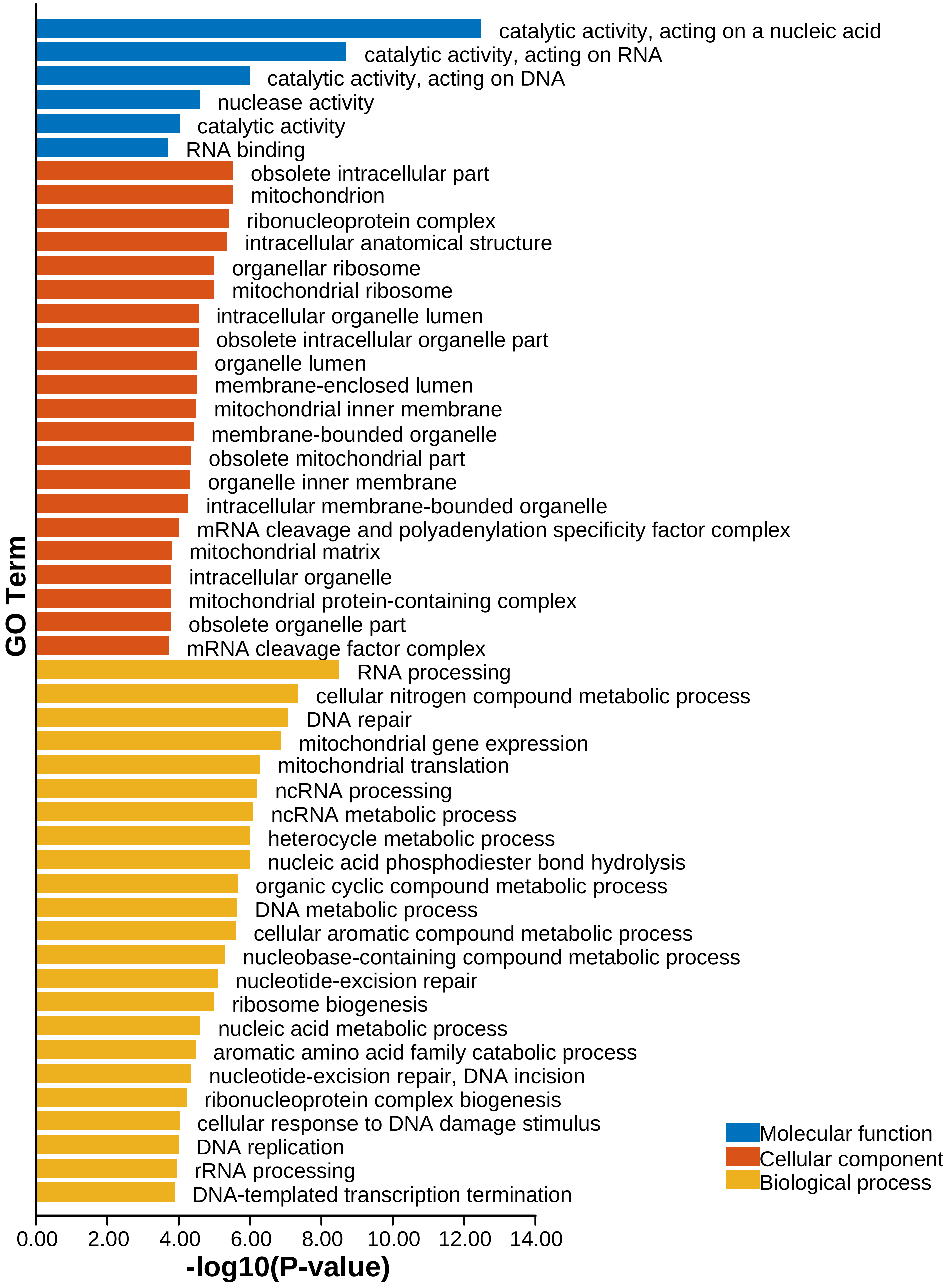

Supplementary Fig. 33. GO enrichment analysis of the complete and single copy BUSCO genes in the subP of *P. rabaudi*.

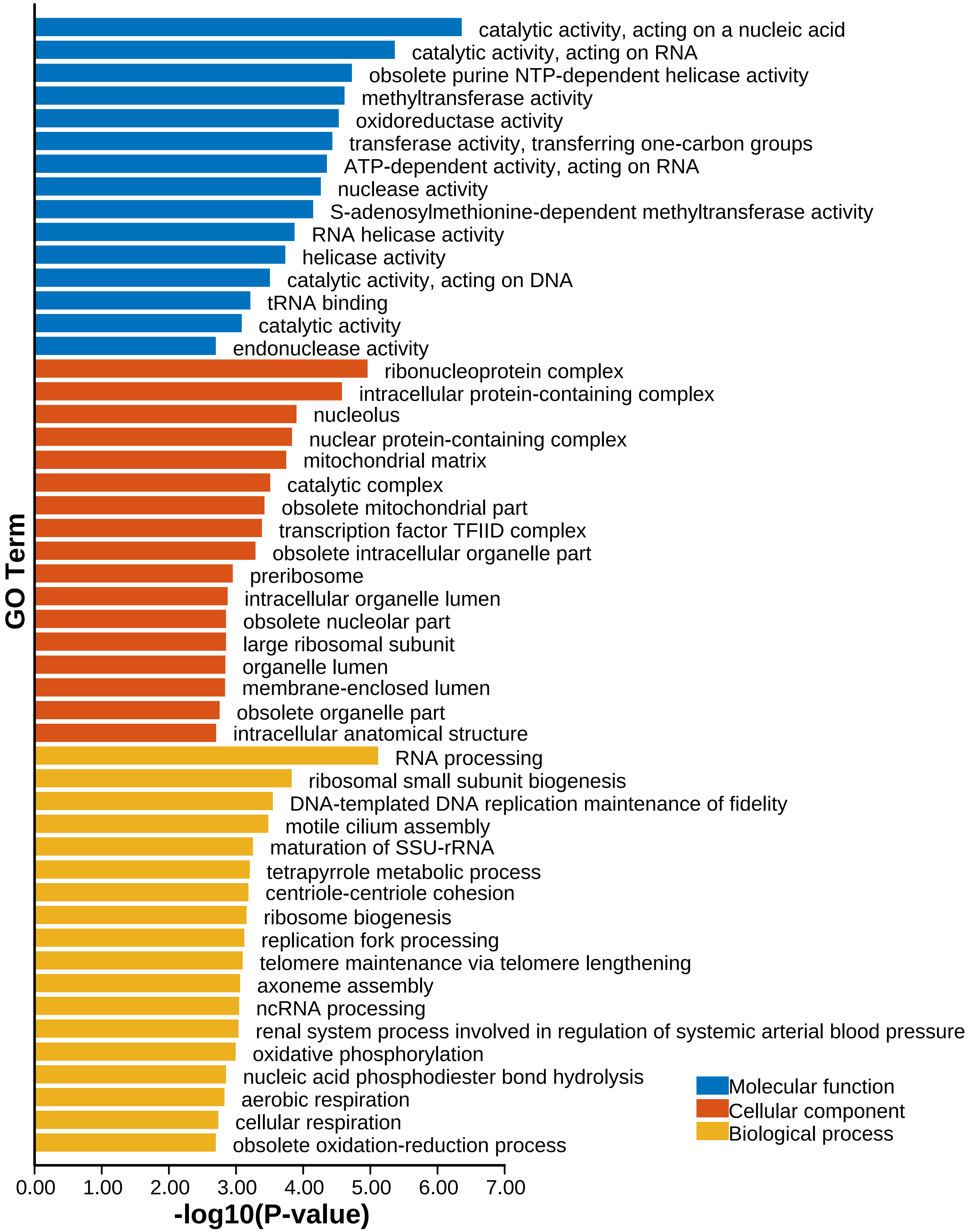

Supplementary Fig. 34. GO enrichment analysis of the complete and single copy BUSCO genes in the subM of *P. rabaudi*.

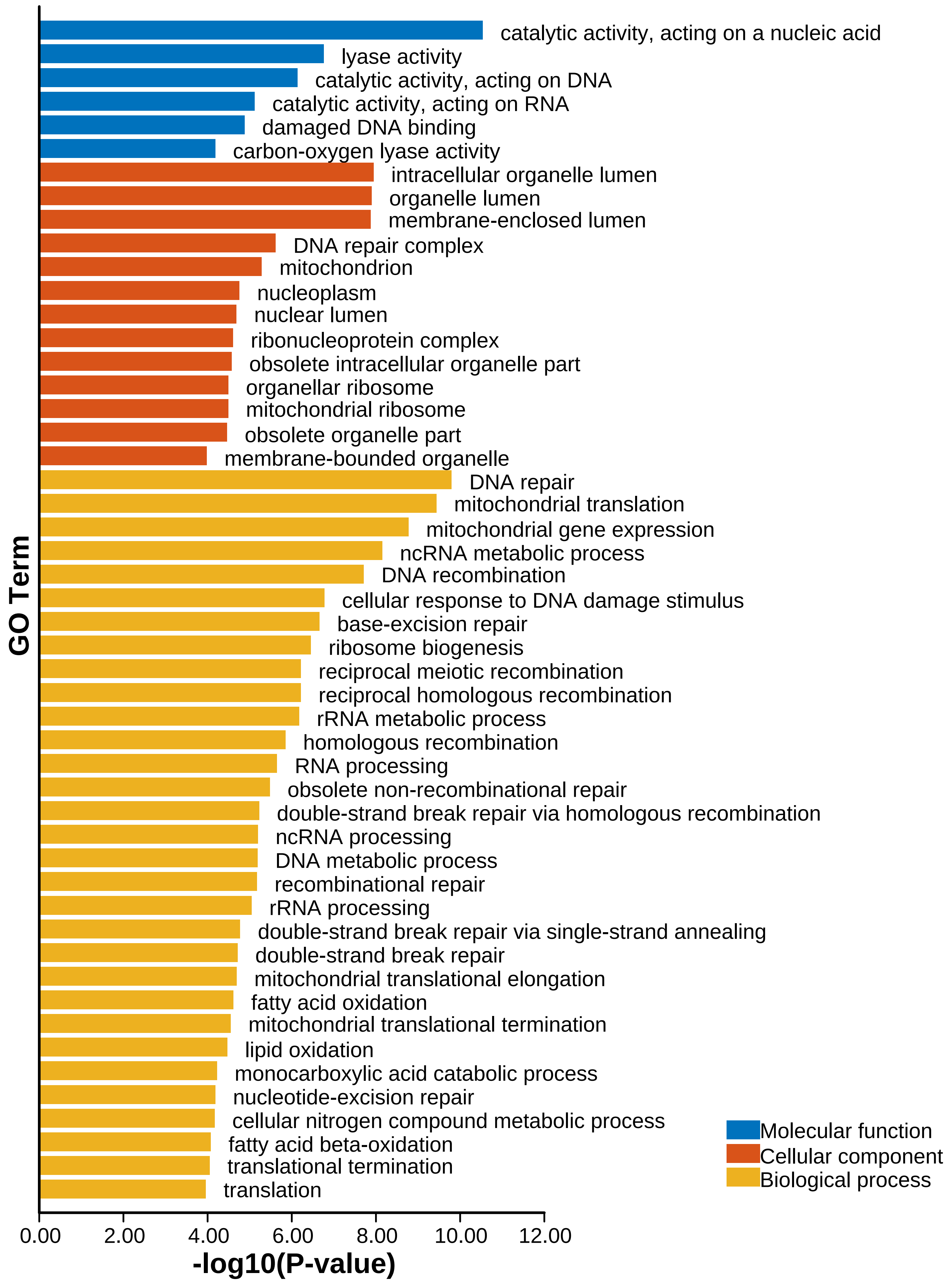

Supplementary Fig. 35. GO enrichment analysis of the complete and single copy BUSCO genes in the subP of *L. capito*.

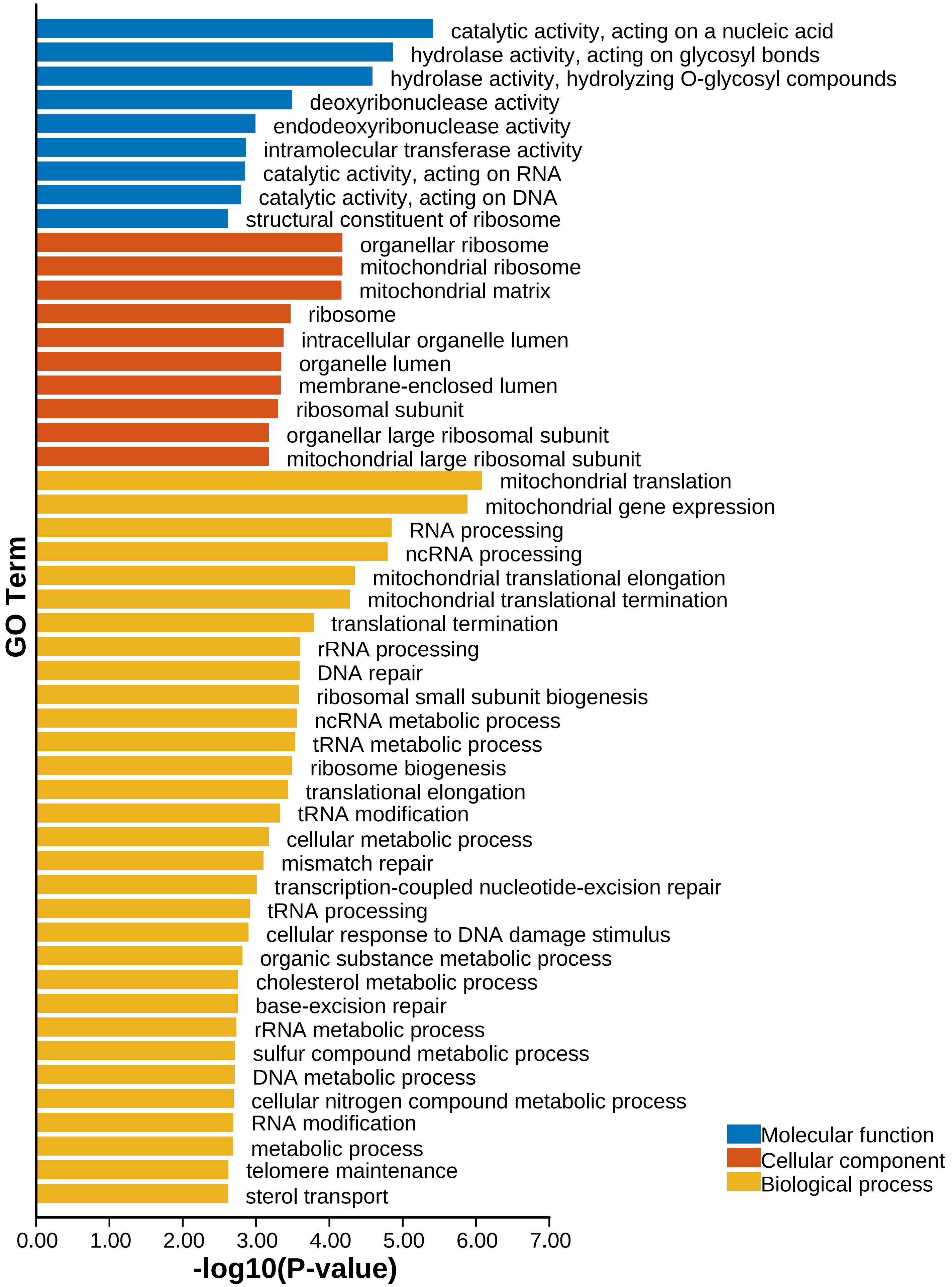

Supplementary Fig. 36. GO enrichment analysis of the complete and single copy BUSCO genes in the subM of *L. capito*.

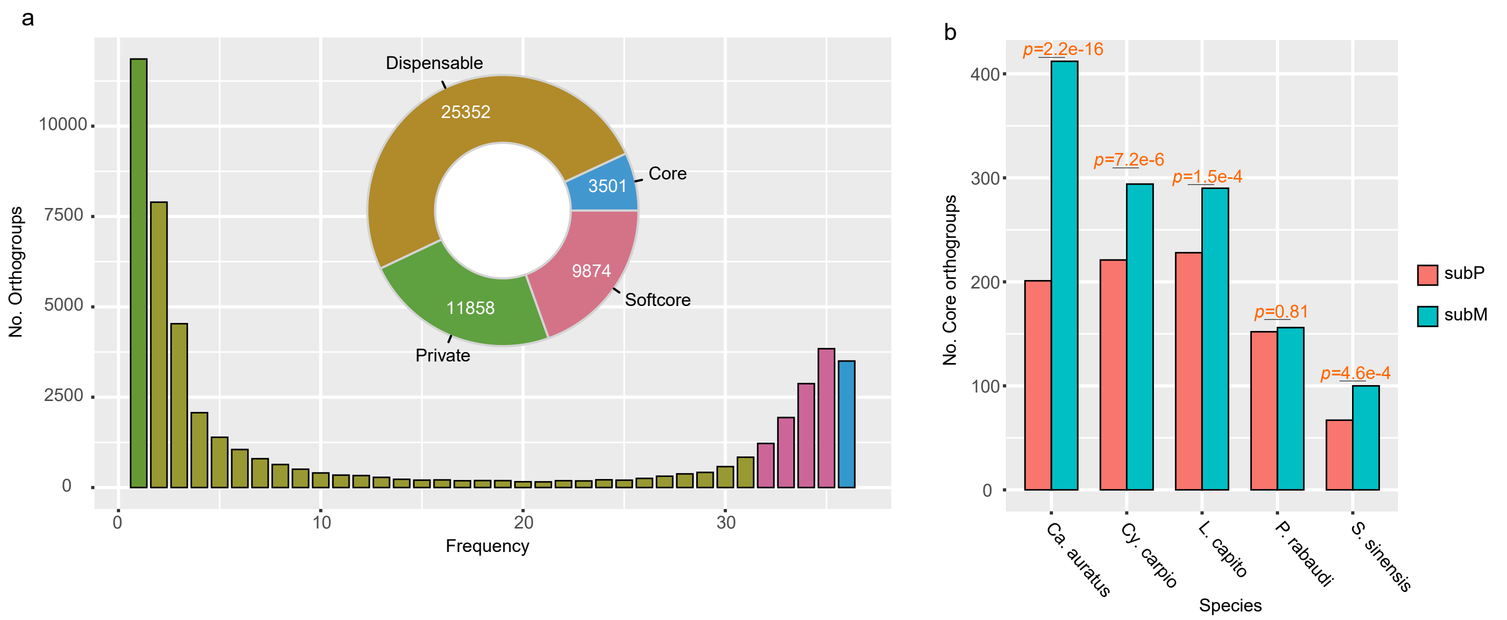

Supplementary Fig. 37. Pan-gene analysis. **a** Frequency of orthogroups. The pie chart shows the proportion of core (shared by all 36 samples), softcore (shared by > 90% samples but not all), dispensable (shared by more than one but ≤ 90% samples), and private genes (present in only one sample) in those genomes. **b** Number of core genes that exists only in subP and subM of allopolyploids. There tends to be a statistically significant number of core genes biased distribution toward the subM in all species except *P. rabaudi* (X^2 text; p-value≤4.6e-4).

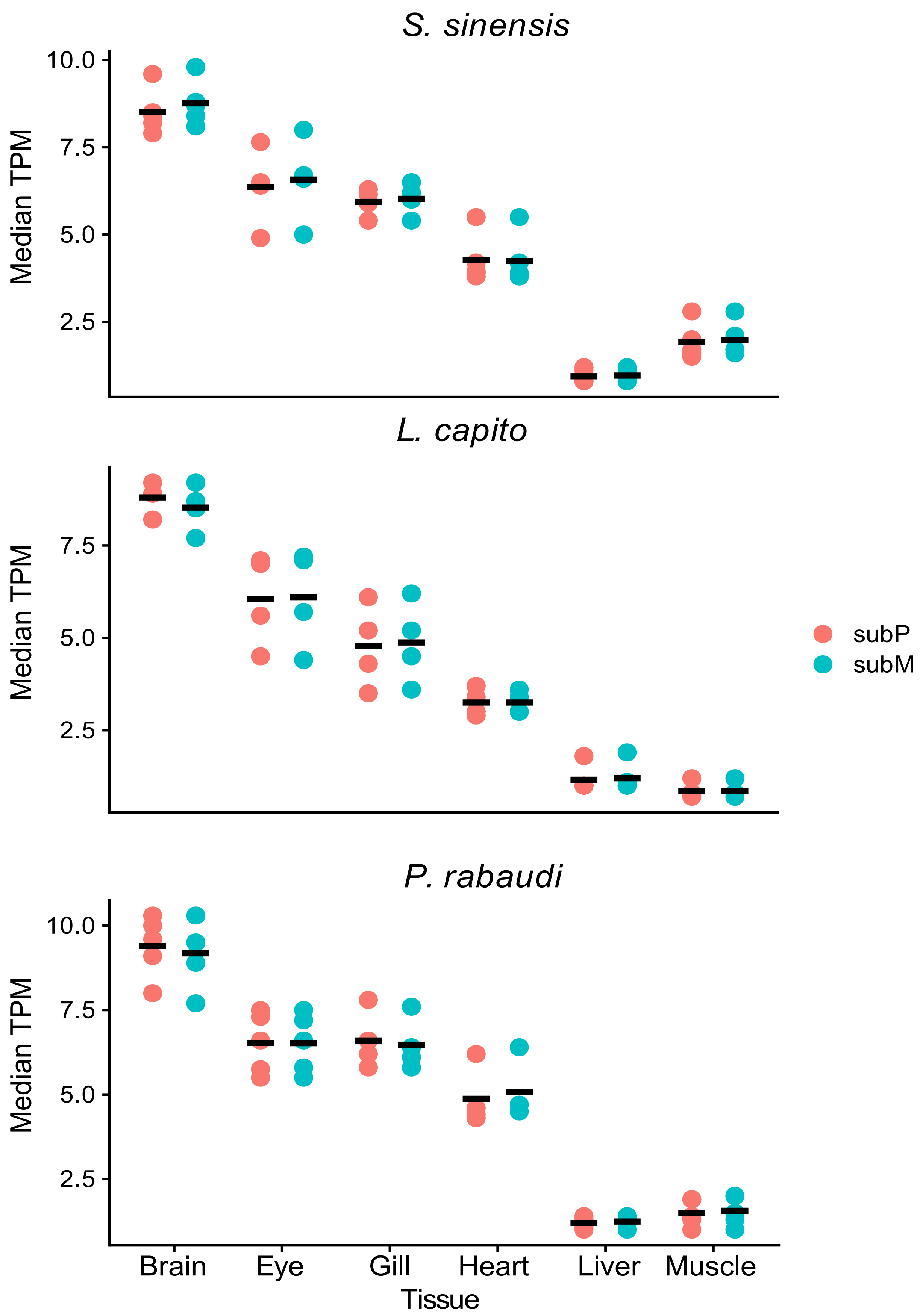

Supplementary Fig. 38. Median TPM of genes from three to five replicates of RNAseq across six tissue types in *S. sinensis*, *L. capito*, and *P. rabaudi*. Median TPM values displayed are separated by subP (red) and subM (blue) with the average across all three to five replicates for each subgenome/tissue indicated by horizontal black bars.

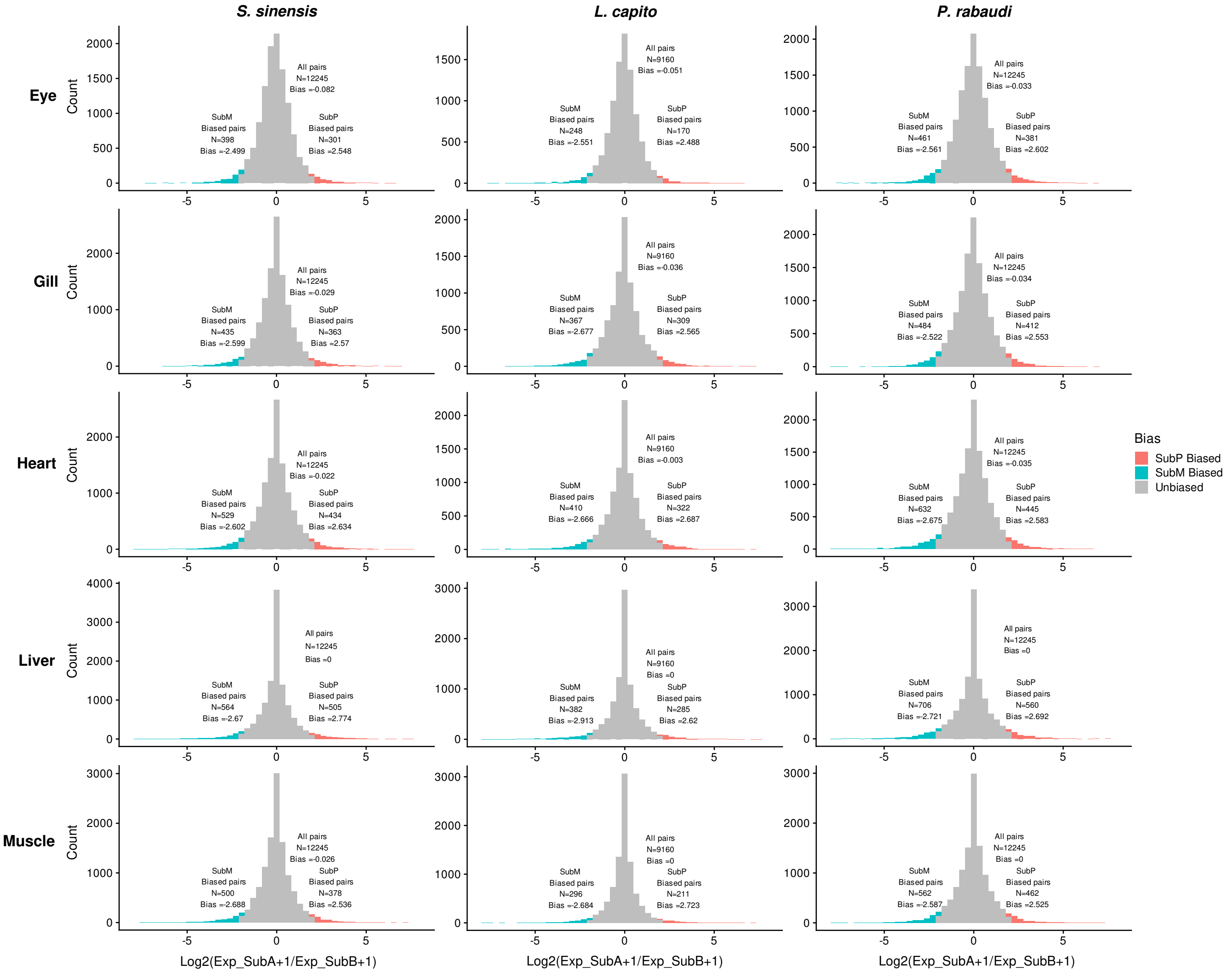

Supplementary Fig. 39. Expression of homoeolog pairs plotted as subP biased synteologs (red), subM biased syntelogs (blue) and unbiased synteologs (grey) was shown in five tissues.

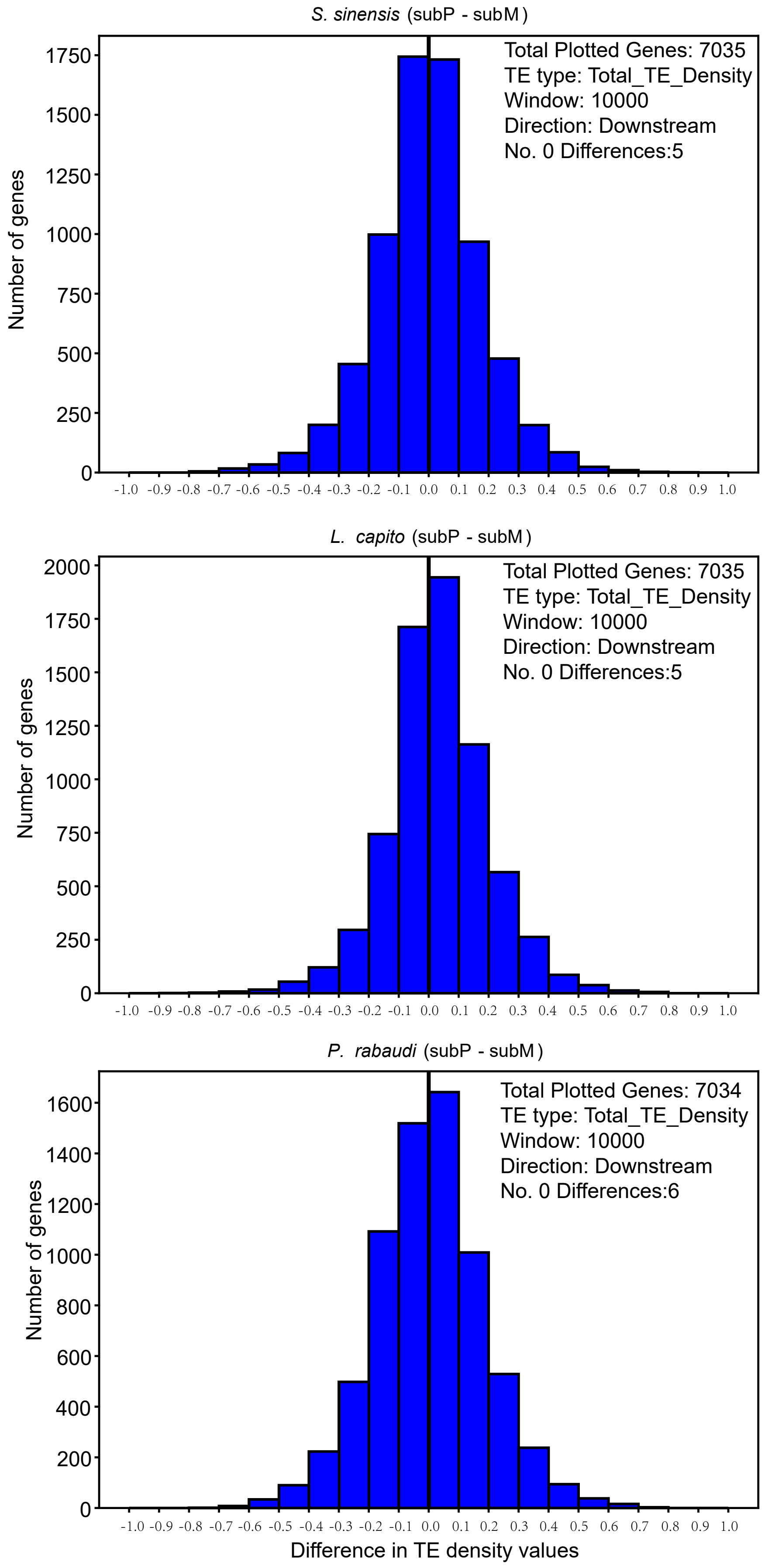

Supplementary Fig. 40. Histograms of differences in TE density values downstream of subP and subM syntelogs of *S. sinensis*, *L. capito*, and *P. rabaudi*. The density values were calculated for all TEs in a 10,000 bp window downstream of genes and difference values were calculated by subtracting TE density of subM syntelogs from subP syntelogs. Negative values represent higher TE density for syntelogs in the subM, whereas positive values reflect higher TE density for the subP syntelogs.

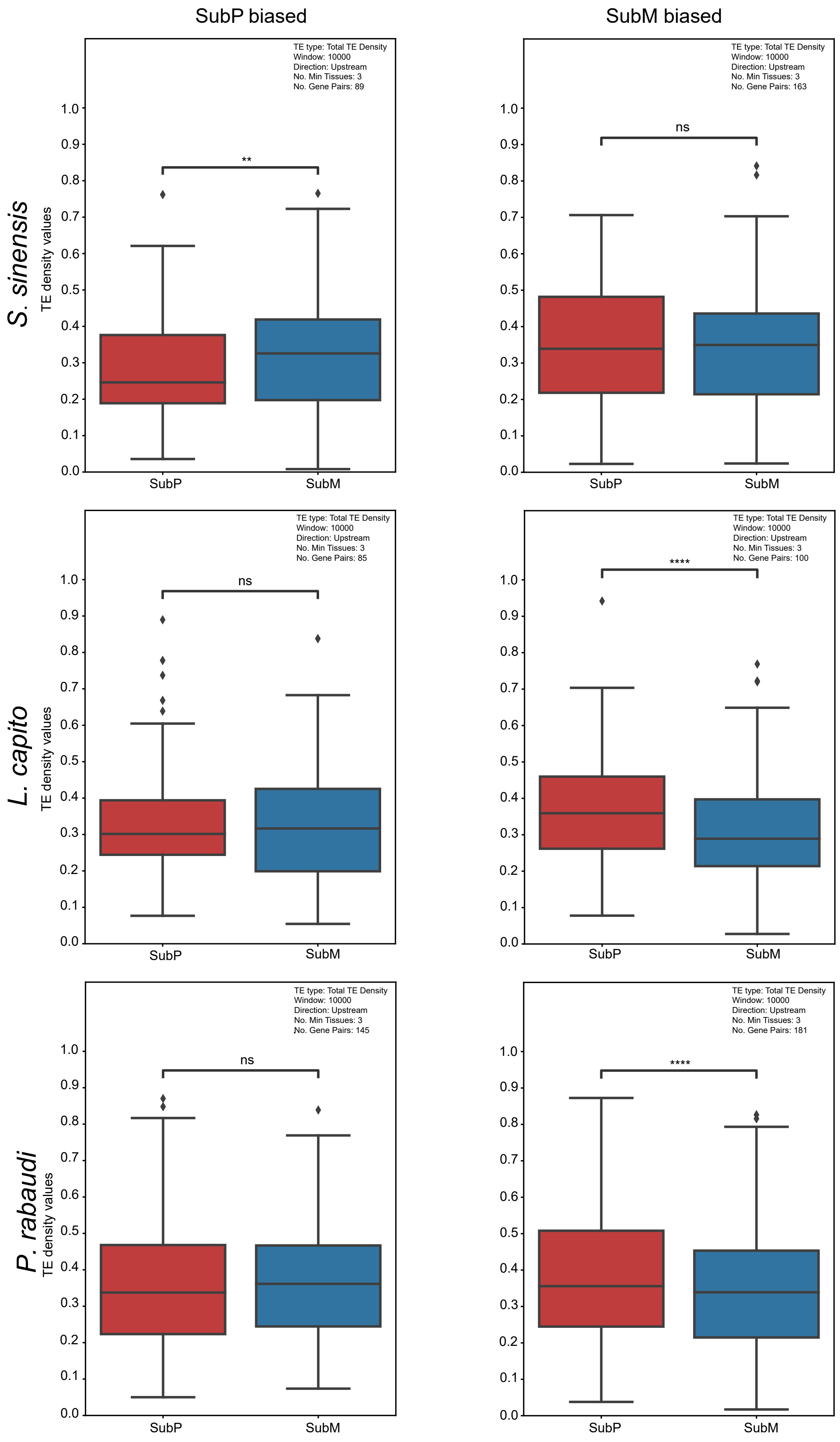

Supplementary Fig. 41. Boxplots of TE density values for genes exhibiting biased expression in subP (left column) and subM (right column). TE density of syntelogs in subP (red) and subM (blue) for *S. sinensis*, *L. capito*, and *P. rabaudi* were plotted for 10,000 bp windows upstream of gene start sites. Two-sample t-test results are shown as non-significant (ns), $p < 0.005$ (**) and $p < 0.00005$ (****).

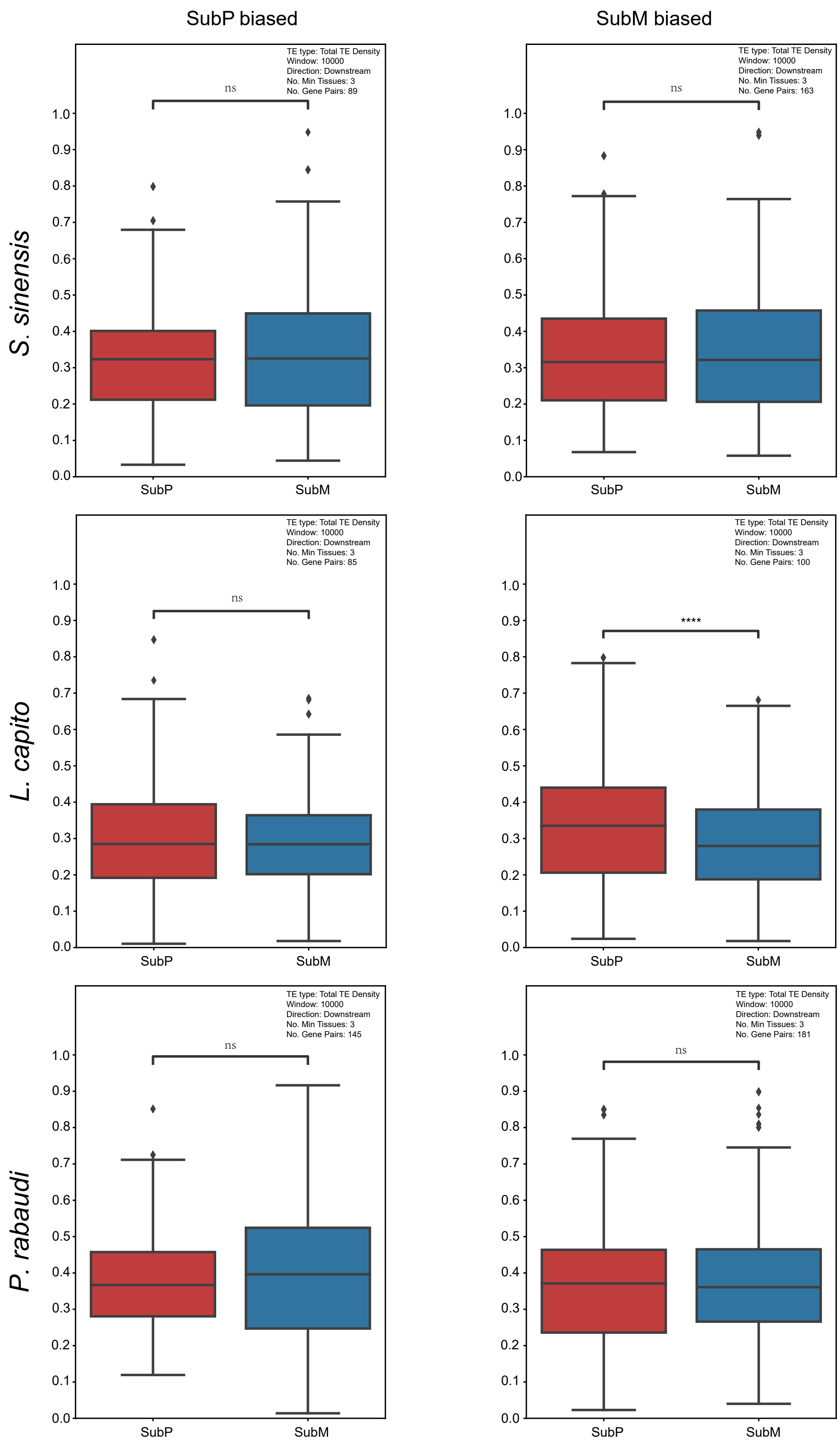

Supplementary Fig. 42. Boxplots of TE density values for genes exhibiting biased expression in subP (left column) and subM (right column). TE density of syntelogs in subP (red) and subM (blue) for *S. sinensis*, *L. capito*, and *P. rabaudi* were plotted for 10,000 bp windows downstream of gene start sites. Two-sample t-test results are shown as non-significant (ns) and $p < 0.00005$ (****).

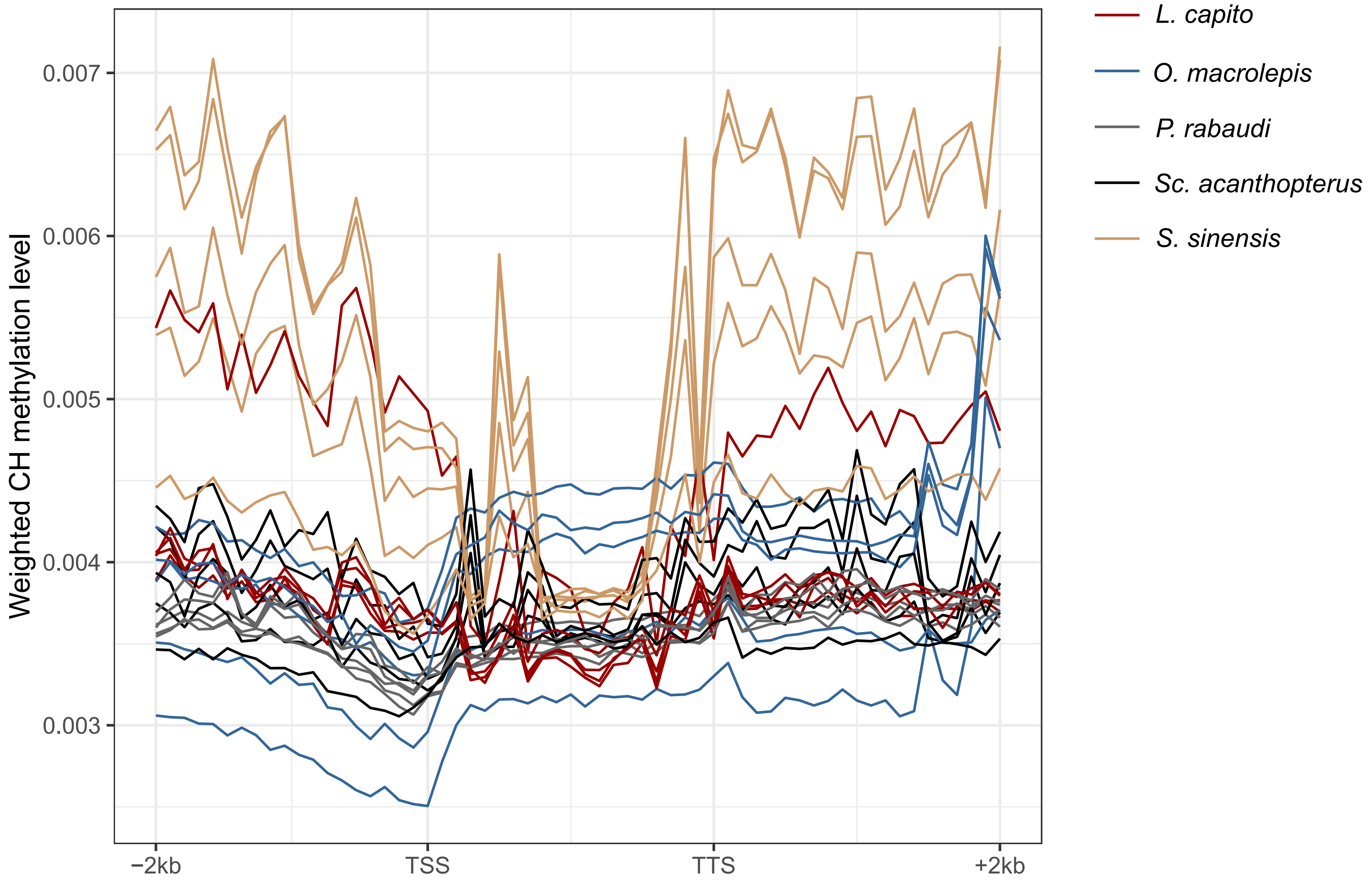

Supplementary Fig. 43. Methylation levels at the CH sites of two diploid ancestors *O. macrolepis* and *Sc. acanthopterus* and three allotetraploids *S. sinensis*, *L. capito*, and *P. rabaudi*. The x axis represented the gene body (TSS = transcription start site and TTS = transcription termination site) and 2 kb upstream and downstream region. The y axis showed the weighted CH methylation level.

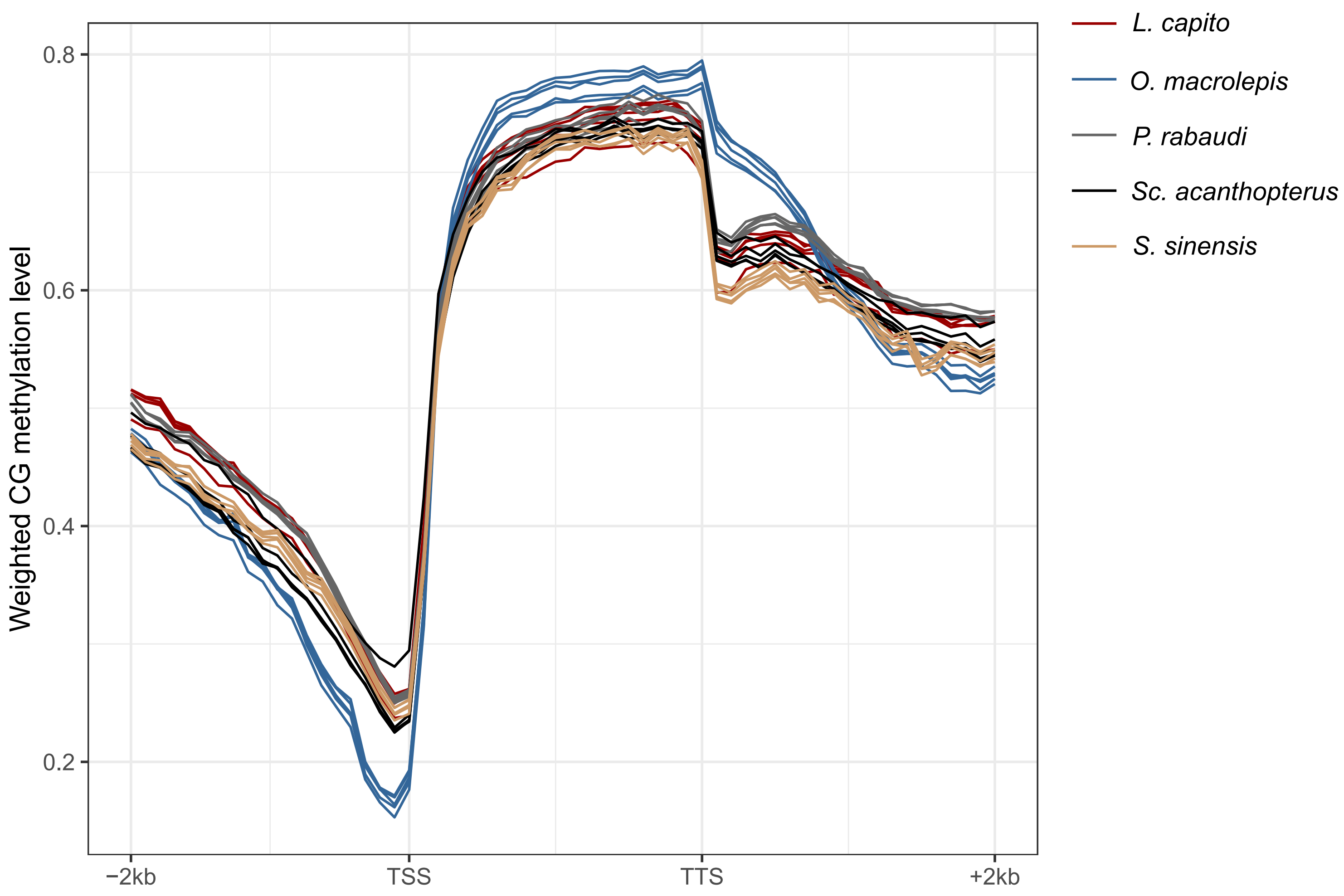

Supplementary Fig. 44. CG methylation pattern of two diploid ancestors *O. macrolepis* and *Sc. acanthopterus* and three allotetraploids *S. sinensis*, *L. capito*, and *P. rabaudi*. The x axis showed the gene body (TSS and TTS) and 2 kb upstream and downstream region. The y axis was the weighted CG methylation level.

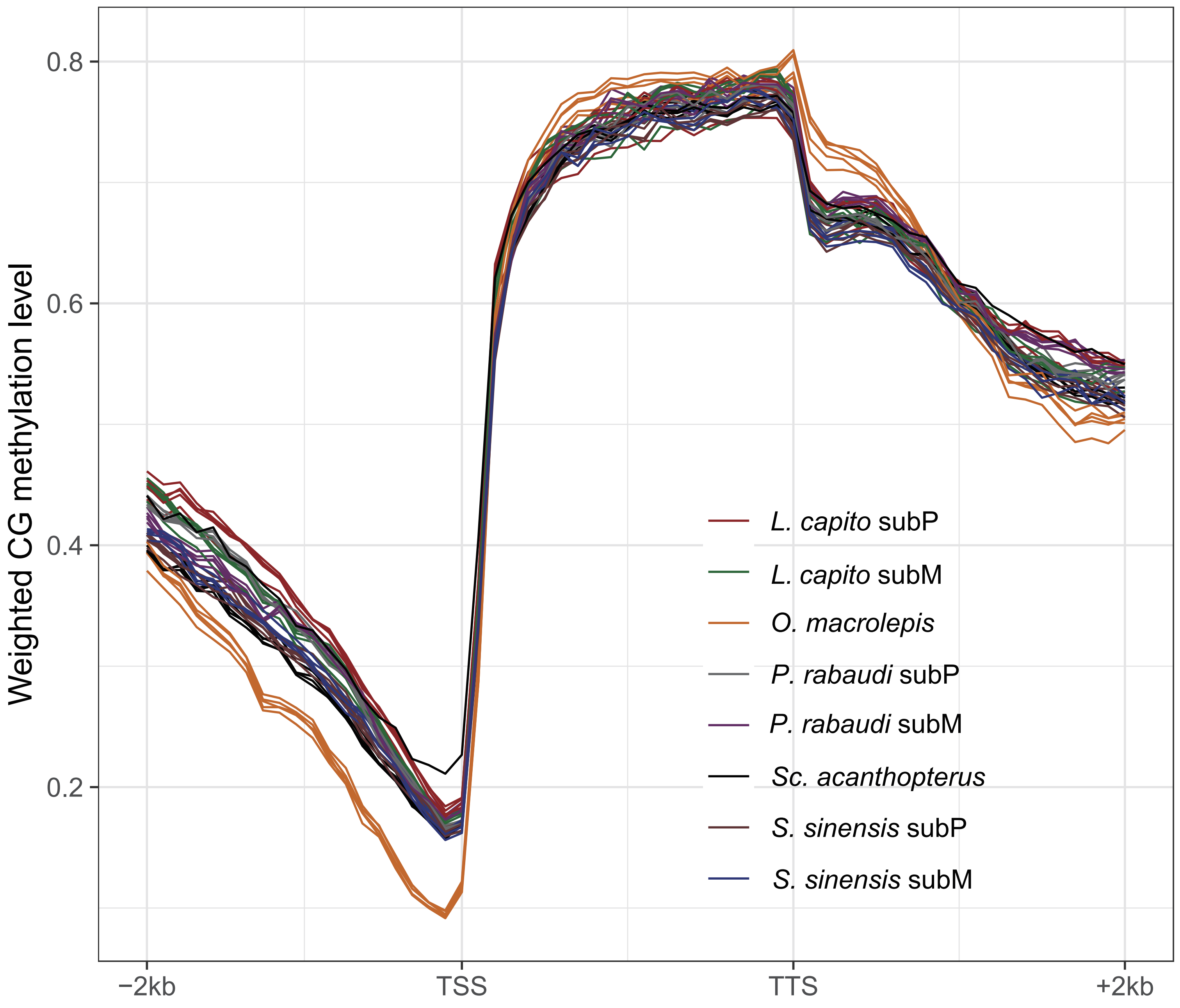

Supplementary Fig. 45. CG methylation pattern of 7040 genes with a 1:1:2:2:2 relationship (1 *O. macrolepis* gene, 1 *Sc. acanthopterus* gene, 2 *S. sinensis* genes, 2 *L. capito* genes and 2 *P. rabaudi* genes). The x and y axis represented the gene body (TSS and TTS) and 2 kb upstream and downstream region and weighted CG methylation level, respectively.

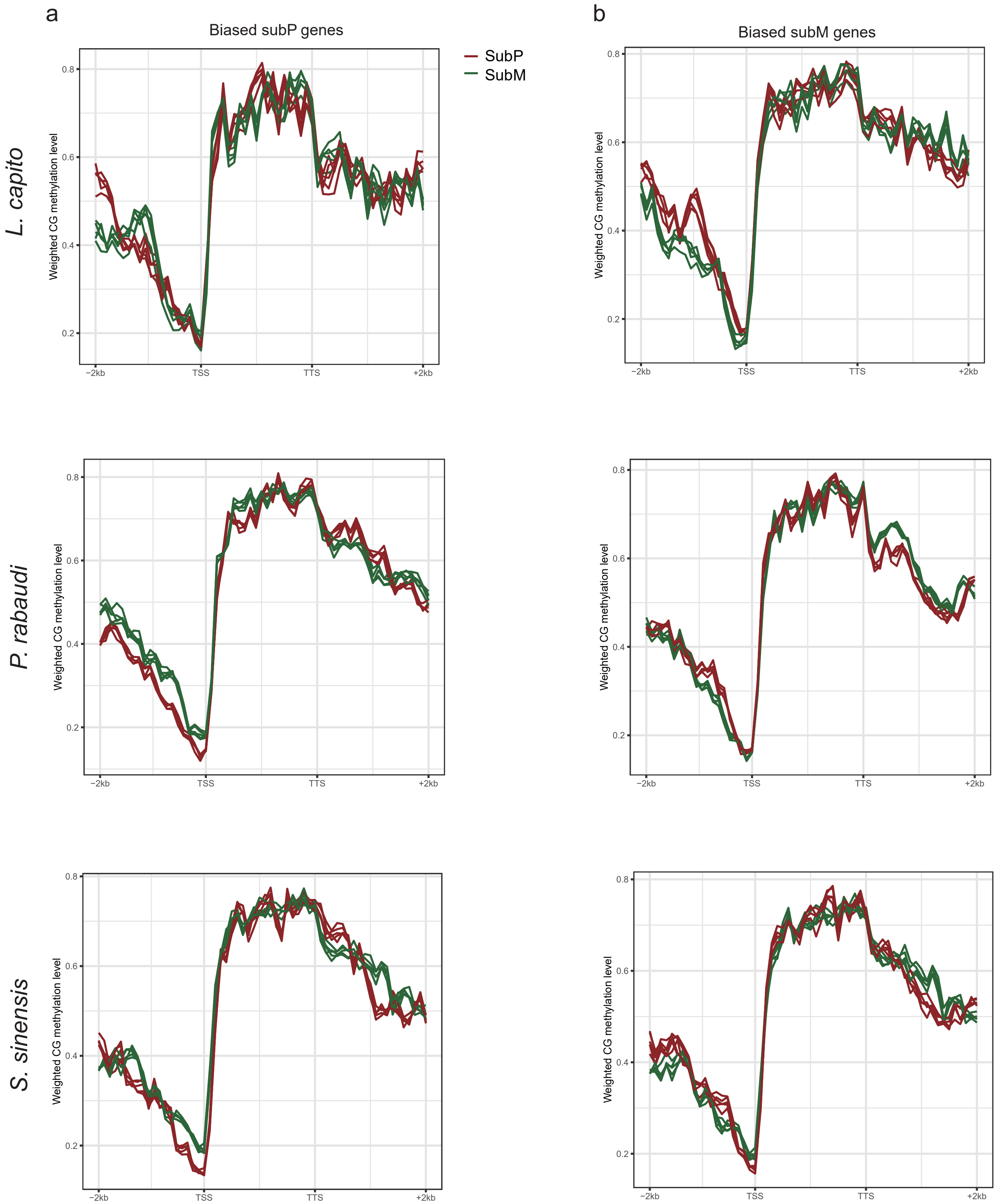

Supplementary Fig. 46. CG methylation levels of subP (**a**) or subM (**b**) biased expression genes in the muscle tissue of *L. capito*, *P. rabaudi* and *S. sinensis*. The x axis was the gene body and 2 kb upstream and downstream region. The y axis indicated the weighted CG methylation level.

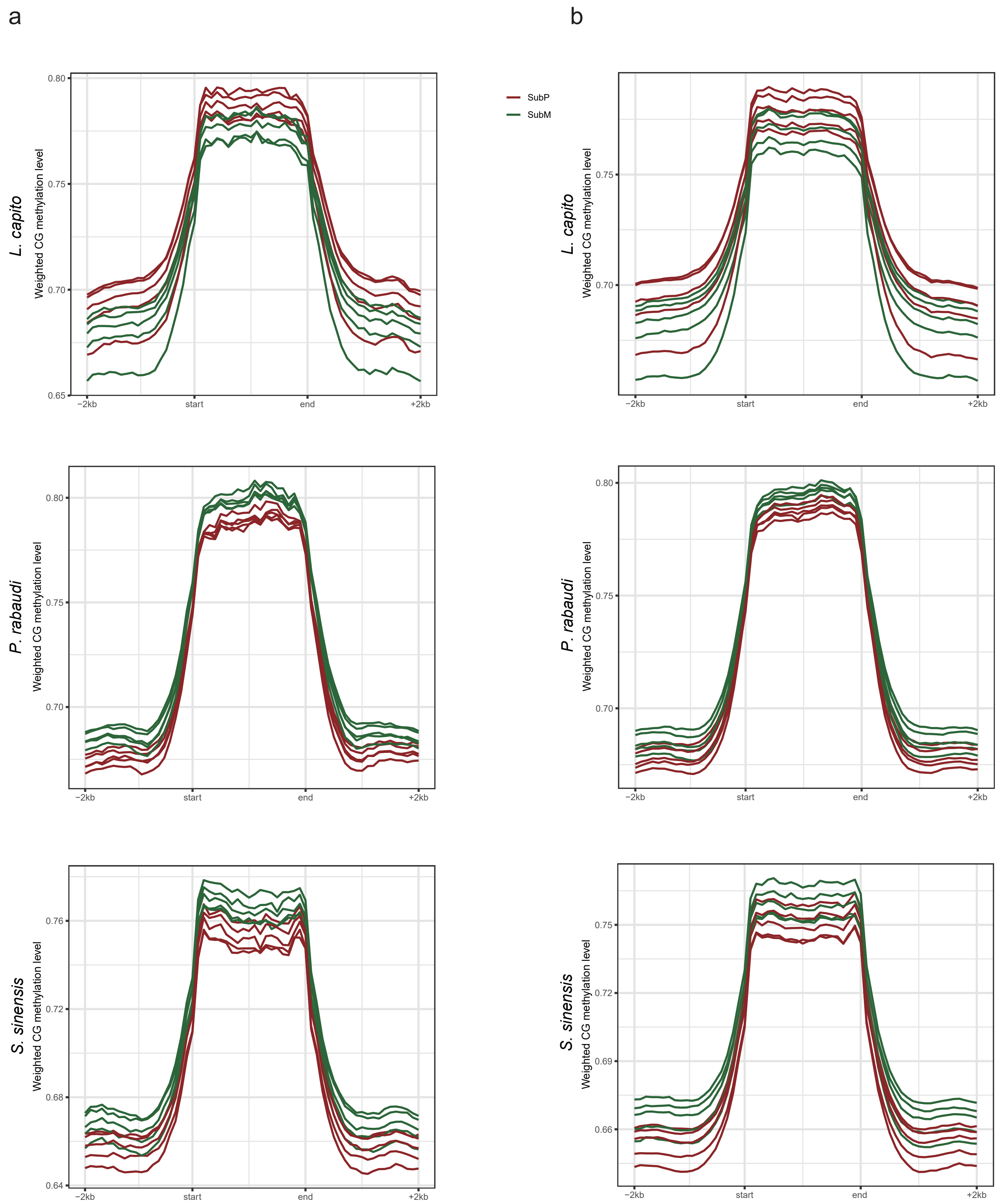

Supplementary Fig. 47. Comparision of TE mCG levels between subP (red) and subM (green). CG methylation of TEs that are in 1kb vicinity of 7040 positionally conserved syntenic ohnologs (**a**) and at the whole genome level (**b**). The x axis was the TE and 2 kb upstream and downstream region. The y axis indicated the weighted CG methylation level of TEs.

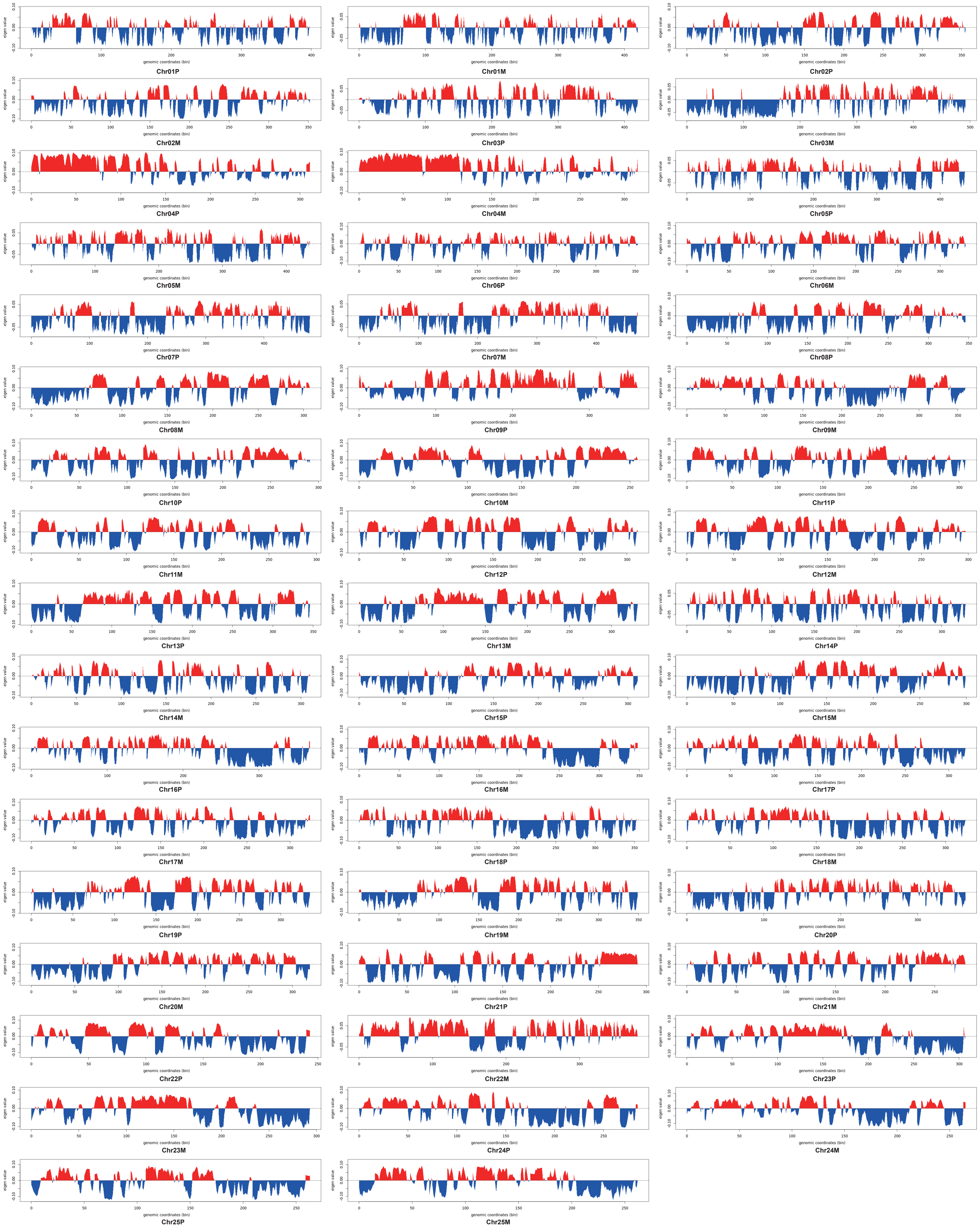

Supplementary Fig. 48. Global A/B compartments identified in the *L. capito* genome using the PCA-based method.

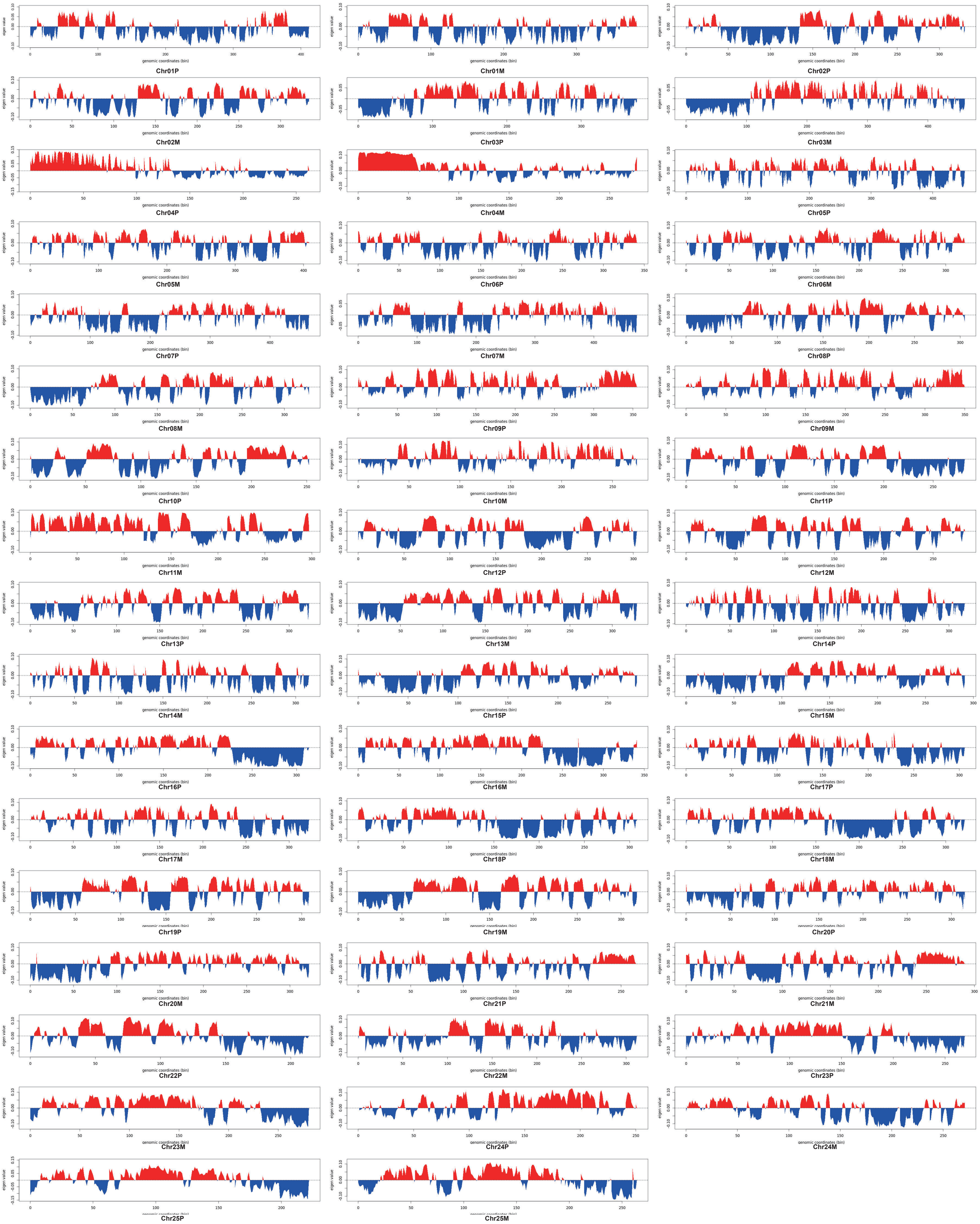

Supplementary Fig. 49. Global A/B compartments found in the *P. rabaudi* genome using the PCA-based method.

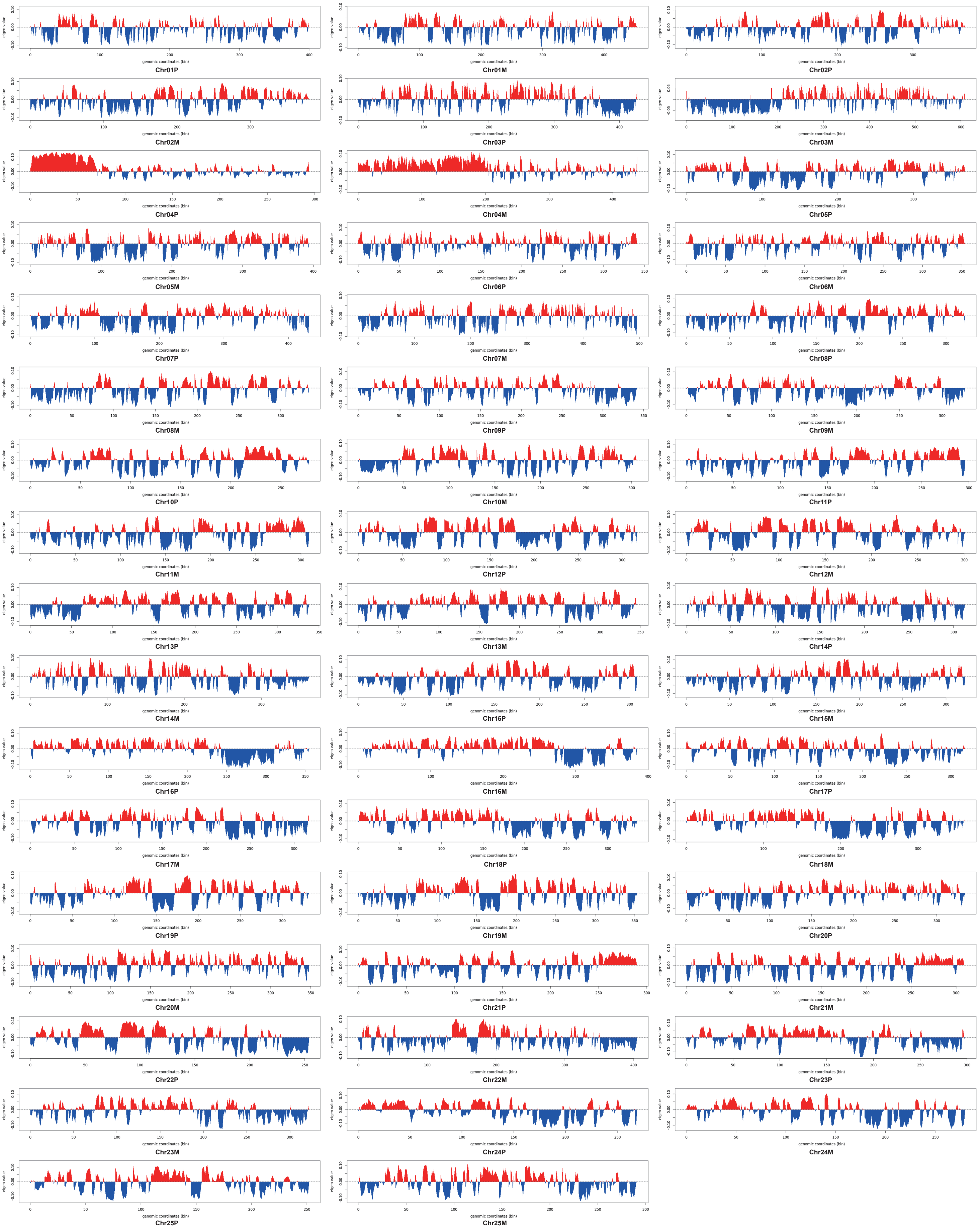

Supplementary Fig. 50. Global A/B compartments obtained from the *S. sinensis* genome using the PCA-based method.

Supplementary Table 1. Detail information of sequenced species in this study.

Species	Sampling sites and Breeder	Common name	Artificial breeding/wild
**Xenocyprididae**			
Zacco platypus	Liaohe River, Yingkou city, Liaoning province, China	freshwater minnow	Wild
Aphyocypris chinensis	Dongjiang, Shaoxing city, Zhejiang province, China	green chub	Wild
Mylopharyngodon piceus	Longyun Aquatic Products, Kunming city, Yunnan province, China **Breeder:** Jian-Chao Jin	black carp	Artificial breeding
Squaliobarbus curriculus	Junda Aquatic Products, Foshang city, Guangdong province, China **Breeder:** Yong-Liang Zhang	barbel chub	Artificial breeding
Anabarilius duoyiheensis	Duoyi River, Luoping city, Yunnan province, China	Duoyi River white fish	Wild
Distoechodon tumirostris	Breeding Farm of Guyu, Liling city, Hunan province, China **Breeder:** Liang-Yu Peng	round snout	Artificial breeding
**Tincidae**			
Tinca tinca	Longyun Aquatic Products, Kunming city, Yunnan province, China **Breeder:** Jian-Chao Jin	tench	Artificial breeding
**Gobionidae**			
Belligobio pengxianensis	Qingbaijiang, Chengdu city, Sichuan province, China		Wild
Pseudorasbora parva	Yunnan province, China	stone moroko	Wild
Ladislavia taczanowskii	Pushi River, Dandong city, Liaoning province, China	Tachanovsky's gudgeon	Wild
Gobiobotia tungi	Qiandao Lake, Chun'an city, Zhejiang province, China		Wild
Progobiobotia guilingensis	Lijiang River, Guilin city, Guangxi province, China		Wild
**Acheilognathidae**			

Rhodeus sinensis	Taihu Lake, Huzhou city, Zhejiang province, China	the Chinese bitterling	Wild
Acheilognathus tonkinensis	Red River, Hechi city, Guangxi province, China	the rainbow bitterling	Wild
**Cyprinidae**			
Onychostoma macrolepis #	Julong Aquatic Products, Linyi city, Shandong province, China **Breeder:** Yu-Feng Wang		Artificial breeding
Scaphiodonichthys acanthopterus	Lancang River, Xishuangbanna, Yunnan province, China		Wild
Crossocheilus oblongus	Borneo, Indonesia	Siamese flying fox	Wild
Sinilabeo rendahli	Fourth Sister Aquatic Products, Chengdu city, Sichuan province, China **Breeder:** Si He		Artificial breeding
Semilabeo prochilus	Nanpan River, Xingyi city, Guizhou province, China		Wild
Procypris rabaudi	Tiangui Aquaculture Farm, Meishan city, Sichuan province, China **Breeder:** Tian-Gui He	rock carp	Artificial breeding
Spinibarbus sinensis	Tiangui Aquaculture Farm, Meishan city, Sichuan province, China **Breeder:** Tian-Gui He	qingbo	Artificial breeding
Luciobarbus capito	Xiaozu Aquaculture Farm, Meishan city, Sichuan province, China **Breeder:** Xiang Gao	Aral barbel	Artificial breeding

#Muscle of *Onychostoma macrolepis* was obtained for whole-genome bisulfite sequencing; RNA-seq sequencing of its six tissues were also performed.

Supplementary Table 2. Summary of sequencing data for assembly of the genomes.

Species	Illumina reads				PacBio reads			
	Insert size (bp)	Total data (Gb)	Mean read length (bp)	Sequence coverage (×)	Insert size (bp)	Total data (Gb)	Mean read length (bp)	Sequence coverage (×)
A. tonkinensis	350	54.9	350	66.35	15,000	33.71	16,189	40.74
An. duoyiheensis	350	65.9	350	64.11	15,000	20.15	16,626	19.60
Ap. chinensis	350	53.1	350	61.13	15,000	25.38	16,959	29.21
B. pengxianensis	350	57.5	350	54.75	15,000	39.64	19,675	37.74
C. oblongus	350	61.8	350	44.46	15,000	33.74	16,379	24.27
D. tumirostris	350	56.3	350	56.88	15,000	21.60	15,780	21.82
G. tungi	350	74.5	350	92.68	15,000	25.18	16,617	31.32
La. taczanowskii	350	50.5	350	45.31	15,000	27.15	15,675	24.36
L. capito	350	103.3	350	59.73	15,000	59.91	20,395	34.23
M. piceus	350	58.1	350	65.53	15,000	32.03	17,720	36.13
Pr. guilingensis	350	74.8	350	88.83	15,000	23.89	14,856	28.37
P. rabaudi	350	153.2	350	94.96	15,000	50.43	13,326	30.94
Ps. parva	350	70.6	350	56.46	15,000	47.00	16,119	37.59
R. sinensis	350	61.6	350	71.31	15,000	28.41	22,235	32.89
Sc. acanthopterus	350	67.1	350	82.82	15,000	37.31	16,698	46.06
Se. prochilus	350	70.2	350	56.26	15,000	33.34	17,126	26.72
Si. rendahli	350	58.7	350	53.30	15,000	36.12	18,110	32.79
S. sinensis	350	102.8	350	56.54	15,000	59.38	16,987	32.37
Sq. curriculus	350	60.7	350	66.62	15,000	33.96	15,924	37.27
T. tinca	350	53.1	350	53.03	15,000	34.60	16,839	34.55
Z. platypus	350	92.2	350	112.95	15,000	32.85	17,309	40.25

Supplementary Table 3. Summary of genome assemblies of twenty-one species.

Species	Title	Total length	Total number	Max length	Min length	N50 length	N90 length
A. tonkinensis	Contig	852,489,282	269	32,237,854	4,372	12,201,717	2,717,980
An. duoyiheensis	Contig	1,066,577,311	331	39,295,935	16,778	11,733,698	2,475,941
Ap. chinensis	Contig	905,580,794	250	36,051,031	1,960	19,070,385	3,680,507
B. pengxianensis	Contig	1,059,753,831	229	52,383,806	6,410	22,184,699	6,363,258
C. oblongus	Contig	1,354,293,807	208	43,807,734	3,075	17,048,025	4,507,251
D. tumirostris	Contig	1,004,000,895	146	51,452,208	16,617	34,074,188	11,081,630
G. tungi	Contig	809,952,474	135	43,348,994	15,847	28,687,375	9,255,376
La. taczanowskii	Contig	1,180,167,056	900	20,720,747	8,400	4,822,149	913,001
L. capito	Contig	1,711,978,371	183	48,354,785	3,445	32,739,442	23,203,500
M. piceus	Contig	888,063,602	92	74,811,575	16,615	33,902,650	26,986,539
Pr. guilingensis	Contig	852,817,038	179	44,603,978	2,177	25,354,504	7,715,807
P. rabaudi	Contig	1,642,167,810	572	33,828,682	11,013	16,097,078	1,958,542
Ps. parva	Contig	1,273,287,385	268	33,052,391	16,353	10,622,733	2,740,494
R. sinensis	Contig	902,936,778	287	41,811,712	13,121	27,608,729	4,542,592
Sc. acanthopterus	Contig	810,168,215	96	49,176,325	16,527	31,627,781	24,492,193
Se. prochilus	Contig	1,323,874,878	355	41,324,975	2,468	17,175,830	4,186,396
Si. rendahli	Contig	1,133,144,711	142	45,933,930	10,838	38,276,418	11,622,219
S. sinensis	Contig	1,834,339,152	359	48,733,901	7,972	27,166,239	5,861,860
Sq. curriculus	Contig	944,349,790	132	40,974,083	15,155	24,429,182	11,241,868
T. tinca	Contig	1,017,523,531	176	55,735,399	8,130	27,533,735	7,401,790
Z. platypus	Contig	832,640,428	178	42,058,691	18,959	23,286,023	6,563,167

Supplementary Table 4. Estimation of the genome size using 17-mer distribution analysis.

Species	*K* -mer number	Genome size (Mb)	Revised genome size (Mb)	Heterozygous rate (%)	Repeat rate (%)
A. tonkinensis	39,482,506,269	840.05	827.49	0.58	46.36
An. duoyiheensis	53,376,760,265	1046.6	1027.89	0.33	54.46
Ap. chinensis	42,299,190,119	881.23	868.6	0.79	47.73
B. pengxianensis	45,796,475,485	1,065.03	1,050.31	0.42	55.37
C. oblongus	47,836,714,168	1,406.96	1,390.07	0.99	61.73
D. tumirostris	45,230,095,376	1005.11	989.86	0.4	53.43
G. tungi	58,880,249,640	817.78	803.88	0.41	44.55
La. taczanowskii	40,723,843,533	1,131.22	1,114.52	0.46	57.72
L. capito	82,122,315,237	1,747.28	1,729.44	0.26	68.04
M. piceus	46,740,942,556	898.86	886.59	0.26	50.63
Pr. guilingensis	59,124,713,573	856.88	842.08	0.62	45.75
P. rabaudi	120,715,983,953	1,631.30	1,613.34	0.38	60.31
Ps. parva	55,733,836,016	1,266.68	1,250.34	0.64	59.31
R. sinensis	49,081,886,650	876.46	863.85	0.93	47.4
Sc. acanthopterus	47,860,751,637	825.19	813.63	0.33	46.3
Se. prochilus	54,482,247,533	1,267.03	1,247.84	0.4	61.7
Si. rendahli	45,906,630,802	1,119.67	1,101.28	0.36	58.07
S. sinensis	82,695,020,082	1,837.67	1,818.34	0.48	66.05
Sq. curriculus	48,049,248,202	924.02	911.18	0.54	50.01
T. tinca	42,700,330,212	1,016.67	1,001.39	0.49	54.05
Z. platypus	73,512,184,785	825.98	816.26	0.59	45.43

Supplementary Table 5. BUSCO evaluation of the completeness and accuracy of the genomes.

Species	Complete BUSCOs (%)	Complete and single-copy BUSCOs (%)	Complete and duplicated BUSCOs (%)	Fragmented BUSCOs (%)	Missing BUSCOs (%)	Total number of BUSCOs
A. tonkinensis	95.90%	91.20%	4.70%	1.90%	2.20%	4584
An. duoyiheensis	95.70%	91.10%	4.60%	2.10%	2.20%	4584
Ap. chinensis	95.60%	90.00%	5.60%	2.10%	2.30%	4584
B. pengxianensis	95.70%	91.90%	3.80%	2.10%	2.20%	4584
C. oblongus	95.70%	92.10%	3.60%	2.00%	2.30%	4584
D. tumirostris	95.90%	91.40%	4.50%	2.10%	2.00%	4584
G. tungi	95.30%	90.90%	4.40%	2.20%	2.50%	4584
La. taczanowskii	94.40%	89.00%	5.40%	2.70%	2.90%	4584
L. capito	96.60%	24.90%	71.70%	1.10%	2.30%	4584
M. piceus	96.00%	92.30%	3.70%	2.00%	2.00%	4584
Pr. guilingensis	95.50%	91.30%	4.20%	2.10%	2.40%	4584
P. rabaudi	96.50%	31.90%	64.60%	1.40%	2.10%	4584
Ps. parva	94.80%	90.70%	4.10%	2.40%	2.80%	4584
R. sinensis	95.70%	90.40%	5.30%	2.10%	2.20%	4584
Sc. acanthopterus	96.20%	92.40%	3.80%	1.70%	2.10%	4584
Se. prochilus	95.70%	91.90%	3.80%	2.30%	2.00%	4584
Si. rendahli	96.00%	91.90%	4.10%	1.80%	2.20%	4584
S. sinensis	96.60%	24.50%	72.10%	1.10%	2.30%	4584
Sq. curriculus	95.80%	90.60%	5.20%	1.90%	2.30%	4584
T. tinca	95.80%	91.80%	4.00%	1.90%	2.30%	4584
Z. platypus	95.90%	91.30%	4.60%	2.10%	2.00%	4584

Supplementary Table 6. Statistics of Hi-C mapping of the *S. sinensis* genome.

Clean Paired-end Reads	511,202,827
Unmapped pairs-end Reads	20,288,335
Unmapped pairs-end Reads Rate (%)	3.969
Pairs-end Reads with singleton	126,649,959
Pairs-end Reads with singleton Rate (%)	24.775
Multi Mapped pairs-end Reads	0
Multi Mapped Ratio (%)	0
Unique Mapped paird-end Reads	192,162,529
Unique Mapped paird-end Reads Rate (%)	37.59
Statistics of valid reads	
Unique Mapped Paird-end Reads	192,162,529
Dangling End Pair-end Reads	69,106,617
Dangling End. Rate (%)	35.962
Self Circle Paird-end Reads	1,316,086
Self Circle Rate (%)	0.685
Dumped Paird-end Reads	130,738
Dumped Rate (%)	0.068
Interaction Paired-end Reads	98,259,877
Interaction Rate(%)	51.134
Lib Valid Paired-end Reads	67,349,935
Lib valid Rate (%)	68.543
Lib Dup (%)	31.457

Supplementary Table 7. Statistics of Hi-C mapping of the *L. capito* genome.

Clean Paired-end Reads	530,105,378
Unmapped pairs-end Reads	13,182,640
Unmapped pairs-end Reads Rate(%)	2.487
Pairs-end Reads with singleton	66,420,608
Pairs-end Reads with singleton Rate(%)	12.53
Multi Mapped pairs-end Reads	0
Multi Mapped Ratio(%)	0
Unique Mapped paird-end Reads	222,495,670
Unique Mapped paird-end Reads	41.972
Statistics of valid reads	
Unique Mapped Paird-end Reads	222,495,670
Dangling End Pair-end Reads	30,382,857
Dangling End. Rate(%)	13.655
Self Circle Paird-end Reads	2,215,387
Self Circle Rate(%)	0.995
Dumped Paird-end Reads	50,048
Dumped Rate(%)	0.022
Interaction Paired-end Reads	174,876,480
Interaction Rate(%)	78.598
Lib Valid Paired-end Reads	123,501,434
Lib valid Rate(%)	70.622
Lib Dup(%)	29.378

Supplementary Table 8. Statistics of Hi-C mapping of the *P. rabaudi* genome.

Clean Paired-end Reads	587,620,065
Unmapped pairs-end Reads	12,526,620
Unmapped pairs-end Reads Rate(%)	2.132
Pairs-end Reads with singleton	79,239,660
Pairs-end Reads with singleton Rate(%)	13.485
Multi Mapped pairs-end Reads	0
Multi Mapped Ratio(%)	0
Unique Mapped paird-end Reads	273,052,557
Unique Mapped paird-end Reads	46.468
Statistics of valid reads	
Unique Mapped Paird-end Reads	273,052,557
Dangling End Pair-end Reads	39,287,331
Dangling End. Rate(%)	14.388
Self Circle Paird-end Reads	1,714,916
Self Circle Rate(%)	0.628
Dumped Paird-end Reads	59,778
Dumped Rate(%)	0.021
Interaction Paired-end Reads	212,518,334
Interaction Rate(%)	77.83
Lib Valid Paired-end Reads	151,997,863
Lib valid Rate(%)	71.522
Lib Dup(%)	28.478

Supplementary Table 9. Statistical data of 50 chromosomes of *S. sinensis*.

ChrID	Anchored_ctg	Length (bp)	Gene number	ChrID	Anchored_ctg	Length (bp)	Gene number
Chr01P	25	40,048,438	1,134	Chr13M	9	34,622,671	913
Chr01M	66	45,400,670	1,232	Chr14P	3	31,327,747	844
Chr02P	58	36,983,292	1,109	Chr14M	88	36,262,177	915
Chr02M	78	38,086,976	1,165	Chr15P	25	30,892,536	854
Chr03P	90	42,702,752	1,286	Chr15M	23	32,281,118	862
Chr03M	105	60,952,779	1,809	Chr16P	81	35,579,278	1,054
Chr04P	16	29,456,046	828	Chr16M	50	38,558,027	1,121
Chr04M	90	43,812,607	1,196	Chr17P	48	31,617,493	856
Chr05P	53	36,844,798	1,095	Chr17M	14	31,795,286	861
Chr05M	38	39,430,148	1,105	Chr18P	40	33,530,787	804
Chr06P	8	34,116,691	986	Chr18M	13	36,111,743	796
Chr06M	58	35,411,886	965	Chr19P	36	33,280,341	953
Chr07P	55	43,249,945	1,079	Chr19M	52	35,306,529	999
Chr07M	18	49,662,784	1,293	Chr20P	52	33,404,830	965
Chr08P	2	32,288,098	1,011	Chr20M	43	34,891,650	949
Chr08M	42	33,452,449	1,022	Chr21P	40	29,010,266	856
Chr09P	78	34,259,335	856	Chr21M	24	31,024,171	914
Chr09M	14	32,737,962	808	Chr22P	75	25,448,094	688
Chr10P	32	27,862,358	868	Chr22M	108	40,516,179	914
Chr10M	33	30,630,240	885	Chr23P	80	29,863,141	904
Chr11P	25	29,617,316	845	Chr23M	36	32,222,432	933
Chr11M	32	30,973,789	812	Chr24P	20	26,988,831	746
Chr12P	15	31,797,855	836	Chr24M	10	28,009,647	779
Chr12M	45	30,117,390	845	Chr25P	50	25,256,613	780
Chr13P	21	33,838,460	932	Chr25M	68	29,159,053	905

Total number of contigs: 4,343
Total length of contigs (bp): 1,834,334,809
Total number of anchored contgis: 2,185
Total length of chromosome level assembly (bp): 1,730,695,704
Number of unanchored contigs: 2,158
Length of unanchored contigs (bp): 103,852,605
Anchor rate (%): 94.34

Supplementary Table 10. Statistical data of 50 chromosomes of *P. rabaudi*.

ChrID	Anchored_ctg	Length (bp)	Gene number	ChrID	Anchored_ctg	Length (bp)	Gene number
Chr01P	185	41,274,036	1,090	Chr13M	70	32,931,668	889
Chr01M	100	38,395,085	1,078	Chr14P	54	31,878,092	819
Chr02P	72	33,036,141	992	Chr14M	77	31,537,492	808
Chr02M	43	33,477,915	1,046	Chr15P	58	27,993,769	785
Chr03P	134	37,586,275	1,192	Chr15M	51	29,127,640	821
Chr03M	134	46,173,982	1,335	Chr16P	41	31,404,537	939
Chr04P	54	26,277,941	757	Chr16M	65	34,187,610	1,032
Chr04M	22	27,730,151	757	Chr17P	38	30,697,880	821
Chr05P	60	45,221,023	1,261	Chr17M	50	32,333,807	870
Chr05M	53	40,831,580	1,242	Chr18P	44	30,811,384	746
Chr06P	55	34,116,527	953	Chr18M	25	32,208,807	782
Chr06M	52	32,292,431	898	Chr19P	94	30,844,295	888
Chr07P	101	46,598,033	1,217	Chr19M	73	31,834,130	892
Chr07M	52	47,302,989	1,201	Chr20P	75	31,590,913	884
Chr08P	60	30,561,774	922	Chr20M	32	32,252,527	887
Chr08M	53	32,924,569	962	Chr21P	22	26,522,369	825
Chr09P	82	35,593,557	845	Chr21M	29	29,063,070	888
Chr09M	66	35,043,038	844	Chr22P	75	21,424,517	631
Chr10P	35	25,278,516	786	Chr22M	126	31,295,962	724
Chr10M	73	27,482,011	793	Chr23P	74	26,945,570	805
Chr11P	70	28,208,994	759	Chr23M	127	28,155,753	861
Chr11M	86	29,794,645	809	Chr24P	32	25,185,387	670
Chr12P	61	30,469,296	832	Chr24M	28	27,198,397	705
Chr12M	29	28,206,806	750	Chr25P	55	22,243,253	718
Chr13P	37	32,348,885	882	Chr25M	116	26,462,806	759

Total number of contigs: 4,866
Total length of contigs (bp): 1,642,162,944
Total number of anchored contgis: 3,300
Total length of chromosome level assembly (bp): 1,602,357,835
Number of unanchored contigs: 1,566
Length of unanchored contigs (bp): 4,0130,109
Anchor rate (%): 97.56

Supplementary Table 11. Statistical data of 50 chromosomes of *L. capito* .

ChrID	Anchored_ctg	Length (bp)	Gene number	ChrID	Anchored_ctg	Length (bp)	Gene number
Chr01P	20	39,835,672	956	Chr13M	33	33,114,635	814
Chr01M	50	42,091,401	1045	Chr14P	5	32,886,395	750
Chr02P	6	35,565,493	984	Chr14M	15	31,004,809	754
Chr02M	24	35,208,143	1,061	Chr15P	37	31,107,464	720
Chr03P	117	42,086,565	1,126	Chr15M	10	29,997,890	774
Chr03M	192	49,215,915	1,399	Chr16P	21	36,763,600	912
Chr04P	30	31,191,941	828	Chr16M	23	34,809,537	990
Chr04M	40	31,532,706	817	Chr17P	6	31,951,494	781
Chr05P	25	44,041,910	1,121	Chr17M	59	32,300,712	831
Chr05M	61	43,713,097	1,190	Chr18P	19	35,492,216	691
Chr06P	12	35,322,922	902	Chr18M	18	32,333,262	728
Chr06M	4	33,025,491	914	Chr19P	18	33,581,297	837
Chr07P	10	47,916,889	1,096	Chr19M	28	34,958,156	943
Chr07M	21	47,072,773	1,146	Chr20P	26	36,247,472	875
Chr08P	35	34,635,988	914	Chr20M	36	32,042,907	882
Chr08M	12	30,761,087	864	Chr21P	20	29,153,082	781
Chr09P	15	36,394,676	750	Chr21M	19	28,159,712	789
Chr09M	46	36,030,262	841	Chr22P	21	24,330,971	598
Chr10P	17	29,106,581	755	Chr22M	67	37,928,011	846
Chr10M	19	25,709,912	790	Chr23P	19	30,762,945	782
Chr11P	37	30,759,062	713	Chr23M	15	29,349,384	834
Chr11M	42	29,330,057	720	Chr24P	16	28,514,483	643
Chr12P	31	31,293,265	750	Chr24M	14	26,597,859	671
Chr12M	32	29,716,698	756	Chr25P	29	26,367,322	724
Chr13P	30	34,633,884	771	Chr25M	19	26,203,567	731

Total number of contigs: 2,143
Total length of contigs (bp): 1,711,976,228
Total number of anchored contgis: 1,521
Total length of chromosome level assembly (bp): 1,692,151,572
Number of unanchored contigs: 622
Length of unanchored contigs (bp): 19,971,756
Anchor rate (%): 98.83

Supplementary Table 12. BUSCO evaluation of gene completeness from the assembly genomes.

Species	Complete BUSCOs (%)	Complete and single-copy BUSCOs (%)	Complete and duplicated BUSCOs (%)	Fragmented BUSCOs (%)	Missing BUSCOs (%)	Total number of BUSCOs
A. tonkinensis	89.7%	84.1%	5.6%	5.2%	5.1%	4584
An. duoyiheensis	96.7%	90.3%	6.4%	2.0%	1.3%	4584
Ap. chinensis	91.5%	84.4%	7.1%	4.0%	4.5%	4584
B. pengxianensis	87.4%	81.5%	5.9%	5.6%	7.0%	4584
C. oblongus	90.0%	84.6%	5.4%	3.7%	6.3%	4584
D. tumirostris	93.0%	87.2%	5.8%	3.6%	3.4%	4584
G. tungi	95.1%	89.4%	5.7%	3.1%	1.8%	4584
La. taczanowskii	87.6%	81.0%	6.6%	5.5%	6.9%	4584
L. capito	92.6%	33.4%	59.2%	3.7%	3.7%	4584
M. piceus	92.8%	87.1%	5.7%	4.2%	3.0%	4584
Pr. guilingensis	96.1%	89.5%	6.6%	2.3%	1.6%	4584
P. rabaudi	94.4%	31.4%	63.0%	2.9%	2.7%	4584
Ps. parva	88.6%	83.0%	5.6%	3.5%	7.9%	4584
R. sinensis	89.9%	83.7%	6.2%	5.0%	5.1%	4584
Sc. acanthopterus	92.3%	86.7%	5.6%	4.0%	3.7%	4584
Se. prochilus	89.0%	83.5%	5.5%	4.5%	6.5%	4584
Si. rendahli	88.4%	83.0%	5.4%	5.1%	6.5%	4584
S. sinensis	95.6%	23.4%	72.2%	2.5%	1.9%	4584
Sq. curriculus	92.5%	85.7%	6.8%	4.0%	3.5%	4584
T. tinca	88.7%	83.2%	5.5%	4.8%	6.5%	4584
Z. platypus	92.1%	85.9%	6.2%	4.1%	3.8%	4584

Supplementary Table 13. Characteristics of transposable element identified in the assembled genomes.

Species	DNA	LTR	LINE	SINE	RC/Helitron	Unknown	Total (%)
A. tonkinensis	17.03	15.01	4.133	1.231	4.834	1.454	43.692
An. duoyiheensis	31.314	10.535	3.7	0.751	3.271	1.912	51.483
Ap. chinensis	22.617	13.789	4.693	1.241	2.943	1.367	46.65
B. pengxianensis	29.771	12.071	4.804	1.493	1.575	2.087	51.801
C. oblongus	37.807	11.668	3.383	0.407	4.544	1.366	59.175
D. tumirostris	30.539	10.684	2.866	0.24	1.958	2.32	48.607
G. tungi	29.188	7.236	2.436	0.467	2.144	2.738	44.209
La. taczanowskii	34.55	11.5	4.005	0.803	3.155	1.654	55.667
L. capito	23.232	11.989	4.791	0.418	1.836	0.805	43.071
M. piceus	31.149	8.096	2.986	0.446	2.245	1.27	46.192
Pr. guilingensis	28.569	9.299	3.222	0.515	2.157	2.387	46.149
P. rabaudi	25.923	10.219	5.234	0.203	1.097	1.224	43.9
Ps. parva	33.99	14.18	3.56	0.36	2.99	1.8	56.88
R. sinensis	18.225	14.831	4.849	1.804	3.605	1.6	44.914
Sc. acanthopterus	25.343	7.112	4.814	0.621	1.61	1.369	40.869
Se. prochilus	33.911	10.847	3.678	3.422	2.066	0.947	54.871
Si. rendahli	33.763	10.563	3.692	1.121	2.551	0.911	52.601
S. sinensis	27.085	8.548	5.301	0.284	3.989	1.075	46.282
Sq. curriculus	30.722	9.395	3.148	0.348	2	1.409	47.022
T. tinca	24.912	15.262	5.16	0.482	1.593	2.693	50.102
Z. platypus	28.065	9.291	3.703	0.287	2.171	1.277	44.794

Supplementary Table 14. Transposble elements that are subgenome biased in the three allotetraploids.

	TE	SBI	num.P>M	num.P<M	num.P=M
L. capito	L1-128_DR	0.73	25	0	0
	DNAX-18_DR	0.72	1	24	0
	Mariner-4_DR	0.58	0	25	0
	DNA-4-2_DR	0.55	1	23	1
	hAT-N120_DR	0.54	24	1	0
	L1-112_DR	0.52	24	1	0
	TC1DR3	0.49	25	0	0
	hAT-N191_DR	0.46	25	0	0
P. rabaudi	TC1DR2	0.95	0	25	0
	Mariner-12_DR	0.90	25	0	0
	Mariner-N17_DR	0.88	24	0	1
	HATN5_DR	0.86	0	25	0
	DNA-2-19_DR	0.83	0	25	0
	hAT-21_DR	0.79	0	25	0
	DNA-X-8_DR	0.78	0	25	0
	Tc1-7_DR	0.77	0	25	0
	Tc1-8B_DR	0.75	0	25	0
	Mariner-N34_DR	0.75	0	25	0
	Tc1-8_DR	0.70	0	25	0
	hAT-30_DR	0.69	24	0	1
	piggyBac-N22_DR	0.63	24	1	0
	hAT-N91_DR	0.57	0	25	0
	EnSpm-N16_DR	0.56	0	24	1
	SAT-27_DR	0.55	0	25	0
S. sinensis	TDR13B	0.97	25	0	0
	TDR13C	0.92	25	0	0
	Mariner-17_DR	0.89	0	25	0
	LOOPERN4_DR	0.69	0	25	0
	Gypsy-235_DR-LTR	0.63	0	25	0
	DNA-X-6_DR	0.63	0	25	0
	Kolobok-N17_DR	0.63	0	25	0
	Mariner-N34_DR	0.56	0	25	0
	TC1DR2	0.51	0	25	0
	Kolobok-1N1_DR	0.47	0	25	0

Supplementary Table 15. Number of ohnolog clusters in evolutionary fate categories.

	Diploid ancestors	Allotetraploids	Count
neoF	*O. macrolepis*	*L. capito*	272
	O. macrolepis	*P. rabaudi*	420
	O. macrolepis	*S. sinensis*	329
	Sc. acanthopterus	*L. capito*	223
	Sc. acanthopterus	*P. rabaudi*	356
	Sc. acanthopterus	*S. sinensis*	229
nonF	*O. macrolepis*	*L. capito*	226
	O. macrolepis	*P. rabaudi*	333
	O. macrolepis	*S. sinensis*	233
	Sc. acanthopterus	*L. capito*	245
	Sc. acanthopterus	*P. rabaudi*	348
	Sc. acanthopterus	*S. sinensis*	262
subF	*O. macrolepis*	*L. capito*	4
	O. macrolepis	*P. rabaudi*	14
	O. macrolepis	*S. sinensis*	9
	Sc. acanthopterus	*L. capito*	14
	Sc. acanthopterus	*P. rabaudi*	12
	Sc. acanthopterus	*S. sinensis*	11
coexpress	-	*L. capito*	5345
	-	*P. rabaudi*	4884
	-	*S. sinensis*	5305

Supplementary Table 16. Total subgenome percent gene retention values for each subgenome of three focal tetraploid species when aligned to three diploid references.

	L. capito		*P. rabaudi*		*S. sinensis*	
	subP	subM	subP	subM	subP	subM
Danio rerio	85.68	88.09	91.65	92.05	94.03	94.80
O. macrolepis	85.80	88.57	91.26	92.29	93.27	94.85
Sc. acanthopterus	83.98	86.59	88.70	90.26	91.41	93.01

Supplementary Table 17. Detail information of tandem repeat genes of *S. sinensis*, *L. capito* and *P. rabaudi*.

	S. sinensis		*L. capito*		*P. rabaudi*	
	subP	subM	subP	subM	subP	subM
Total arrays	1992	2155	1803	1915	1800	1826
Total Genes	5268	6042	4579	5283	4564	4929
Average array size	2.64	2.8	2.54	2.76	2.53	2.7
Array sizes larger than 5	82	114	52	94	54	79

Supplementary Table 18. Chi-squared test (two sides) results for biased genes in each of six tissue types.

Tissue	Species	Expected subP	Observed subP	Expected subM	Observed subM	X2	df	*P*-value
Brain	*S. sinensis*	387.5	347	387.5	428	8.4658	1	0.003619
Brain	*L. capito*	214	192	214	236	4.5234	1	0.03344
Brain	*P. rabaudi*	387.5	371	387.5	404	1.4052	1	0.2359
Eye	*S. sinensis*	349.5	301	349.5	398	13.461	1	0.000244
Eye	*L. capito*	209	170	209	248	14.555	1	0.000136
Eye	*P. rabaudi*	421	381	421	461	7.601	1	0.005834
Gill	*S. sinensis*	399	363	399	435	6.4962	1	0.01081
Gill	*L. capito*	338	309	338	367	4.9763	1	0.0257
Gill	*P. rabaudi*	448	412	448	484	5.7857	1	0.01616
Heart	*S. sinensis*	481.5	434	481.5	529	9.3718	1	0.002204
Heart	*L. capito*	366	322	366	410	10.579	1	0.001144
Heart	*P. rabaudi*	538.5	445	538.5	632	32.469	1	1.21E-08
Liver	*S. sinensis*	534.5	505	534.5	564	3.2563	1	0.07115
Liver	*L. capito*	333.5	285	333.5	382	14.106	1	0.000173
Liver	*P. rabaudi*	633	560	633	706	16.837	1	4.07E-05
Muscle	*S. sinensis*	439	378	439	500	16.952	1	3.83E-05
Muscle	*L. capito*	253.5	211	253.5	296	14.25	1	0.00016
Muscle	*P. rabaudi*	512	462	512	562	9.7656	1	0.001778

Supplementary Table 19. Chi-squared results (two sides) for biased genes in six tissue types which are retained in a 1:1:2:2:2 ratio across subgenomes and the three focal allotetraploids.

Tissue	Species	Expected subP	Observed subP	Expected subM	Observed subM	X2	df	*P*-value
Brain	*S. sinensis*	153.5	104	153.5	203	31.925	1	1.60E-08
Brain	*L. capito*	92.5	95	92.5	90	0.13514	1	0.7132
Brain	*P. rabaudi*	187	179	187	195	0.68449	1	0.408
Eye	*S. sinensis*	133	92	133	174	25.278	1	4.96E-07
Eye	*L. capito*	106.5	88	106.5	125	6.4272	1	0.01124
Eye	*P. rabaudi*	190.5	168	190.5	213	5.315	1	0.02114
Gill	*S. sinensis*	157	127	157	187	11.465	1	0.000709
Gill	*L. capito*	174.5	176	174.5	173	0.025788	1	0.8724
Gill	*P. rabaudi*	448	412	448	484	2.0973	1	0.1476
Heart	*S. sinensis*	197	154	197	240	18.772	1	1.47E-05
Heart	*L. capito*	201	183	201	219	3.2239	1	0.07257
Heart	*P. rabaudi*	538.5	445	538.5	632	14.824	1	0.000118
Liver	*S. sinensis*	223.5	200	223.5	247	4.9418	1	0.02621
Liver	*L. capito*	196.5	165	196.5	228	10.099	1	0.001483
Liver	*P. rabaudi*	300	260	300	340	10.667	1	0.001091
Muscle	*S. sinensis*	185	144	185	226	18.173	1	2.02E-05
Muscle	*L. capito*	146	118	146	174	10.74	1	0.001049
Muscle	*P. rabaudi*	242.5	231	242.5	254	1.0907	1	0.2963

Supplementary Table 20. Constraint on CNSs located on the subP and subM of each species relative to a model that incorporates mean CNS conservation across all subgenomes and subgenome-specific phylogenetic distance from *O. macrolepis*. Positive values indicate greater constraint than expected, negative values less constraint than expected, and 1SD error bars are derived from the variance in constraint across the subgenome.

Species	Constraint on CNSs relative to a neutral model	1SD
P. rabaudi subP	0.0044259	0.00634195
P. rabaudi subM	-0.0020742	0.00357983
S. sinensis subP	0.0089924	0.00626237
S. sinensis subM	-0.0048886	0.00276531
L. capito subP	-0.0048327	0.00329996
L. capito subM	0.00215342	0.00594526
Glodfish subP	-0.0024434	0.01028964
Glodfish subM	0.00051385	0.00609393
Common carp subP	0.000097932	0.00959718
Common carp subM	-0.0017146	0.0073686

Supplementary Table 21. Non-clonal read pair alignment rate and non-conversion rate of the two diploid and three tetraploid fish species

Sample	Non-clonal read pair alignment (%)	Non-conversion rate (%)
LuC1	49.87	0.22
LuC2	47.54	0.23
LuC3	47.44	0.21
LuC4	47.72	0.23
LuC5	48.16	0.22
PrR1	50.05	0.23
PrR2	50.64	0.22
PrR3	51.83	0.22
PrR4	52.63	0.22
PrR5	49.81	0.22
SpS1	44.35	0.23
SpS2	44.65	0.23
SpS3	45.13	0.23
SpS4	44.51	0.23
SpS5	43.7	0.22
OnM1	55.05	0.24
OnM2	56.98	0.24
OnM3	49.99	0.29
OnM4	52.58	0.34
OnM5	51.81	0.30
ScA1	60.35	0.23
ScA2	57.01	0.23
ScA3	58.86	0.24
ScA4	57.45	0.24
ScA5	56.81	0.24

Abbreviation of species name: Luc, *L. capito* ; PrR, *P. rabaudi* ; SpS, *S. sinensis* ; OnM, *O. macrolepis* ; ScA, *Sc. acanthopterus* .

Supplementary Table 22. TAD and conserved TAD identified by hicFINDTAD and HiTAD.

	S. sinensis	*P. rabaudi*	*L. capito*
TAD (hicFINDTAD)			
SubP_TAD_number	1,333	1,574	1,714
SubM_TAD_number	1,449	1,663	1,669
SubP conserved TAD	1,102	1,282	1,297
SubgM conserved TAD	1,119	1,244	1,332
TAD (HiTAD)			
SubP_TAD_number	1,345	1,292	1,443
SubM_TAD_number	1,493	1,328	1,477
SubP conserved TAD	1,073	1,044	1,069
SubM conserved TAD	1,106	1,059	1,073

Supplementary Table 23. Change in size of TADs found in subgenomes of three allotetraploids.

	S. sinensis subP	*S. sinensis* subM	*P. rabaudi* subP	*P. rabaudi* subM	*L. capito* subP	*L. capito* subM
Maximum size (bp)	3160000	3320000	2120000	5240000	2280000	3200000
Minimum size (bp)	120000	160000	120000	80000	120000	120000
Average size (bp)	578665	594092	471258	466723	473162	482109

Supplementary Table 24. Mitochondrial and assembly genomes of reported species used in this study.

Species	Type	Source	Access number or downable website
Anabarilius grahami	Mitochondrial	NCBI	MF370204.1
	Genome	NCBI	GCA_003731715.1
Ancherythroculter nigrocaudc	Mitochondrial	NCBI	MT588183.1
	Genome	NGDC	GWHAAZV00000000
Carassius auratus	Mitochondrial	NCBI	NC 002079.1
	Genome	NCBI	GCA_014332655.1
			GCA_019720715.2
Ctenopharyngodon idella	Mitochondrial	NCBI	NC 010288.1
	Genome	NCGR	http://www.ncgr.ac.cn/grasscarp/
Cyprinus carpio	Mitochondrial	NCBI	NC 001606.1
	Genome	NCBI	GCA_018340385.1
Danio rerio	Mitochondrial	NCBI	NC 002333.2
	Genome	NCBI	GCA_020184715.1
Danionella translucida	Mitochondrial	GenBase	C_AA001640.1
	Genome	NCBI	GCA_007224835.1
Hypophthalmichthys molitrix	Mitochondrial	NCBI	NC 010156.1
	Genome	figshare	12618884
Hypophthalmichthys nobilis	Mitochondrial	NCBI	NC 010194.1
	Genome	CNSA	CNA0019189
Megalobrama amblycephala	Mitochondrial	NCBI	NC 010341.1
	Genome	GigaDB	100305
Onychostoma macrolepis	Mitochondrial	NCBI	NC 023799.1
	Genome	NCBI	GCA_012432095.1
Paracanthobrama guichenoti	Mitochondrial	NCBI	NC 024430.1
	Genome	NCBI	GCA_018749465.1
Pimephales promelas	Mitochondrial	NCBI	NC 028087.1
	Genome	NCBI	GCA_016745375.1
Poropuntius huangchuchieni	Mitochondrial	NCBI	MN723896.1
	Genome	Dryad	dryad.crjdfn32p
Puntius tetrazona	Mitochondrial	NCBI	EU287909.1
	Genome	NCBI	GCA_018831695.1
Triplophysa bleekeri	Mitochondrial	NCBI	NC 018774.1
	Genome	GigaDB	100823

For *Carassius auratus*, the genome version (GCA_019720715.2) was only used for CNSs analysis, and we used another genome version (GCA_014332655.1) for all other analyses.
