# Supplementary material for: Maternal dominance contributes to subgenome differentiation in allopolyploid fishes
Source: Nat Commun. 2023 Dec 15;14:8357. doi: 10.1038/s41467-023-43740-y (PMC10724154; doi:10.1038/s41467-023-43740-y)
Supplement: Supplementary file 3 — Description of Additional Supplementary Files [file 41467_2023_43740_MOESM3_ESM.pdf]

Description of Additional Supplementary Files

Supplementary Data 1
Description: Evaluation of the accuracy of the assembled genomes using Illumina pair-end reads.

Supplementary Data 2
Description: Characteristics of genes identified in the assembled genomes.

Supplementary Data 3
Description: Detail information of TEs identified in the assembled genomes.

Supplementary Data 4
Description: Hox genes identified in subgenomes of three allotetraploids.

Supplementary Data 5
Description: Occupied genome length, gene number and TE sequences in A/B compartment in subgenomes of three allotetraploids.

Supplementary data 6
Description: Tissues of studied species used for RNA-Seq and Iso-Seq. Supplementary Data 7. Hyperlinks for all data generated in this study.
